# Phytochemical Characterisation and Skin-Relevant In Vitro Biological Activity of Leaf Extracts from Selected *Geranium* Species

**DOI:** 10.3390/molecules31142406

**Published:** 2026-07-08

**Authors:** Maciej Książkiewicz, Emil Paluch, Jarosław Widelski, Justyna Stefanowicz-Hajduk, Kinga Kochan-Jamrozy, Olga Bortkiewicz, Krzysztof Kamil Wojtanowski, Magdalena Gucwa, Judyta Cielecka-Piontek, Elżbieta Studzińska-Sroka

**Affiliations:** 1 Department of Pharmacognosy and Biomaterials, Poznan University of Medical Sciences, 3 Rokietnicka Str., 60-806 Poznan, Poland; ksioze.m@gmail.com (M.K.); jpiontek@ump.edu.pl (J.C.-P.); 2 Department of Microbiology, Faculty of Medicine, Wroclaw Medical University, 4 T. Chałubinskiego Str., 50-376 Wroclaw, Poland; emil.paluch@umw.edu.pl (E.P.); olga.bortkiewicz@umw.edu.pl (O.B.); 3 Department of Pharmacognosy with Medicinal Plants Garden, Lublin Medical University, 20-093 Lublin, Poland; jwidelski@pharmacognosy.home.pl (J.W.); krzysztof.wojtanowski@umlub.pl (K.K.W.); 4 Department of Biology and Pharmaceutical Botany, Medical University of Gdańsk, 107 Al. Gen. J. Hallera Str., 80-416 Gdańsk, Poland; justynastef@gumed.edu.pl (J.S.-H.); kinga.kochan-jamrozy@gumed.edu.pl (K.K.-J.); magdalena.gucwa@gumed.edu.pl (M.G.)

**Keywords:** *Geranium* leaves, plant extracts, antioxidant activity, skin-related enzyme inhibition, antimicrobial activity, cytocompatibility

## Abstract

In the search for natural compounds with skin-relevant biological activity, the leaves from three species of the Geraniaceae family (*Geranium phaeum*, *Geranium sanguineum*, and *Geranium macrorrhizum*) were investigated. The study combined phytochemical characterisation with a broad evaluation of biological activity. The bioactivity of the tested extracts was assessed using antioxidant assays (DPPH, ABTS, and CUPRAC), metal-chelating tests, and inhibition assays of hyaluronidase, elastase, and tyrosinase. In addition, antimicrobial activity was evaluated against selected Gram-positive and Gram-negative bacteria and *Candida* spp., while cytocompatibility was assessed using HaCaT keratinocytes and HFF-1 fibroblasts. To support the interpretation of the observed biological effects, qualitative phytochemical profiling of the extracts was performed by LC–MS; selected compounds were quantified by HPLC; and total polyphenol and flavonoid contents (TPC and TFC) were determined. Overall, the extracts exhibited notable antioxidant, antimicrobial, and enzyme-inhibitory activities, while maintaining good cytocompatibility at lower concentrations. Among the tested species, *G. sanguineum* and *G. macrorrhizum* showed the most pronounced overall activity, likely associated with their high polyphenol content. These results indicate that selected Geraniaceae species represent promising sources of bioactive compounds with combined antioxidant, antimicrobial, and skin-compatible properties, supporting their further investigation in skin-relevant in vitro and formulation-oriented studies.

## 1. Introduction

Plant-derived materials are widely recognised as valuable sources of bioactive compounds relevant to both medicinal and cosmetic skin applications [1]. Owing to their structural diversity, natural origin, and long history of use, plant secondary metabolites are considered promising candidates for the management of chronic, multifactorial disorders, including inflammatory skin diseases [2]. In particular, phenolic compounds, such as flavonoids, tannins, and phenolic acids, are known to exert antioxidant, anti-inflammatory, antimicrobial, and enzyme-modulating effects, all of which are highly relevant to skin homeostasis and pathology [3,4].

Inflammatory skin disorders develop through overlapping mechanisms rather than a single pathological pathway. Oxidative stress, persistent inflammatory responses, altered extracellular matrix turnover, and elevated activity of enzymes such as hyaluronidase, elastase, and tyrosinase all contribute to skin damage and disease progression [5]. In particular, excessive ROS formation may aggravate inflammation, impair the epidermal barrier, and accelerate the degradation of the skin’s structural components. At the same time, hydrolytic and matrix-degrading enzymes may further disturb tissue integrity and delay repair [6]. In this context, plant extracts with combined antioxidant, enzyme-inhibitory, and antimicrobial properties represent an interesting subject for skin-relevant in vitro screening and may provide a basis for further studies on topical formulation development [7].

The family Geraniaceae, which includes seven accepted genera and more than 800 species, is distributed mainly in temperate and subtropical regions. *Geranium* is the largest genus in this family, comprising about 350 species [8]. The name *Geranium* derives from the characteristic fruit shape, which resembles the beak of a crane [9].

**Figure 1:**
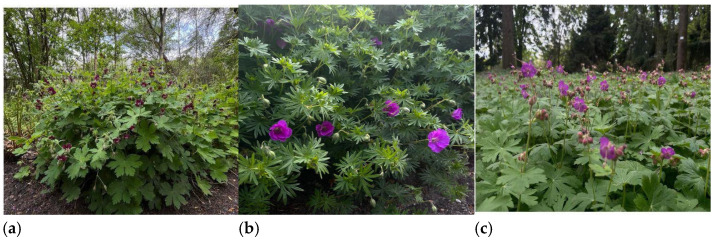
Investigated *Geranium* species: (**a**) *Geranium phaeum* L.; (**b**) *Geranium sanguineum* L.; (**c**) *Geranium macrorrhizum* L. Source: authors’ own photographs taken at the AMU Botanical Garden in Poznań.

The three species investigated in this study differ in their botanical characteristics and distribution. *Geranium phaeum* L. is a perennial species native to Europe, occurring mainly in forests and moist meadows. It forms clumps with branched stems, lobed leaves, and characteristic dark purple-brown flowers [10]. *Geranium sanguineum* L. is a perennial species distributed from Europe to the Caucasus. It has creeping, hairy stems, deeply divided leaves, and purple flowers; its leaves may turn reddish in autumn, which is reflected in its common name [11]. *Geranium macrorrhizum* L. is a rhizomatous, semi-evergreen perennial native to Central and South-Eastern Europe and Western Turkey. It is characterised by aromatic, deeply lobed leaves and pink-to-purple flowers with dark-red calyces [12]. The investigated plant species are shown in Figure 1.

Species of the genus *Geranium* have traditionally been used for their astringent and soothing effects, often prepared as decoctions or rinses for inflammatory conditions of mucous membranes and various skin ailments in ethnomedicine across Europe, particularly in the Balkans. *G. sanguineum* (GS) is specifically documented as a traditional remedy, with its tannin-rich extracts used for their astringent, anti-inflammatory, and antimicrobial effects [13,14]. While *G. macrorrhizum* (GM) also has a long history of medicinal use in the Balkan region, where it is valued as an aromatic and therapeutic plant, ethnobotanical sources associate it with applications for gastrointestinal discomfort, wound care and general tonic effects, consistent with its rich sesquiterpene and phenolic profile. Notably, the literature shows considerable variation in essential oil composition and phenolic content, influenced by environmental factors, and this variability appears to affect antioxidant potential [15]. Although *G. phaeum* (GP) is less frequently described in the ethnomedical literature, reviews of the genus indicate that *Geranium* species broadly share traditional uses linked to their content of flavonoids, proanthocyanidins and tannins, supporting their historical application as astringent and anti-inflammatory agents [16]. Nevertheless, despite increasing interest in the genus as a whole, integrated phytochemical and biological evaluations remain limited for GP, *GS*, and GM, particularly in the context of skin-relevant applications.

In this study, leaves of GP, GS, and GM were investigated for their phytochemical composition and skin-related biological activity. To provide a phytochemical context, total polyphenol content (TPC) and total flavonoid content (TFC) were determined, qualitative profiling was performed by LC–MS, and selected compounds were quantified by HPLC. The extracts were also evaluated for antioxidant activity using DPPH, ABTS, and CUPRAC assays, metal-chelating capacity towards Fe^2+^ and Cu^2+^ ions, and inhibition of hyaluronidase, elastase, and tyrosinase.

## 2. Results and Discussion

### 2.1. Phytochemical Characterisation of Geranium Species Extracts

#### 2.1.1. Total Polyphenol Content (TPC) and Total Flavonoid Content (TFC)

**Figure 2:**
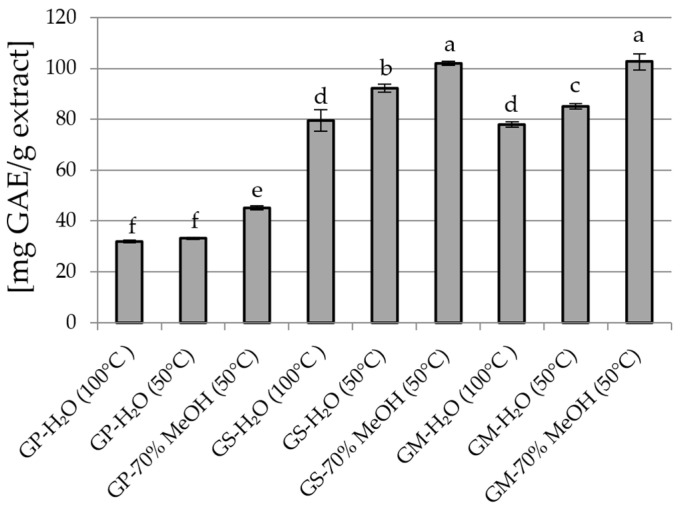
Total polyphenol content in the tested samples, expressed as mg gallic acid equivalent per gram of dry extract [mg GAE/g extract]. GP—*G. phaeum*, GS—*G. sanguineum*, GM—*G. macrorrhizum*. Different letters indicate significant differences between groups (*p* < 0.05, one-way ANOVA followed by Tukey post hoc test).

Determining the polyphenol and flavonoid content in the tested leaf extracts revealed differences depending on both *Geranium* species and the type of solvent/extraction used. The highest polyphenol content was found in the 70% methanol extract from GM leaves (102.65 ± 3.16 mg GAE/g extract) and the 70% methanol extract from GS leaves (102.10 ± 0.75 mg GAE/g extract). The amount of polyphenols in the other extracts ranged between 31.94 ± 0.61 mg GAE/g extract and 92.26 ± 1.49 mg GAE/g extract for the aqueous extract at 100 °C from GP leaves and the aqueous extract at 50 °C from GS leaves, respectively (Figure 2).

The results of the study suggest that the significant content in the tested raw materials is probably due to their polyphenolic structure. The existing literature confirms this conclusion [16]. In a study conducted by Ilić et al., methanol extracts from the aerial parts of *Geraniums* were also characterised by a very high content of polyphenolic compounds. Similarly to our research, GS and GM contained the highest levels of polyphenols, while GP contained significantly lower levels. These were: 547.38 ± 5.83 mg GAE/g extract, 523.96 ± 2.28 mg GAE/g extract, and 170.64 ± 1.08 mg GAE/g extract for extracts from GS, GM, and GP leaves, respectively.

The results obtained by Radulović et al. were closer to ours, with the methanol extract from GM leaves containing 160.2 ± 3.1 mg GAE/g dry weight of the raw material [17].

**Figure 3:**
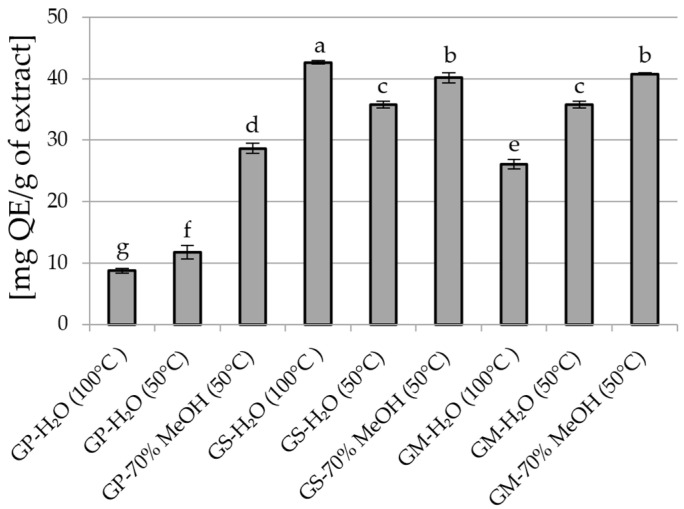
Total flavonoid content in the tested samples, expressed as mg quercetin equivalent per gram of dry extract [mg QE/g of extract]. GP—*G. phaeum*, GS—*G. sanguineum*, GM—*G. macrorrhizum*. Different letters indicate significant differences between groups (*p* < 0.05, one-way ANOVA followed by Tukey post hoc test).

The highest amount of flavonoids was found in the aqueous extract at 100 °C from GS leaves (42.66 ± 0.29 mg QE/g extract), followed by the 70% methanol extract from the leaves of the same plant (40.56 ± 0.90 mg QE/g extract) and the 70% methanol extract from GM leaves (40.45 ± 0.59 mg QE/g extract) (Figure 3). The flavonoid content in the other extracts ranged between 8.74 ± 0.41 mg QE/g extract and 35.83 ± 0.56 mg QE/g extract for the 100 °C aqueous extract from GP leaves and the 50 °C aqueous extract from GS leaves, respectively. The results of this analysis indicated that flavonoids accounted for a high proportion of the polyphenols in the tested raw materials. According to a study conducted by Radulović et al., the methanol extract from GM leaves was characterised by a flavonoid content of 44.9 ± 1.1 mg catechin/g dry weight of the raw material [17]. However, according to a study by Sharopov et al., the methanol extract from GM leaves contained 49.0 mg QE/g [18].

#### 2.1.2. LC–MS Analysis

Analysis of leaf extracts from GP, GS, and GM using LC–MS/MS revealed some differences in the qualitative profile of phenolic metabolites. The extracts were rich in organic acids, gallic acid derivatives, hydrolysable tannins and flavonoids. These results are consistent with previous reports indicating that plants of the genus *Geranium* are rich sources of polyphenolic compounds [16,19].

At the same time, detailed LC–MS/MS profiling demonstrated species-specific differences in metabolite complexity that have not always been described at the qualitative level. These compositional differences may underlie the variability in biological activity observed among the tested extracts.

**Figure 4:**
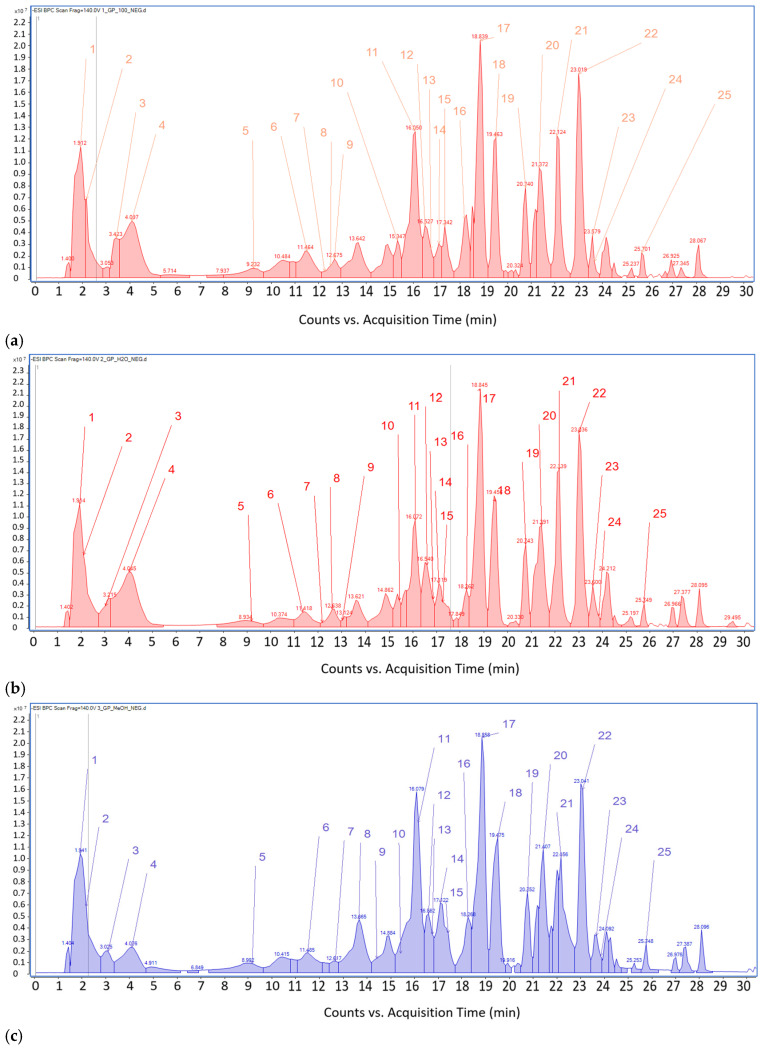
LC–MS chromatograms of three extracts from *Geranium phaeum* (GP): (**a**) GP-H_2_O (100 °C) extract, (**b**) GP-H_2_O (50 °C) extract, and (**c**) GP-70% MeOH (50 °C) extract. Peak numbers correspond to tentatively identified compounds listed in Supplementary Materials Table S1.

Extracts of GP were characterised by a predominance of organic acids and C-glycosyl flavonoids, including apigenin-6,8-di-C-glucoside, vitexin, and isovitexin. While flavonoids in *Geranium* species are well documented, available studies predominantly report flavonol aglycones and their O-glycosides, whereas detailed data on C-glycosyl flavonoids in GP leaves remain limited, and their co-occurrence with numerous phenolic acid derivatives has been rarely reported [20]. Moreover, the detection of cyanidin 3-O-(caffeoyl)-sambubioside is noteworthy, as the available literature on anthocyanins in GP is scarce, and the occurrence of such acylated anthocyanins has not been widely reported for this species. The LC–MS chromatograms of GP extracts are presented in Figure 4.

**Figure 5:**
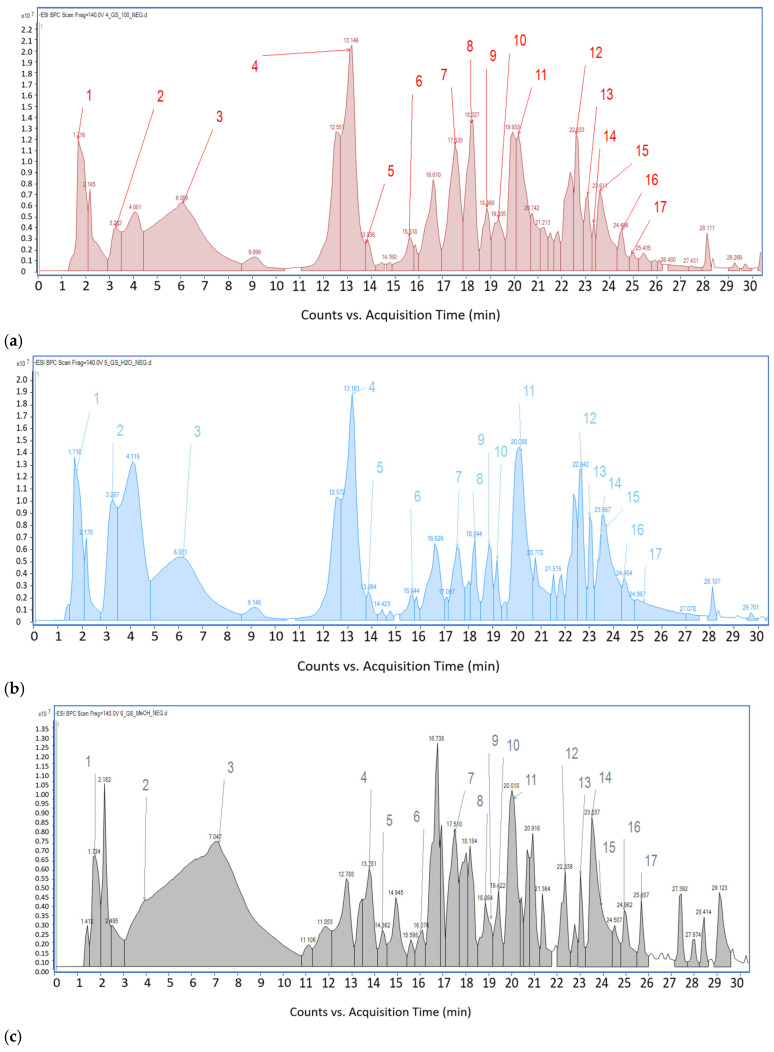
LC–MS chromatograms of three extracts from *Geranium sanguineum* (GS): (**a**) GS-H_2_O (100 °C) extract, (**b**) GS-H_2_O (50 °C) extract, and (**c**) GS-70% MeOH (50 °C) extract. Peak numbers correspond to tentatively identified compounds listed in Supplementary Materials Table S2.

The phenolic profile of GS was dominated by hydrolysable tannins and ellagitannins, including geraniin, which is in agreement with previous studies describing this species as particularly rich in tannin-type polyphenols [21]. Brevifolincarboxylic acid was also identified in the extract as a metabolite of geraniin. However, the present study demonstrated the simultaneous presence of flavonoid glycosides, including rutin and quercetin derivatives, in the same plant material. Such a combination of polyphenolic classes likely contributes to a broad spectrum of biological activities, including antioxidant, anti-inflammatory, and antiviral effects, as reported for GS extracts [22,23]. The LC–MS chromatograms of GS extracts are presented in Figure 5.

**Figure 6:**
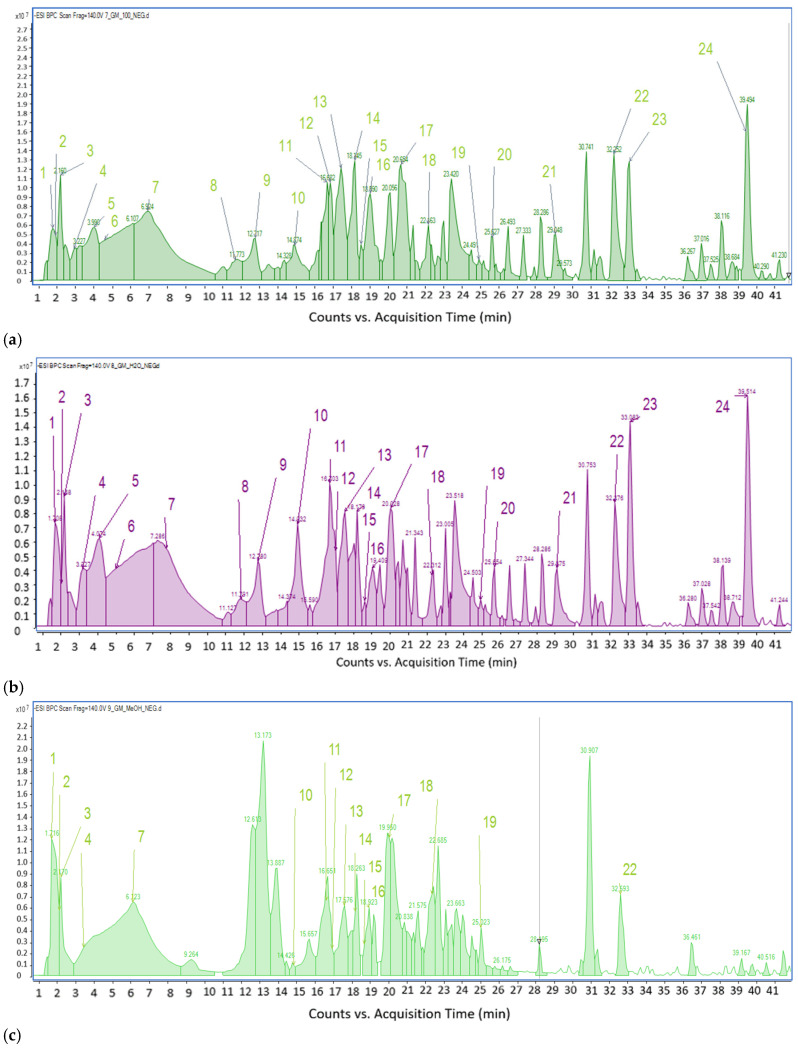
LC–MS chromatograms of three extracts from *Geranium macrorrhizum* (GM): (**a**) GM-H_2_O (100 °C) extract, (**b**) GM-H_2_O (50 °C) extract, and (**c**) GM-70% MeOH (50 °C) extract. Peak numbers correspond to tentatively identified compounds listed in Supplementary Materials Table S3.

Among the analysed species, GM exhibited the most complex phenolic profile. The examined extracts comprise hydrolysable tannins (e.g., corilagin, tellimagrandin I), quinic acid derivatives, and flavonoids. The presence of chlorogenic acid was also confirmed, which is consistent with earlier reports on the chemical composition of this species [24]. Compared to GP and GS, GM showed higher phytochemical complexity. Notably, chlorogenic and neochlorogenic acids were detected exclusively in GM extracts and were absent in the other investigated species. This observation suggests that these compounds may have value as species-associated markers for GM; however, their chemotaxonomic significance requires confirmation in broader studies, including a larger number of samples and populations, as well as related *Geranium* species. The LC–MS chromatograms of GM extracts are presented in Figure 6. MS/MS product ion spectra of *Geranium* sp. extracts are included in the Supplementary Materials (Tables S1–S3 and Figures S1–S3).

From a biological perspective, the identified compounds, particularly hydrolysable tannins, flavonoids, and hydroxycinnamic acid derivatives, are widely associated with strong antioxidant, antimicrobial, and anti-inflammatory activities [16,25,26]. Their co-occurrence within the analysed extracts suggests the possibility of synergistic biological effects, which may partially explain the pronounced bioactivity reported for *Geranium* species in various in vitro and cell-based models.

In summary, the present results confirm previous reports on the phenolic composition of *Geranium* species, provide new qualitative insights into the polyphenolic profile of GP, and highlight the high metabolomic complexity of GM. Comparative analysis revealed a progressive increase in metabolomic complexity from GP, through GS, to GM, which may be relevant for chemotaxonomic and biological studies.

#### 2.1.3. Standardisation of the Extract Using High-Performance Liquid Chromatography (HPLC)

To complement the qualitative LC–MS/MS profiling, selected marker compounds were further quantified by HPLC-DAD. The tested extracts from *Geranium* leaves were subjected to HPLC-DAD analysis, and the contents of four compounds were determined: neochlorogenic acid, chlorogenic acid, gallic acid, and rutin (Table 1). Representative chromatograms of the analysed extracts and references are provided in the Supplementary Materials as Supplementary Figures S4–S16.

The results confirmed the presence of gallic acid in all tested extracts, with its content ranging between 1.41 ± 0.03 mg/g extract and 21.26 ± 0.18 mg/g extract for the aqueous extract at 100 °C from GP leaves and for the aqueous extract at 50 °C from GS leaves, respectively.

In addition, chlorogenic acid was detected in the extracts, with contents of 9.38 ± 0.04 mg/g extract and 11.17 ± 0.28 mg/g extract for GM-H_2_O (100 °C) and GM-H_2_O (50 °C), respectively. These extracts also contained neochlorogenic acid (4.91 ± 0.11 mg/g extract and 3.84 ± 0.07 mg/g extract for GM-H_2_O (100 °C) and GM-H_2_O (50 °C), respectively). Chlorogenic and neochlorogenic acids were not detected in any of the other extracts tested. However, rutin was a flavonoid present in GS and GM extracts but not detected in GP extracts. The amount of the substance varied with the type of extract tested, ranging from 2.59 ± 0.09 mg/g extract to 13.96 ± 0.29 mg/g extract for the aqueous extract at 50 °C from GM leaves and at 100 °C from GS leaves, respectively. These results are consistent with the LC–MS/MS data and further confirm species- and extraction-dependent differences in phenolic composition. In particular, chlorogenic and neochlorogenic acids were detected only in the aqueous GM extracts, whereas they were not detected in GM-70% MeOH or in the other extracts analysed.

In a study by Kremer et al., the rutin content in a methanolic extract of GM herb was reported as 1.116 ± 0.003% (m/m) of dry raw material [27]. Previous studies by Sharopov et al. and Miliauskas et al. confirmed the presence of gallic acid as a major polyphenol [18]. Similarly, Leucuta et al. reported the presence of rutin in GS herb [28].

It should be noted that the observed phytochemical differences reflected both species-related variability and extraction-dependent metabolite recovery. For example, the highest total polyphenol contents were found in the 70% methanol extracts of GM and GS, indicating that hydroalcoholic extraction favoured the recovery of total phenolics from these species. In contrast, the highest flavonoid content was observed in GS-H_2_O (100 °C), followed by GS-70% MeOH and GM-70% MeOH, showing that the recovery of different phenolic groups did not follow a single extraction pattern. Moreover, chlorogenic and neochlorogenic acids were detected only in the aqueous GM extracts, not in GM-70% MeOH, further confirming that individual metabolites may respond differently to solvent type and extraction temperature. These observations indicate that the chemical profiles cannot be interpreted as exclusively species-specific, but rather as the result of both plant species and extraction conditions. Therefore, the relationships between chemical composition and biological activity should be considered preliminary. A more precise interpretation would require targeted quantitative LC–MS/MS analysis of major metabolites, combined with biostatistical correlation analyses and further evaluation of the activity of selected compounds or fractions.

### 2.2. Biological Activity of Geranium Species Extracts

#### 2.2.1. Antioxidant Activity of *Geranium* Species Extracts

Five different in vitro methods were used to determine the antioxidant activity of extracts from the leaves of the tested *Geranium* species. Since excess free radicals contribute to the initiation and maintenance of inflammation, antioxidant compounds reduce the severity of inflammatory processes [29,30]. The ability to remove free radicals indicates the tested substance’s anti-inflammatory potential. The type of reaction that initiates inflammation is the so-called ‘oxidative burst’, which occurs in various cells of the body [31]. The use of various methods allowed for a comprehensive assessment of the properties of the tested extracts.

##### Anti-Radical Activity of *Geranium* Species Extracts

**Figure 7:**
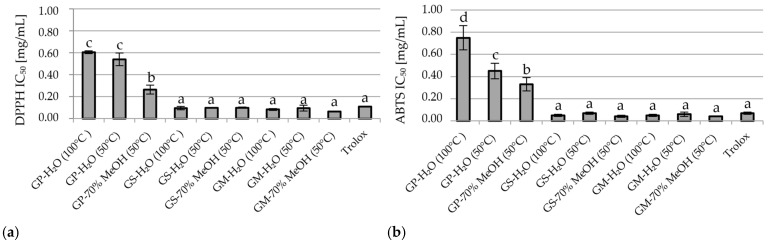
Antiradical activity of *Geranium* sp. extracts: (**a**) DPPH radical scavenging activity; (**b**) ABTS radical scavenging activity (GP—*G. phaeum*, GS—*G. sanguineum*, GM—*G. macrorrhizum*). Different letters indicate significant differences between groups (*p* < 0.05, one-way ANOVA followed by Tukey post hoc test).

The IC_50_ parameter was used to express the results of the DPPH analysis, with a lower value corresponding to greater activity of the tested extract. All extracts from GS and GM leaves were characterised by similar, strong antioxidant activity, and the determined activity ranged from IC_50_ = 0.06 ± 0.00 mg/mL to IC_50_ = 0.10 ± 0.00 mg/mL (for the 70% methanol extract from GM leaves and the aqueous extract at 50 °C from GS leaves, respectively). The observed activity of the *Geranium* leaf extracts was stronger than that of Trolox, the standard (IC_50_ = 0.11 ± 0.01 mg/mL). The extracts that showed lower activity than Trolox were those obtained from GP leaves and ranged from IC_50_ = 0.26 ± 0.04 mg/mL to IC_50_ = 0.60 ± 0.01 mg/mL (for the 70% methanol extract and the aqueous extract at 100 °C, respectively; Figure 7a). The strong antioxidant activity has also been observed by other authors. A study conducted by Sharopov et al. showed that the IC_50_ value for the methanol extract from GM leaves was 14.1 μg/mL [18], while in a study by Radulović et al., the oxidative activity of the methanol extract from GM leaves, expressed as Trolox equivalents, was 178.7 ± 1.8 mg TE/g extract [17]. In turn, an analysis of the 80% methanol extract from GS aerial parts by Nikolova et al. determined the IC_50_ to be 11.95 μg/mL [32].

Similar to the DPPH test, extracts from GS and GM leaves showed similar and strong antioxidant activity (IC_50_ from 0.04 ± 0.00 mg/mL, IC_50_ = 0.07 ± 0.01 mg/mL for 70% methanol extract from GM leaves and for aqueous extract at 50 °C from GS leaves, respectively). The observed activity was greater than that of the standard (Trolox IC_50_: 0.07 ± 0.01 mg/mL). The lowest activity, several times lower than that of the reference substance, was exhibited by extracts from GP leaves. The activity of those extracts ranged from IC_50_ = 0.33 ± 0.06 mg/mL to 0.75 ± 0.11 mg/mL for the 70% methanol extract from GP and for the aqueous extract at 100 °C from GP, respectively (Figure 7b). The high ability to scavenge the ABTS cation radical has already been reported by Sharopov et al. The team’s results indicated that the IC_50_ of the ABTS assay for the methanol extract of GM leaves was 21.2 μg/mL [18]. Meanwhile, a study by Radulović et al. demonstrated the strong antioxidant potential of the methanol extract of GM leaves (323.3 ± 1.2 mg TE/g) in the ABTS assay [17].

##### Chelation Power of *Geranium* Species Extracts

**Figure 8:**
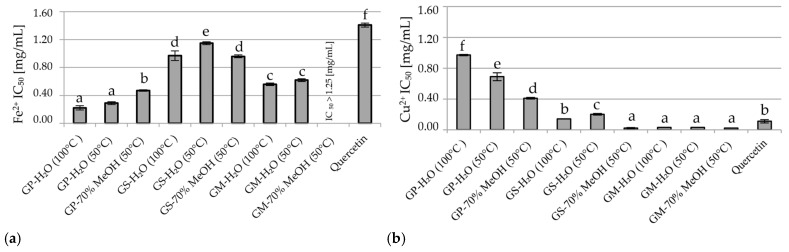
Chelating capacities of *Geranium* sp. extracts: (**a**) Fe^2+^ chelating capacities; (**b**) Cu^2+^ chelating capacities. GP—*G. phaeum*, GS—*G. sanguineum*, GM—*G. macrorrhizum*. Different letters indicate significant differences between groups (*p* < 0.05, one-way ANOVA followed by Tukey post hoc test).

Metal ions have many functions in living organisms. The ability to chelate Fe^2+^ and Cu^2+^ affects antioxidant properties because they facilitate the production of reactive oxygen species [33]. Therefore, our research included an assessment of the ability of the tested extracts to chelate these metal ions. The analyses showed that most of the tested extracts exhibited a high ability to chelate Fe^2+^ (IC_50_ from 0.22 ± 0.03 mg/mL to 1.15 ± 0.02 mg/mL for the aqueous extract at 100 °C from GP leaves and for the aqueous extract at 50 °C from GS leaves, respectively, Figure 8a) higher than the reference substance belonging to the group of flavonoid compounds—quercetin (IC_50_ = 1.41 ± 0.03 mg/mL). The literature indicates that flavonoids have chelating properties, which enable them to interact with transition metals to catalyse electron transport and promote free radical scavenging [34,35].

Another experiment determined copper chelating properties. The most potent extract was the 70% methanol extract from GS leaves (IC_50_ = 0.02 ± 0.01 mg/mL), and all extracts obtained from GM leaves, whose activity ranged between IC_50_ = 0.02 ± 0.00 mg/mL and IC_50_ = 0.03 ± 0.00 mg/mL, for the 70% methanol extract from GM leaves and for the water extract at 50 °C from GM leaves, respectively. The above extracts were characterised by a higher chelating capacity than the reference substance (quercetin; IC_50_ = 0.11 ± 0.02 mg/mL). The activity of the remaining extracts from *Geranium* leaves ranged between IC_50_ = 0.14 ± 0.00 mg/mL and IC_50_ = 0.97 ± 0.01 mg/mL for the aqueous extract at 100 °C from GS leaves and for the aqueous extract at 100 °C from GP leaves, respectively (Figure 8b).

The above studies on the ability to chelate iron and copper ions have not been previously conducted for the species studied. For comparison, a study by Jemia et al. reported that a methanol extract from *Geranium robertianum* showed an iron-chelating ability of approximately 20% [36].

The metal-chelating results did not fully parallel the radical-scavenging assays, indicating that this activity may depend on additional structural features of the extracts. In particular, the relatively strong Fe^2+^-chelating activity of GP extracts, despite their lower TPC, TFC, and quantified phenolic marker content, suggests that compounds other than the selected HPLC markers may be involved. Organic acids and other polar constituents detected in GP by LC–MS/MS may contribute to metal-binding properties. In contrast, the strong Cu^2+^-chelating activity of GS and GM extracts was more consistent with their high polyphenol content and the presence of flavonoids, tannins, and hydroxycinnamic acid derivatives. Therefore, the chelating activity appears to be partly species- and assay-dependent and should not be interpreted solely on the basis of total phenolic content or the four quantified marker compounds.

##### Reducing Power of *Geranium* Species Extracts

The reducing power of plant extracts reflects their ability to donate electrons to oxidants, an important mechanism of antioxidant action that helps mitigate oxidative stress–induced damage in biological systems. Methods based on metal-ion reduction, such as the CUPRAC assay, quantify this electron-transfer capacity by measuring the reduction of Cu^2+^ complexes and are widely used to assess total antioxidant capacity under conditions that are closer to physiological pH than some other assays [37,38].

**Figure 9:**
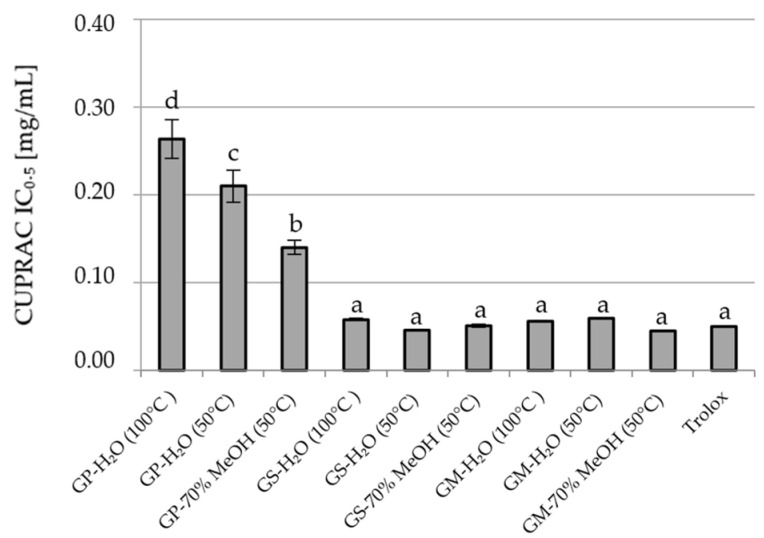
Reducing the power of *Geranium* sp. extracts. GP—*G. phaeum*, GS—*G. sanguineum*, GM—*G. macrorrhizum*. Different letters indicate significant differences between groups (*p* < 0.05, one-way ANOVA followed by Tukey post hoc test).

As in the two previous analyses, the results showed the highest activity across all GS and GM leaf extracts. These values were similar to the activity of Trolox (IC_0.5_ = 0.05 ± 0.00 mg/mL) and ranged between IC_0.5_ = 0.06 ± 0.00 mg/mL and IC_0.5_ = 0.33 ± 0.06 mg/mL for the aqueous extract at 100 °C from GS leaves and for the 70% methanol extract from GM leaves, respectively. Extracts from GP leaves again showed several times lower activity (IC_0.5_ from 0.14 ± 0.01 mg/mL for the 70% methanol extract to 0.26 ± 0.02 mg/mL for the aqueous extract at 100 °C; Figure 9).

The antioxidant potential was also confirmed by the CUPRAC method of Radulović et al. for the methanol extract of GM leaves, which amounted to 466.0 ± 4.1 mg TE/g extract [17].

The studies conducted indicate that extracts from *G. sanguineum* and *G. macrorrhizum* exhibit the strongest antioxidant potential, whereas *G. phaeum* shows lower activity. This may be related to the lower polyphenol content (TPC and TFC). Moreover, the weaker antioxidant response of *G. phaeum* may be related to the lower contribution of highly hydroxylated phenolics and tannin-type constituents, which are important redox-active, electron-donating compounds [39]. In contrast, the high activity of *G. sanguineum* extracts may be associated with their high polyphenol content and the presence of gallic acid and rutin, particularly in the aqueous extracts, as both compounds are known to contribute to radical-scavenging and reducing activity [40,41]. In *G. macrorrhizum*, the strong antioxidant activity of the aqueous extracts may be additionally supported by the presence of chlorogenic and neochlorogenic acids, which were detected only in this species and belong to caffeoylquinic acid derivatives with well-documented antioxidant potential [42]. However, the antioxidant potential cannot be attributed exclusively to the quantified HPLC markers, as the activity of the extracts most likely reflects the combined contribution of phenolic acids, flavonoids, hydrolysable tannins and other LC–MS-detected constituents.

Overall, extracts with higher levels of polyphenolic compounds exhibited greater reducing power than those with lower levels, a trend observed across the antioxidant assays. Studies conducted by Radulović et al. and Sharopov et al. on methanol extract from GM and methanol extract and essential oils from GM, respectively, showed a relationship between polyphenol content and antioxidant activity [17,18]. Similar relationships have also been reported for other natural raw materials.

#### 2.2.2. Enzyme Inhibitory Activity of *Geranium* Species Extracts

##### Hyaluronidase Inhibitory Activity of *Geranium* Species Extracts

Hyaluronidases are common enzymes involved in inflammatory processes, among other functions. Scientific data suggest that inflammation may be associated with the conversion of hyaluronic acid into short-chain fragments [43].

**Figure 10:**
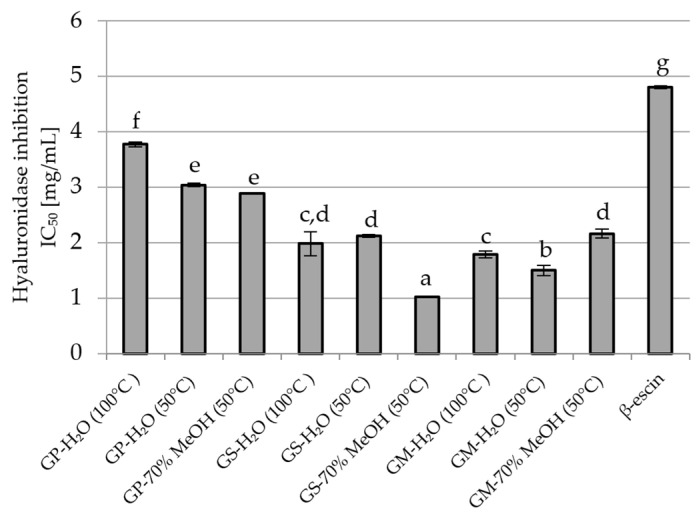
Hyaluronidase inhibition by *Geranium* sp. extracts. GP—*G. phaeum*, GS—*G. sanguineum*, GM—*G. macrorrhizum*. Different letters indicate significant differences between groups (*p* < 0.05, one-way ANOVA followed by Tukey post hoc test).

The results of the studies showed that all tested extracts exhibited greater enzyme-inhibitory capacity than the known hyaluronidase inhibitor β-escin (IC_50_ = 4.80 ± 0.03 mg/mL). The most active was the 70% methanol extract from GS leaves (IC_50_ = 1.02 ± 0.00 mg/mL), and both aqueous extracts from GM leaves were also highly effective (IC_50_ = 1.79 ± 0.06 mg/mL and 1.50 ± 0.09 mg/mL for GM-H_2_O (100 °C) and GM-H_2_O (50 °C) extracts, respectively). The activity of the other extracts ranged from 1.98 ± 0.22 mg/mL to 3.04 ± 0.03 mg/mL for GS-H_2_O (100 °C) and GP-H_2_O (50 °C), respectively (Figure 10).

Based on an analysis by Sklirou et al., an extract from GM (whole plant, including roots) at a concentration of 300 μg/mL inhibited hyaluronidase activity by 51.09% [44].

The hyaluronidase-inhibiting activity also appeared to reflect both the overall phenolic richness and the qualitative profile of polyphenols in the extracts. The strongest activity of GS-70% MeOH and the high activity of GM extracts are consistent with their LC–MS/MS profiles, which revealed tannin-type constituents, flavonoid glycosides and, particularly in GM, hydroxycinnamic acid derivatives. Although rutin, gallic acid, and chlorogenic acid derivatives may contribute to the observed activity, the higher potency of some extracts cannot be explained solely by the quantified markers. This suggests that tannin-type constituents, together with other qualitatively detected polyphenols, may play an important role in hyaluronidase inhibition.

##### Elastase Inhibitory Activity of *Geranium* Species Extracts

**Figure 11:**
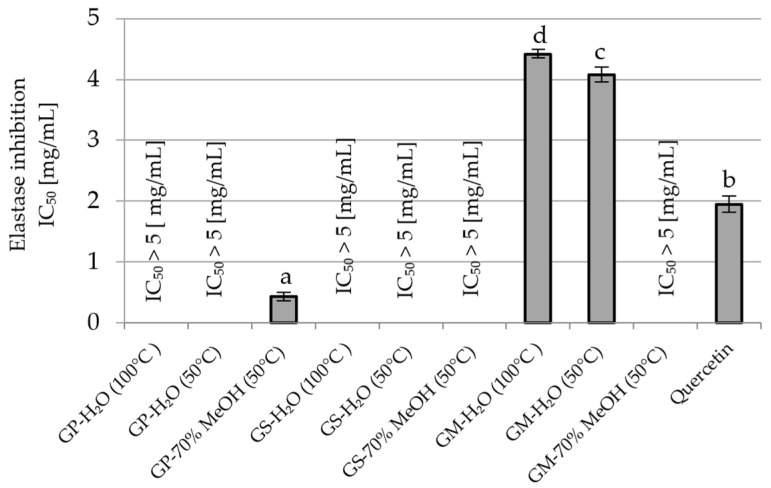
Elastase inhibition by *Geranium* sp. extracts. GP—*G. phaeum*, GS—*G. sanguineum*, GM—*G. macrorrhizum*. IC_50_ > 5 mg/mL indicates that 50% inhibition was not reached within the tested concentration range. Different letters indicate significant differences between groups (*p* < 0.05, one-way ANOVA followed by Tukey post hoc test).

Elastase is responsible for the mechanical properties of connective tissue and plays a key role in inflammatory processes [45]. In the elastase inhibition study, only three of the tested extracts showed sufficient activity to determine their IC_50_ values. The 70% methanol extract from GP leaves proved to be an extract with exceptionally high activity (IC_50_ = 0.43 ± 0.07 mg/mL). The IC_50_ value was approximately 4.5-fold lower than that of quercetin, indicating stronger elastase-inhibiting activity (IC_50_ = 1.95 ± 0.13 mg/mL). The other extracts for which it was possible to determine the IC_50_ parameter were both aqueous extracts from GM leaves (IC_50_ 4.42 ± 0.07 mg/mL, 4.08 ± 0.12 mg/mL for GM-H_2_O (100 °C) and GM-H_2_O (50 °C) extracts, respectively (Figure 11). The other extracts tested did not exceed 50% inhibition of elastase activity at the highest concentration tested (5 mg/mL) and ranged from 16.43 ± 1.92% to 46.82 ± 2.26% for the aqueous extract from GP leaves at 100 °C and from GS leaves at 50 °C, respectively (Table 2). The strong elastase-inhibiting activity of the GP-70% MeOH extract is particularly relevant because it did not follow the same pattern as total phenolic content or the quantified HPLC markers. This suggests the possible involvement of less abundant or more specific constituents detected in the LC–MS/MS profile, including C-glycosyl flavonoids, acylated anthocyanins or other phenolic acid derivatives. However, because the present study used crude extracts, this relationship should be considered preliminary. Further bioactivity-guided fractionation, isolation, and biological testing of individual fractions or purified constituents are needed to identify the compounds responsible for the observed elastase inhibition.

According to studies conducted by Sklirou et al., the whole-plant extract (GM) at a concentration of 300 μg/mL exhibited enzyme inhibition of 46.64% [44]. The difference in the degree of inhibition may result from the method of extraction (the comparative study used pressurised liquid extraction), but also from the type of raw material used. The whole plant, including the roots, was used to obtain the extract.

##### Tyrosinase Inhibitory Activity of *Geranium* Species Extracts

Another enzyme that affects skin function is tyrosinase. It plays a number of roles, including epidermal pigmentation, wound healing and primary immune response [46,47,48].

**Figure 12:**
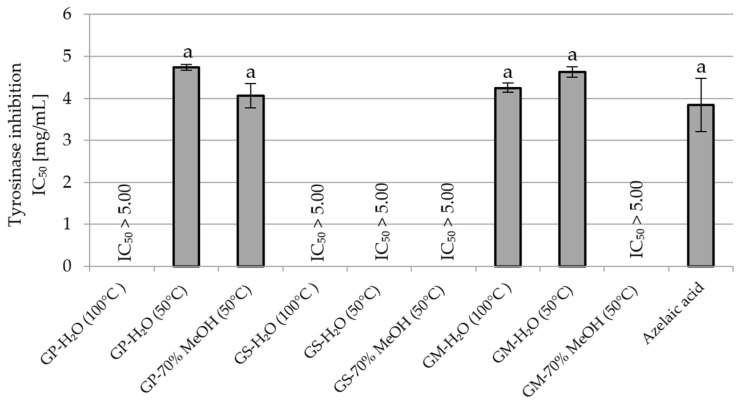
Tyrosinase inhibition of *Geranium* sp. extracts. GP—*G. phaeum*, GS—*G. sanguineum*, GM—*G. macrorrhizum*. IC_50_ > 5 mg/mL indicates that 50% inhibition was not reached within the tested concentration range. Different letters indicate significant differences between groups (*p* < 0.05, one-way ANOVA followed by Tukey post hoc test).

The results of the studies showed that the tested extracts were less effective than the reference substance, azelaic acid (IC_50_ = 3.84 ± 0.63 mg/mL). Only four of the tested extracts showed activity sufficient to determine their IC_50_ values. These values were: IC_50_ = 4.06 ± 0.29 mg/mL, IC_50_ = 4.25 ± 0.11 mg/mL, IC_50_ = 4.63 ± 0.12 mg/mL and IC_50_ = 4.73 ± 0.10 mg/mL for the GP-70% MeOH (50 °C), GM-H_2_O (100 °C), GM-H_2_O (50 °C) and GP-H_2_O (50 °C), respectively (Figure 12). The activity of the other extracts at a concentration of 5 mg/mL ranged between 32.54 ± 4.56% and 47.86 ± 0.99% for the 70% methanol extract from GS leaves and the 70% methanol extract from GM leaves, respectively. In addition, the aqueous extract of GP at 100 °C showed no activity at any of the concentrations tested. The results are presented in Table 3.

According to studies by Ozer et al., the ethanol extract of the aerial parts of GS exhibited enzyme inhibition at 26.37 ± 1.06% [49].

In contrast to the antioxidant assays, tyrosinase inhibition did not show a simple relationship with the content of gallic acid, rutin, or chlorogenic acid derivatives. The highest activity was observed for GP-70% MeOH and GP-H_2_O (50 °C), although GP extracts contained only low amounts of the quantified markers. This suggests that other constituents of GP, including C-glycosyl flavonoids, acylated anthocyanins or other phenolic acid derivatives detected by LC–MS/MS, may be more relevant for this activity. The activity of GM aqueous extracts may additionally be supported by chlorogenic and neochlorogenic acids, but the present data do not allow direct attribution of the effect to these compounds.

Based on enzymatic inhibition results, extracts from the tested *Geranium* species inhibit enzymes involved in inflammatory processes (hyaluronidase), extracellular matrix degradation (elastase), and melanogenesis (tyrosinase). Similar to the antioxidant activity studies, *G. macrorrhizum* demonstrated greater biological potential as an enzyme inhibitor. The weaker potential was presented by *G. sanguineum* and *G. phaeum*. This observation may be important when selecting species for further studies aimed at potential biological applications.

### 2.3. Antimicrobial Properties of Geranium Species Extracts

The escalating global threat of antimicrobial resistance necessitates continued exploration of novel and effective sources of antimicrobial agents [50]. Natural products, including multi-component plant extracts, have long been recognised as valuable reservoirs of bioactive compounds active against a broad range of pathogens and have historically played an important role in medical practice [50,51]. Due to their chemical complexity, such extracts may exhibit enhanced antimicrobial effects through synergistic interactions among constituents, potentially reducing the likelihood of resistance development compared to single-compound therapeutics [52,53]. In this context, the evaluation of the antimicrobial potential of extracts from the genus *Geranium*, several species of which have been traditionally used to treat microbial skin conditions [54], provides a rationale for evaluating their activity against selected skin- and wound-relevant microorganisms. In the present study, this general background was further investigated using three selected species, namely *G. phaeum* (GP), *G. sanguineum* (GS), and *G. macrorrhizum* (GM), in order to evaluate species-specific antimicrobial activity.

Extracts prepared from GP, GS, and GM exhibited measurable antimicrobial activity against both Gram-positive and Gram-negative pathogens implicated in skin and soft-tissue infections (Table 4 and Table 5). Among the Gram-positive strains, *Staphylococcus aureus* and *Streptococcus pyogenes*—well-established etiological agents of superficial and invasive skin infections, including wound infections, cellulitis, impetigo, and erysipelas—showed varying susceptibilities to aqueous and hydroalcoholic *Geranium* preparations. The lowest MIC_50_ values were observed for GP H_2_O (50 °C) and GS H_2_O (50 °C) extracts (0.125–0.5 mg/mL), while the lowest MIC_90_ values were generally recorded for GS and GM extracts.

Gram-negative bacteria, which are also relevant in wound infections, are generally considered more intrinsically resistant due to the presence of an outer membrane [55]. Within this group, GM extracts exhibited the highest overall activity against the tested strains. In particular, the hot-water (100 °C) extract of GM showed the strongest effect against *Pseudomonas aeruginosa*, a pathogen associated with chronic wounds, burn infections, and biofilm formation [56,57], with an MIC_50_ value of 0.0625 mg/mL. Other extracts also inhibited Gram-negative bacteria, with MIC_50_ values ranging from 0.125 to 2 mg/mL depending on the strain and extraction method. Only a few extracts achieved 90% inhibition; the most active in this respect was GP-70% MeOH against *Acinetobacter baumannii* (MIC_90_ = 0.5 mg/mL), a pathogen frequently associated with complicated skin and wound infections [58].

The observed activity across both Gram-positive and Gram-negative bacteria supports the potential applicability of *Geranium* spp. extracts in antimicrobial formulations, particularly for topical use. However, as MBC values were generally higher than MIC values and frequently exceeded the highest tested concentration, the antibacterial effect should be interpreted mainly as growth-inhibitory, i.e., predominantly bacteriostatic rather than bactericidal under the applied experimental conditions.

These findings are consistent with previous reports describing the traditional external use of *Geranium* species in the treatment of wounds, ulcers, and inflammatory conditions of the skin and mucous membranes [54]. The present results provide experimental support for these ethnomedicinal applications and suggest that specific extraction conditions, particularly aqueous decoctions and hydroalcoholic preparations, may enhance the release of antimicrobial constituents active against skin-associated pathogens.

Previous studies have also reported antimicrobial activity within the genus. Methanolic extracts of various *Geranium* species have demonstrated inhibitory effects against *Escherichia coli* and other reference strains [16]. In addition, GS extracts obtained with water or 50% ethanol have shown antibacterial and antifungal activity [8]. Essential oils from related Geraniaceae species, particularly *Pelargonium graveolens*, have also exhibited activity against clinical *S. aureus* strains, including MRSA [59].

It is worth noting that the stronger antibacterial activity of GS and GM may be partly attributable to the presence of phenolic constituents detected in these extracts, including geraniin, chlorogenic acid, rutin, quercetin, and kaempferol derivatives, for which antibacterial or antibiofilm effects have been previously reported [60,61,62,63,64]. However, because the present samples were crude extracts, the observed activity should be interpreted as the combined effect of multiple metabolites rather than the action of a single marker compound.

The extracts were also evaluated for antifungal activity against four *Candida* spp. strains. Across the tested species, the extracts exhibited variable antifungal effects, mainly reflected by growth reduction in the MIC_50_ assay (Table 6).

In general, *Candida glabrata* appeared to be among the more susceptible strains, with the lowest MIC_50_ and MIC_90_ values observed particularly for GS extracts, at 0.03125 mg/mL. High activity was also demonstrated by GM extracts. *Candida tropicalis* showed high susceptibility to GS extracts (MIC_90_ = 0.03125 mg/mL), comparable to GM extracts (MIC_90_ ranging from 0.03125 to 0.0625 mg/mL). In contrast, *C. albicans* and *C. krusei*, both known for their reduced susceptibility to antifungal agents [65,66], were inhibited less effectively. The strongest overall effects against *C. albicans* were observed for GS and GM extracts. Interestingly, particularly high activity against this species was also observed in the 70% MeOH extracts of GP and GM.

Nevertheless, GS and GM extracts, particularly hot-water decoctions, exhibited the lowest MIC_50_ values among all tested preparations, suggesting that these species are the most active and may be especially rich in antifungal constituents effective against non-albicans *Candida* species. It is worth noting that for some *Candida* strains, low MIC_50_ values were accompanied by higher MIC_90_ and MFC values. This indicates that the extracts partially inhibited yeast growth at low concentrations, but complete growth inhibition and fungicidal activity required higher concentrations. Therefore, MIC_50_ values should be interpreted mainly as indicators of partial growth reduction rather than as evidence of complete antifungal activity. Similarly, because MFC values were mostly above the highest tested concentration, the antifungal effect appears to be primarily fungistatic (growth-inhibitory) rather than fungicidal.

These observations are consistent with previous studies indicating that *Geranium* species possess antifungal potential. In particular, aqueous and 50% ethanolic extracts of GS have previously been shown to exhibit antifungal activity, supporting the relevance of polyphenol-rich fractions in inhibiting *Candida* spp. [13]. Although direct studies on GM and GP against *Candida* are limited, several investigations demonstrate that bioactive constituents characteristic of Geraniaceae, especially monoterpenes such as geraniol, exert significant anti-*Candida* and antibiofilm effects [65]. Reported mechanisms include disruption of membrane integrity, inhibition of ergosterol biosynthesis pathways, suppression of virulence factors, and synergy with fluconazole via modulation of efflux pumps [65,67,68,69]. Moreover, essential oils derived from related Geraniaceae species, particularly *Pelargonium graveolens*, have shown inhibitory effects against clinical *C. albicans* isolates, further supporting the antifungal potential of metabolites within this plant family [70].

The outcomes of the present study not only confirm the previously observed properties of plants from the *Geranium* genus, primarily used in traditional medicine, but above all expand existing knowledge through the inclusion of this research on clinically relevant wound pathogens such as *S. aureus*, *S. pyogenes*, *A. baumannii*, and *P. aeruginosa*. In addition, the valuable antifungal effects of GM and GS against *C. glabrata* and *C. tropicalis* further support their potential as sources of biologically active compounds relevant to mucocutaneous and opportunistic infections.

### 2.4. Study on Cell Lines

Assessment of the effects of natural compounds on mammalian cell viability is an essential step in evaluating their potential biological or therapeutic use. Such studies enable the identification of their potential cytotoxic effects and provide basic information on their influence on cellular metabolic activity [71]. In biocompatibility studies of plant compounds/extracts, it is important to determine the concentration that does not cause cytotoxicity in non-cancerous cells. According to the international standard ISO 10993-5 [72], the cytotoxicity threshold is exceeded when cell viability decreases below 70% relative to the control sample. Although this standard was developed for biomaterials and medical devices, the 70% threshold is frequently used as a practical criterion for assessing cytotoxicity in studies of natural substances as well. In the present work, human fibroblasts (HFF-1) and keratinocytes (HaCaT) were employed as in vitro skin models. The *Geranium* extracts were therefore examined using the MTT assay to assess their suitability for further investigation for topical applications, including those targeting inflammatory or hyperproliferative skin conditions.

**Figure 13:**
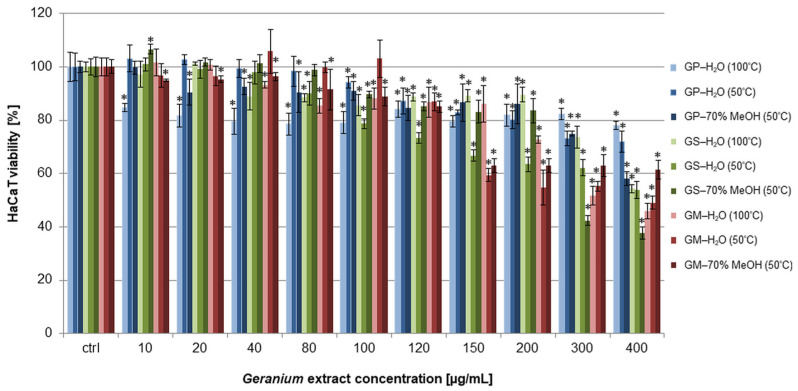
The viability of keratinocytes HaCaT treated with *Geranium* sp. leaf extracts (GP—*G. phaeum*, GS—*G. sanguineum*, GM—*G. macrorrhizum*) for 24 h. The results are presented as mean values ± standard deviations (±SD) obtained with the MTT assay. Error bars indicate ±SD. Statistically significant differences in comparison to control (ctrl—untreated cells/vehicle control) are marked with an asterisk (“*”, Student’s *t*-test, *p* < 0.05).

**Figure 14:**
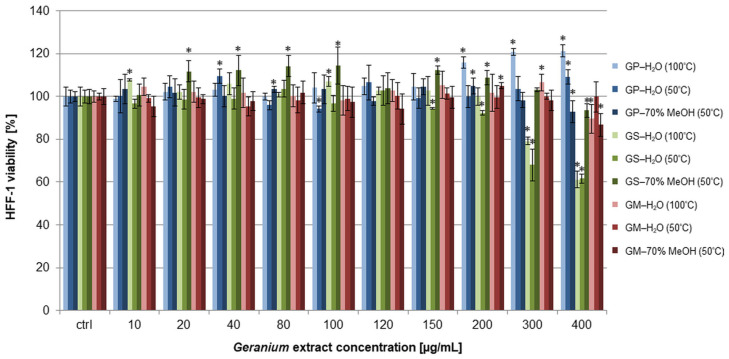
The viability of fibroblasts HFF-1 treated with *Geranium* sp. leaf extracts (GP—*G. phaeum*, GS—*G. sanguineum*, GM—*G. macrorrhizum*) for 24 h. The results are presented as mean values ± standard deviations (±SD) obtained with the MTT assay. Error bars indicate ±SD. Statistically significant differences in comparison to control (ctrl—untreated cells/vehicle control) are marked with an asterisk (“*”, Student’s *t*-test, *p* < 0.05).

The MTT assay indicated that the tested *Geranium* extracts caused concentration-dependent responses in HaCaT keratinocytes and HFF-1 fibroblasts across the range of 10–400 µg/mL, as shown in Figure 13 and Figure 14. Extracts derived from GP exhibited the most favourable profile, as only the hydroalcoholic preparation reduced keratinocyte viability below the cytotoxicity threshold, and this effect was limited to the highest concentration tested (400 µg/mL). In contrast, GS extracts showed more pronounced effects at the highest dose: both aqueous preparations (100 °C and 50 °C) decreased fibroblast and keratinocyte viability to approximately 60%, while the hydroalcoholic GS extract resulted in viability reductions to around 40%, but only in keratinocytes (at 300–400 µg/mL). In fibroblasts, the hydroalcoholic GS extract was not cytotoxic at any of the concentrations tested. Extracts of *G. macrorrhizum* displayed similar trends, with both the 50 °C aqueous and the hydroalcoholic GM extracts beginning to reduce HaCaT viability from concentrations of approximately 150 µg/mL. On the other hand, the GM extracts did not exhibit significant cytotoxic effects on HFF-1 cells within the concentration range used.

**Figure 15:**
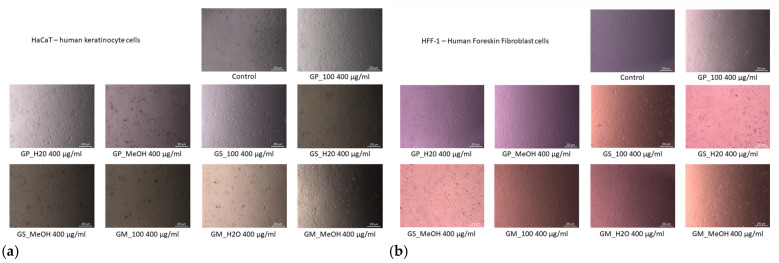
Representative phase-contrast micrographs of cells after exposure to *Geranium* extracts at the highest tested concentration of 400 µg/mL: (**a**) HaCaT human keratinocytes and (**b**) HFF-1 human foreskin fibroblast cells. Untreated cells served as the control. The images show cell morphology after treatment with GP, GS, and GM extracts prepared in water at 100 °C, at 50 °C, or in 70% methanol. Scale bar: 200 µm.

Representative phase-contrast micrographs of HaCaT keratinocytes and HFF-1 fibroblasts treated with the extracts at the highest tested concentration of 400 µg/mL are shown in Figure 15. These images provide additional visual context for the MTT results and illustrate the morphology of cells exposed to the extracts under the most demanding tested conditions.

Across all species, lower and intermediate concentrations generally maintained cell viability at or above 80–90%, demonstrating good cytocompatibility within that range. Aqueous extracts tended to show cytocompatibility similar to, and in some cases slightly better than, hydroalcoholic preparations, particularly in fibroblast cultures. These findings support a preliminary cytocompatibility profile in skin-related in vitro cell models, especially at lower and intermediate concentrations. The present findings, therefore, provide the first in vitro evidence of how extracts of GP, GS, and GM interact with human skin-related cells.

Moreover, studies evaluating the effects of extracts from different *Geranium* species on human cell lines are limited and provide only fragmentary data on their impact on healthy skin cells. The available literature primarily focuses on cytotoxicity against cancer cell lines, and the effect of *Geranium*-derived products appears to be highly dependent on both the plant species and the type of extract [21,73]. On the other hand, published data indicate the biological activity of individual compounds present in the tested extracts against skin cells such as keratinocytes and fibroblasts. For example, the ellagitannin geraniin, identified in the GS extract, was shown by Agyare et al. to increase the metabolic activity of human skin cells (NHDF fibroblasts and HaCaT keratinocytes), thereby promoting cell proliferation [74]. In other studies, the same compound at low concentrations was also shown to protect cells against oxidative stress and H_2_O_2_-induced cell death [75].

Moreover, the relationship between cytocompatibility and antimicrobial activity requires a direct comparison of the tested concentration ranges. The concentration range used in the MTT assay (10–400 µg/mL) corresponded to 0.01–0.4 mg/mL in antimicrobial tests. This comparison showed that antimicrobial activity may be achievable within the concentration range evaluated in HaCaT and HFF-1 cells only for selected extract–microorganism combinations. For bacteria, this applied mainly to MIC_50_ values: GP-H_2_O (50 °C) against *S. aureus* and *S. pyogenes*; GS-H_2_O (50 °C) against *S. pyogenes*; and GM-H_2_O (100 °C) against *P. aeruginosa* (MIC_50_ = 0.0625 mg/mL). In contrast, most bacterial MIC_90_ values exceeded 0.4 mg/mL and therefore cannot be considered supported by the present cytocompatibility data. A more favourable relationship was observed for selected antifungal effects, particularly for GS and GM extracts against *C. glabrata* and *C. tropicalis*, with MIC_90_ values ranging from 0.03125 to 0.0625 mg/mL. Thus, the present results suggest that selected *Geranium* extracts may exert antimicrobial effects at concentrations compatible with MTT-based cytocompatibility in skin-related cells; however, this conclusion is limited to specific extract–microorganism combinations and should be verified in further safety assays.

The tendency observed in our studies offers indications for further research directions. The low cytotoxicity of GP suggests that this species may be a promising source of compounds for further skin-relevant in vitro and formulation-oriented studies, while the stronger effects of GS and GM at higher concentrations suggest the presence of bioactive constituents that can change keratinocyte and fibroblast metabolism. Such activity, particularly when observed only at elevated doses, may be essential for studying the anti-hyperproliferative or anti-inflammatory properties required to treat skin disorders. Collectively, these findings provide a foundation for further studies that are aimed at elucidating the mechanisms of action and evaluating the extracts in more sophisticated dermatological models.

### 2.5. Limitations of the Study

This study has several limitations. First, the plant material was obtained from a botanical garden collection and therefore reflects plants grown under specific and documented cultivation conditions. Since environmental factors may influence secondary metabolite accumulation, the results should be interpreted with caution and should not be directly generalised to natural populations or standardised medicinal raw materials. Second, the enzyme-inhibition assays should be considered preliminary screening studies to select the most active extracts. Further targeted studies should include full concentration–response curves with confidence intervals for IC_50_ values. Third, the observed differences among the biological assays cannot be attributed to a single compound or to total polyphenol/flavonoid content alone, but rather likely reflect assay-dependent contributions of different metabolite classes and the combined action of multiple constituents present in the crude extracts. Further studies should include targeted quantitative analyses, fractionation of the most active extracts, and/or testing of selected isolated compounds to more precisely clarify the relationship between phytochemical composition and biological activity. Finally, no direct anti-inflammatory cellular assays, cytokine measurements, intracellular ROS measurements, skin permeation studies, or mechanistic experiments were performed. Overall, the findings should be considered preliminary and require confirmation in further studies that isolate and characterise the active constituents, clarify their mechanisms of action, and confirm the safety and activity of the most promising extracts in more advanced skin-related experimental models.

## 3. Materials and Methods

### 3.1. Chemical Reagents

Acetate Buffer concentrate was purchased from J.T. Baker (Radnor, PA, USA), and N-Succinyl-Ala-Ala-Ala-p-nitroanilide was purchased from BIOSYNTH s.r.o. (Nobelova, Slovakia). The Folin–Ciocalteu phenol reagent and iron (II) chloride tetrahydrate were from Merck (Darmstadt, Germany). The Ammonium acetate, β-escin, chloral hydrate, dimethyl sulfoxide, 96% ethanol, glacial acetic acid, methanol, copper(II) chloride dehydrate, potassium persulfate, sodium phosphate monobasic dihydrate, sodium phosphate dibasic dodecahydrate, sodium hydroxide, and sodium carbonate were from Avantor Performance Materials Poland S.A. (Gliwice, Poland). All other chemicals were from the Sigma-Aldrich Chemical Co. (Taufkirchen, Germany, or St. Louis, MO, USA). High-quality and ultra-high-quality pure water were prepared using a Direct-Q 3 UV purification system (Merck Millipore, Darmstadt, Germany).

### 3.2. Plant Material

Tested plant material consisted of leaves of three different species: *G. phaeum*, *G. sanguineum*, and *G. macrorrhizum*. The plant material was collected in April 2024 from cultivated specimens growing in the Botanical Garden of Adam Mickiewicz University, Poznań, Poland. After collection, the leaves were dried at room temperature. Voucher specimens are deposited at the Department of Pharmacognosy and Biomaterials, Poznan University of Medical Sciences, under the following numbers: 04_2024_GP, 04_2024_GS, and 04_2024_GM. The collected samples were identified by Grażyna Naser, M. Sc. Eng. (Botanical Garden of Adam Mickiewicz University in Poznan).

### 3.3. Extraction Process

For each of the three *Geranium* species, three types of extracts were prepared: H_2_O (50 °C), H_2_O (100 °C), and 70% MeOH (50 °C). Dried and crushed leaves (2.5 g) were extracted with 50 mL of solvent, corresponding to a solvent-to-material ratio of 20:1 mL/g in each extraction cycle. Water and 70% methanol extractions at 50 °C were performed in an ultrasonic bath (Elmasonic S 180H, Elma, Germany). In addition, water extraction was performed at 100 °C using a conventional water bath (LaboPlay W 215, Bytom, Poland) equipped with a reflux condenser. The extraction process was repeated four times (20 min each cycle). Then, each extract was filtered through cotton wool into a flask. In total, 200 mL samples were obtained. Subsequently, the volume of each extract was reduced using a rotary evaporator (Rotavapor R-210, Büchi, Switzerland), then frozen and freeze-dried. Obtained dry extracts were stored in an airtight Falcon tube secured with Parafilm (room temperature). The yield of the extraction process was presented in Supplementary Materials Table S5.

### 3.4. Phytochemical Studies of the Extracts

#### 3.4.1. Total Polyphenol Content

The analysis was performed following the method developed by Studzińska-Sroka et al. with modifications [76]. In short, 25.0 µL of the test extract or standard was added to each well, followed by 200.0 µL of distilled water, 15.0 µL of the Folin–Ciocalteu reagent, and 60.0 µL of a 20% calcium carbonate solution. A reagent-only control (without extract or standard) was included. Then the plate was shaken in the dark for 30 min at 350 rpm at room temperature, after which absorbance was measured at 760 nm using a Multiskan GOx1510 microplate reader (Thermo Scientific, Vantaa, Finland). All measurements were performed in duplicate, yielding *n* = 4 replicates for extract samples and *n* = 5 for the reference. Results were expressed as milligrams of gallic acid equivalents (GAE) per gram of extract, accompanied by the standard deviation (SD).

#### 3.4.2. Total Flavonoid Content

The analysis was performed following the method of Studzińska-Sroka et al. [76]. Briefly, 100.0 µL of the test extract was mixed with 100.0 µL of a 2% methanolic aluminium chloride solution. After shaking for 1 min, the mixture was incubated in the dark at room temperature for 10 min. Absorbance was then recorded at 415 nm using a Multiskan GOx1510 microplate reader (Thermo Scientific, Vantaa, Finland). A blank sample was prepared by replacing the aluminium chloride solution with methanol. All measurements were carried out in duplicate, yielding *n* = 3 replicates for extract samples and *n* = 5 for the reference. Results are expressed as milligrams of quercetin equivalents (QE) per gram of extract, accompanied by the standard deviation (SD).

#### 3.4.3. LC–MS

The purified samples were analysed qualitatively by an HPLC/ESI–QTOF–MS system in negative ion mode, using a 6530B Accurate-mass QTOF–MS (Agilent Technologies, Inc., Santa Clara, CA, USA) mass spectrometer with an ESI-Jet Stream ion source. The Agilent 1260 chromatograph was equipped with a DAD detector, an autosampler, a binary gradient pump, and a column oven. Gradient of solvents: water with 0,1% formic acid (solvent A) and acetonitrile with 0.1% formic acid (solvent B) were used as the mobile phases. The following gradient procedure was adopted: 0–45 min, 0–60% of B; 45–46 min, 60–95% of B; 46–55 min, 95% of B. The post time was 10 min. The total analysis time was 60 min, with a stable flow rate of 0.300 mL/min. For the stationary phase LunaOmega Polar C18 (100 mm × 2.1 mm, dp 3 μm; Phenomenex, Torrance, CA, USA) was used. The injection volume for the extracts was 10 μL. ESI–QTOF–MS analysis was performed according to the following parameters of the ion source: Dual spray jet stream ESI negative ion mode, gas (N2) flow rate: 12 L/min. nebuliser pressure: 35 psig, vaporiser temp.: 300 °C; *m*/*z* range 100–1000 mass units, with acquisition Mode Auto MS/MS, collision-induced dissociation (CID): 10 and 30 eV with MS scan rate 1 spectrum per s, 2 spectra per cycle, skimmer: 65 V, fragmentor: 140 V and octopole RF Peak: 750 V. The identification was performed based on MS/MS spectra. All compounds were intentionally reported as tentative annotations based on accurate mass measurements, characteristic MS/MS fragmentation patterns, and retention behaviour. Identification was performed using the open-access MS-Dial software (version 5.1.230719; RIKEN, Wako, Japan) and compared with the literature.

#### 3.4.4. Standardisation of the Extract Using High-Performance Liquid Chromatography (HPLC) Analysis

The compounds selected for quantification were chosen based on their clear presence in the HPLC chromatograms and LC–MS profiles, their representation of the main phenolic groups detected in the extracts, their previously reported occurrence in *Geraniaceae* leaves, and the availability of authentic reference standards. Other compounds detected by LC–MS/MS were reported as tentative annotations because reliable quantification would require additional standards and further method development. A high-performance liquid chromatography system (Dionex Thermo Fisher Scientific, Dreieich, Germany) equipped with a high-pressure pump (UltiMate 3000), autosampler (UltiMate 3000), and DAD detector (UltiMate 3000), operated using Chromeleon software (version 7.0; Thermo Fisher Scientific, Waltham, MA, USA), was used to quantify chlorogenic acid, neochlorogenic acid, gallic acid, and rutin in leaf extracts of GM, GP, and GS. Separation was performed on a LiChrospher RP18-5 (E) column (250 mm × 4.6 mm i.d., 5 µm; Supelco, Bellefonte, PA, USA). The analysis followed the method of Paczkowska-Walendowska et al. [77] developed for the determination of phenolic compounds, especially flavonoids and phenolic acids. The procedure was adapted, and analytical performance parameters were established for the reference standards used for quantification. Quantification was performed only for analytes detectable in the investigated extracts and showing retention times comparable with those of the standards. Chromatographic conditions included detection wavelengths of 240 nm for chlorogenic and neochlorogenic acids, 265 nm for gallic acid, and 360 nm for rutin; a flow rate of 1 mL/min; a column temperature of 40 °C; and a mobile phase composed of 0.1% formic acid (solvent A) and acetonitrile (solvent B). The gradient programme was as follows: 0–35 min, 2–20% B; 35–55 min, 20–70% B; 55–60 min, 2% B. Standard solutions were prepared in HPLC-grade methanol at the following concentrations: neochlorogenic acid (0.364 mg/mL), chlorogenic acid (1.0 mg/mL and 0.1 mg/mL), gallic acid (0.1 mg/mL and 0.01 mg/mL), and rutin (0.5 mg/mL). All standards and samples were filtered through 0.45 µm membrane filters prior to injection. Calibration curves were constructed using a range of injection volumes specific to each compound and concentration. Method validation included the assessment of linearity, calibration range, repeatability, intermediate precision, and the determination of the limits of detection (LOD) and quantification (LOQ). The validation parameters are presented in Supplementary Table S4. Chromatograms of the reference standards and examined samples are presented in Supplementary Figures S4–S7 and S8–S16, respectively. Results are expressed as milligrams of compound per gram of extract, accompanied by standard deviation (SD).

### 3.5. Studies of the Biological Activity of Extracts

#### 3.5.1. Antioxidant Activity Assay

##### DPPH Assay

The assay was used to evaluate antiradical activity following the procedure described in reference [78]. Briefly, 25.0 µL of the test extract (concentration range from 0.02 to 0.11 mg/mL for GS and GM, and from 0.078 to 0.63 mg/mL for GP)/standard (Trolox concentration ranged from 0.025 to 0.2 mg/mL) was combined with 175.0 µL of DPPH solution (3.9 mg in 50 mL methanol). The mixture was shaken using a Thermo-Shaker TS-100 microplate shaker (BioSan, Riga, Latvia) at 350 rpm in the dark for 5 min at room temperature, followed by a 25-min incubation in the dark. For each extract concentration, sample blanks containing the extract solution and all assay components except the DPPH solution (MeOH instead) were prepared to correct for possible background absorbance. Blank-corrected absorbance values were used for calculations, and the control was corrected using the corresponding reagent blank without extract. Absorbance was measured at 517 nm using a Multiskan GO 1510 microplate reader (Thermo Fisher Scientific, Vantaa, Finland). Analyses were performed in duplicate for each concentration, and results represent the mean of four measurements (*n* = 4). Antiradical activity is expressed as an IC_50_ value (milligrams per millilitre ± standard deviation). IC_50_ values were calculated only for extracts that reached more than 50% inhibition within the tested concentration range.

##### ABTS Assay

The assay was performed to evaluate antiradical activity following the procedure described in reference [79]. Briefly, 10.0 µL of the test extract (concentration range from 0.025 to 0.2 mg/mL for GS and GM, and from 0.078 to 1.25 mg/mL for GP)/standard (Trolox concentration ranged from 0.025 to 0.2 mg/mL) was mixed with 200.0 µL of ABTS solution, prepared by dissolving 0.1920 g ABTS-(NH_4_)_2_ in 50.0 mL of 0.3311 mg K_2_S_2_O_8_ in 500 mL of distilled water. Prior to use, the ABTS stock solution was diluted to achieve an absorbance of ~0.77 at 734 nm. For each extract concentration, sample blanks containing the extract solution and all assay components except the ABTS solution (water instead) were prepared to correct for possible background absorbance. Blank-corrected absorbance values were used for calculations, and the control was corrected using the corresponding reagent blank without extract. The mixture was shaken in the dark at 500 rpm for 30 min at room temperature, after which absorbance was measured at 734 nm using a Multiskan GO 1510 microplate reader (Thermo Fisher Scientific, Vantaa, Finland). All analyses were performed in duplicate for each concentration, and results are presented as the mean of four measurements (*n* = 4). Antiradical activity is expressed as an IC_50_ value (milligrams per millilitre ± standard deviation). IC_50_ values were calculated only for extracts that reached more than 50% inhibition within the tested concentration range.

##### Chelation Power of Ferrous (Fe^2+^) Ions Assay

The chelating capacity of the extracts was evaluated according to the method of Dinis et al. [80], with minor modifications. Briefly, 200.0 µL of each sample (concentration range from 0.16 to 1.25 mg/mL for all samples)/standard (Quercetin concentration ranged from 0.25 to 5.0 mg/mL) was mixed with 10.0 µL of FeCl_2_·4H_2_O (1 mM) and pre-incubated in a 96-well plate for 10 min at room temperature with shaking (500 rpm). Subsequently, 40.0 µL of ferrozine solution (2.5 mM) was added, followed by incubation for 30 min under the same conditions. For each extract concentration, sample blanks containing the extract solution and all assay components except the ferrozine (water instead) were prepared to correct for possible background absorbance. Blank-corrected absorbance values were used for calculations, and the control was corrected using the corresponding reagent blank without extract. Absorbance was measured at 562 nm in five replicates (*n* = 5). Chelating activity was measured in duplicate for each concentration, and results are presented as the mean of four measurements (*n* = 4). Activity was expressed as IC_50_ value (milligrams per millilitre ± standard deviation). IC_50_ values were calculated only for extracts that reached more than 50% inhibition within the tested concentration range.

##### Chelation Power of Cupric (Cu^2+^) Ions Assay

The chelating capacity of the extracts was evaluated according to the previously described method [81], with minor modifications. Briefly, 30.0 µL of each sample (concentration range from 0.16 to 1.25 mg/mL for all samples)/standard (Quercetin concentration ranged from 0.0625 to 0.5 mg/mL) was mixed with 30.0 µL of CuSO_4_·5H_2_O (0.4 mM) and 175.0 µL of acetate buffer (pH = 6), and then pre-incuibated in a 96-well plate for 10 min at room temperature with shaking (500 rpm). Subsequently, 15.0 µL of pyrocatechol violet (2 mM) was added, followed by 20 min incubation under the same conditions. For each extract concentration, sample blanks containing the extract solution and all assay components except the pyrocatechol violet (buffer instead) were prepared to correct for possible background absorbance. Blank-corrected absorbance values were used for calculations, and the control was corrected using the corresponding reagent blank without extract. Absorbance was measured at 632 nm in five replicates (*n* = 5). Chelating activity was measured in duplicate for each concentration, and results are presented as the mean of four measurements (*n* = 4). Activity was expressed as IC_50_ value (milligrams per millilitre ± standard deviation). IC_50_ values were calculated only for extracts that reached more than 50% inhibition within the tested concentration range.

##### CUPRAC Assay

The assay was conducted according to a previously described procedure [82]. The CUPRAC reagent was prepared by mixing equal volumes of neocuproine (7.5 mM), copper(II) chloride (10 mM), and ammonium acetate buffer (1 M, pH 7.0). Then, 50.0 µL of the test extract (concentration range from 0.01 to 0.16 mg/mL for GS and GM, and from 0.04 to 0.63 mg/mL for GP)/standard (Trolox concentration ranged from 0.00156 to 0.2 mg/mL) was combined with 150 µL of the reagent. For each extract concentration, sample blanks containing the extract solution and all assay components except the CUPRAC reagent (water instead) were prepared to correct for possible background absorbance. Blank-corrected absorbance values were used for calculations, and the control was corrected using the corresponding reagent blank without extract. After incubation in the dark for 30 min at room temperature with shaking (350 rpm), absorbance was recorded at 450 nm. A blank was prepared by replacing the extract with the extraction solvent. The experiment was performed in duplicate for each concentration, and results are presented as the mean of four measurements (*n* = 4). Activity was expressed as the IC_0.5_ value (milligrams per millilitre ± standard deviation), used as an operational comparative parameter, defined as the extract concentration corresponding to an absorbance of 0.5 under the applied assay conditions. Lower IC_0.5_ values indicate stronger reducing/antioxidant capacity. IC_0.5_ values were calculated only for extracts that reached more than 50% inhibition within the tested concentration range.

#### 3.5.2. Inhibition of Enzymes

##### Hyaluronidase Inhibition Assay

The anti-hyaluronidase assay was conducted according to a previously described method [83]. With minor modifications. Briefly, 25.0 µL of incubation buffer, 25.0 µL of hyaluronidase solution (30 U/mL), 10.0 µL of the test extract (concentration range for all samples [mg/mL]: 0.63–5.0 (highest possible concentration to obtain due to extract solubility)/standard (β-escin concentration ranged from 2.5 to 10.0 mg/mL), and 15.0 µL of acetate buffer were added to each well. The mixture was incubated at 37 °C for 15 min with shaking at 200 rpm, after which 25.0 µL of hyaluronic acid (HA) solution was introduced and incubation continued for an additional 45 min under the same conditions. Subsequently, 200.0 µL of cetyltrimethylammonium bromide (CTAB) in 2% NaOH was added, followed by a 10 min incubation at room temperature without shaking. Absorbance was recorded at 600 nm using a Multiskan GO 1510 microplate reader (Thermo Fisher Scientific, Vantaa, Finland). The blank was prepared as described previously [84]. The assay was performed in duplicate for each concentration, with results calculated from four measurements (*n* = 4) and expressed as an IC_50_ value (milligrams per millilitre ± standard deviation). IC_50_ values were calculated only for extracts that reached more than 50% inhibition within the tested concentration range.

##### Elastase Inhibition Assay

The anti-elastase experiment was performed following a previously described method [85] with modifications. Shortly, 20.0 µL of test extract (concentrations for all samples [mg/mL]: 0.005, 0.05, 5.0 (the highest possible concentration to obtain due to extract solubility))/standard (Quercetin concentration ranged from 1.0 to 2.5 mg/mL) and 50 µL of Trisma buffer were added to each well, followed by 10 min room temperature pre-incubation, shaking at 500 rpm. Following, 10 µL of elastase solution was added and incubated for 20 min at room temperature, without shaking. Lastly, 20 µL of N-Succinyl-Ala-Ala-Ala-p-nitroanilide was added to each well, and the wells were incubated at 37 °C for 20 min. For each extract concentration, sample blanks containing the extract solution and all assay components except the enzyme (using buffer instead) were prepared to correct for potential background absorbance. Blank-corrected absorbance values were used for calculations, and the control was corrected using the corresponding reagent blank without extract. Absorbance was recorded at 410 nm using a Multiscan GO 1510 microplate reader (Thermo Fisher Scientific, Vantaa, Finland). The assay was performed in duplicate for each concentration, with results calculated from four measurements (*n* = 4) and expressed as percent inhibition ± standard deviation (SD) for each sample concentration. The IC_50_ value was assessed for standard and extract samples, for which it was feasible (milligrams per millilitre ± standard deviation). IC_50_ values were calculated only for extracts that reached more than 50% inhibition within the tested concentration range.

##### Tyrosinase Inhibition Assay

The anti-tyrosinase assay was performed following a method described by Lim et al. [86] with modifications. Shortly, 25.0 µL of test extract (concentrations for all samples [mg/mL]: 0.32, 1.25, 5.00 (highest possible concentration to obtain due to extract solubility))/standard (azelaic acid concentration ranged from 0.63 to 20.0 mg/mL), 75.0 µL of phosphate buffer (0.1 M, pH 6.8), and 50.0 µL of tyrosinase (192 U/mL solution in phosphate buffer) and were added to each well, followed by 10 min room temperature incubation in dark. Following this, 50.0 µL of L-DOPA solution (2 mM) was added and incubated for 20 min at room temperature, in the dark. For each extract concentration, sample blanks containing the extract solution and all assay components except the substrate (using buffer instead) were prepared to correct for potential background absorbance. Blank-corrected absorbance values were used for calculations, and the control was corrected using the corresponding reagent blank without extract. Lastly, absorbance was recorded at 475 nm using a Multiscan GO 1510 microplate reader (Thermo Fisher Scientific, Vantaa, Finland). The assay was performed in duplicate for each concentration, with results calculated from four measurements (*n* = 4) and expressed as percent inhibition ± standard deviation (SD) for each sample concentration. The IC_50_ value was assessed for standard and extract samples, for which it was feasible (milligrams per millilitre ± standard deviation). IC_50_ values were calculated only for extracts that reached more than 50% inhibition within the tested concentration range.

#### 3.5.3. Study on Antimicrobial Properties

The study employed reference strains of bacteria and yeasts obtained from the laboratory collection and stored at –80 °C or –20 °C, including *Candida albicans* ATCC 10231, *Candida glabrata* ATCC 90030, *Candida krusei* ATCC 6258, *Candida tropicalis* ATCC 750, *Acinetobacter baumannii* ATCC 19606, and *Pseudomonas aeruginosa* ATCC 27853, *Klebsiella pneumoniae* ATCC 700603, *Escherichia coli* ATCC 25922, and *Staphylococcus aureus* ATCC 25923 and *Streptococcus pyogenes* ATCC 16615. All strains were streaked onto solid media, with RPMI 1640 agar for yeasts and AO/AK agar for bacteria, and incubated at 37 °C for 24 h for bacteria and 48 h for yeasts. Stock solutions of *Geranium* extract were prepared in Mueller–Hinton Broth (MHB) at a concentration of 2 mg/mL, and appropriate volumes of the extract and MHB were added to 96-well microplates to obtain final concentrations ranging from 2 to 0.0625 mg/mL for bacteria, and two concentration series for yeasts: 2 to 0.0625 mg/mL in the first series and 0.25 to 0.001953 mg/mL in the second series. The final volume in each well was 200 μL. For each strain, a growth control consisting of 200 μL of inoculated MHB without extract was included. In addition, solvent controls, extract blanks, and sterility controls were used. Extract blanks contained the corresponding extract concentration in medium without microbial inoculum and were used to correct for background absorbance.

Bacterial inocula were prepared by adjusting strain suspensions to 0.5 McFarland in physiological saline (0.9% NaCl). For yeast inocula, a suspension of each *Candida* strain at 0.5 McFarland was prepared in saline, after which 0.1 mL of the suspension was added to 0.9 mL of saline and vortexed for 10 s. Subsequently, 2 μL of the appropriate inoculum was added to each well containing extract, MHB, or control medium. Microplates were incubated at 37 °C with shaking at 400 rpm for 24 h for bacteria and 48 h for yeasts. The final inoculum density was approximately [5 × 10^5^] CFU/mL for bacteria and [2.5 × 10^3^] CFU/mL for yeasts.

After incubation, MIC_90_ values were determined visually by comparing microbial growth in each well with that of the positive control; the lowest extract concentration at which no visible growth was observed was recorded as the MIC_90_. MIC_50_ values were obtained spectrophotometrically by measuring absorbance at 600 nm. To determine minimum bactericidal (MBC) or fungicidal (MFC) concentrations, 5 μL of culture from each well was plated onto solid medium, Mueller–Hinton Agar (MHA) for bacteria and RPMI 1640 agar for yeasts. Plates were incubated at 37 °C for 24 h for bacteria and 48 h for yeasts, and the lowest extract concentration that yielded no growth on solid medium was interpreted as the MBC for bacteria and the MFC for yeasts. Standardly, three replicates were used (3 or 5 if there were discrepancies). The tests were performed in accordance with the recommendations: CLSI M27 for *Candida* and CLSI M07 for bacteria. All media used in the procedures included MHB (Oxoid), RPMI 1640 medium (Thermo Fisher Scientific), and agar-agar (Thermo Fisher Scientific).

#### 3.5.4. In Vitro Cytotoxicity Evaluation in HaCaT and HFF-1 Cells

##### Cell Lines

Human keratinocyte HaCaT and human fibroblast HFF-1 cell lines were obtained from the American Type Culture Collection (ATCC, Manassas, VA, USA) and LGC Standards (Teddington, Middlesex, UK), respectively. Dulbecco’s Modified Eagle’s Medium (DMEM) and Dulbecco’s Modified Eagle’s Medium (DMEM) with high glucose concentration were used for the keratinocytes and the fibroblasts (Merck Millipore, Burlington, MA, USA), respectively. Supplements (100 mg/mL of streptomycin, 100 units/mL of penicillin, and 10% (*v*/*v*) fetal bovine serum (FBS)) were added to all the media. The cells were incubated at 37 °C and 5% CO_2_.

##### MTT [3-(4,5-Dimethylthiazol-2-yl)-2,5-diphenyltetrazolium Bromide] Assay

To assess the viability of HaCaT and HFF-1 cells after treatment with the tested extracts, the MTT assay was used. The cells were seeded at a density of 5 × 10^3^ cells/well and treated with the extracts dissolved in water (2 mg/mL; concentration range: 10–400 µg/mL) for 24 h. The control was the untreated cells (with medium and 40 µL of water). The absorbance of the formazan solution was measured with a microtiter plate reader (Epoch, BioTek Instruments, Winooski, VT, USA). GraFit software v.7 (Erithacus Software, East Grinstead, West Sussex, UK) was used to analyse the results and, if possible, calculate IC_50_ values. MTT assay was performed in six repetitions (*n* = 6). IC_50_ values were not reached within the tested concentration range (10–400 µg/mL). Cell viability ≥70% relative to the vehicle control was interpreted as non-cytotoxic, whereas viability below 70% was considered indicative of cytotoxicity, in accordance with the commonly applied ISO 10993-5 criterion [72].

### 3.6. Statistical Analysis

Microsoft Excel (Microsoft Office LTSC 2021 Professional Plus, Microsoft Corp., Redmond, WA, USA) was used to calculate mean values and standard deviations. Statistical significance was assessed using Statistica software (version 13.3; StatSoft Polska Sp. z o.o., Krakow, Poland). Data are expressed as mean ± standard deviation (SD). IC_50_/IC_0.5_ values were calculated from the concentration–response curve. Statistically significant differences in comparison to the control are marked with an asterisk (“*”, Student’s *t*-test, *p* < 0.05). Different letters indicate statistically significant differences between extracts/samples according to one-way ANOVA followed by Tukey’s post hoc test (*p* < 0.05).

## 4. Conclusions

The present study indicates that leaf extracts from the investigated *Geranium* species may represent promising sources of bioactive compounds with skin-relevant in vitro biological activity. This was particularly evident in *G. sanguineum* and *G. macrorrhizum*, which exhibited notable antioxidant activity, likely attributable to their high polyphenol content and polyphenol-rich phytochemical profiles. Selected extracts also exhibited inhibitory activity against hyaluronidase, elastase, and tyrosinase, supporting their selection for further skin-related studies. In this context, *G. macrorrhizum* extracts and the GP-70% MeOH extract appear particularly noteworthy.

Moreover, extracts from *G. macrorrhizum* and *G. sanguineum* showed greater antimicrobial activity against bacteria and *Candida* species than *G. phaeum*, whereas *G. phaeum* exhibited lower antimicrobial activity but a more favourable cytocompatibility profile. All tested extracts were non-cytotoxic at low and intermediate concentrations in keratinocyte and fibroblast models. The partial overlap between selected antimicrobial effects and non-cytotoxic concentration ranges indicates the practical relevance of these findings for prioritising extracts for further safety, activity, and formulation-oriented studies. Overall, *G. macrorrhizum* and *G. sanguineum* appear to be the most promising sources of bioactive compounds among the investigated species.

## Figures and Tables

**Table 1:** Content of selected active compounds in extracts from the leaves of three species of *Geranium*.

Extract	Detected Amount [mg/g of Extract]			
	Neochlorogenic Acid	Chlorogenic Acid	Gallic Acid	Rutin
GP-H_2_O (100 °C)	nd	nd	1.41 ± 0.03	nd
GP-H_2_O (50 °C)	nd	nd	2.83 ± 0.01	nd
GP-70% MeOH (50 °C)	nd	nd	1.84 ± 0.11	nd
GS-H_2_O (100 °C)	nd	nd	6.13 ± 0.29	13.96 ± 0.29
GS-H_2_O (50 °C)	nd	nd	21.26 ± 0.18	10.75 ± 1.91
GS-70% MeOH (50 °C)	nd	nd	1.45 ± 0.03	4.19 ± 0.24
GM-H_2_O (100 °C)	4.91 ± 0.11	9.38 ± 0.04	4.99 ± 0.01	2.89 ± 0.05
GM-H_2_O (50 °C)	3.84 ± 0.07	11.17 ± 0.28	8.74 ± 0.25	2.59 ± 0.09
GM-70% MeOH (50 °C)	nd	nd	1.63 ± 0.00	12.31 ± 0.06

GP—*G. phaeum*, GS—*G. sanguineum*, GM—*G. macrorrhizum*; nd—not detected.

**Table 2:** Elastase inhibition capacity (%) of *Geranium* sp. extracts at concentrations of 5.0, 0.05, and 0.005 mg/mL.

	Elastase Inhibition Capacity [%]		
Extract [mg/mL]	5.00	0.05	0.005
GP-H_2_O (100 °C)	16.43 ± 1.92 ^g^	7.42 ± 1.01 ^e^	n.a.
GP-H_2_O (50 °C)	29.63 ± 2.23 ^f^	13.93 ± 0.72 ^c,d^	1.85 ± 0.06 ^b,c^
GP-70% MeOH (50 °C)	79.20 ± 1.50 ^a^	14.46 ± 3.57 ^c^	4.92 ± 1.43 ^b^
GS-H_2_O (100 °C)	45.59 ± 1.15 ^d^	21.14 ± 0.73 ^a,b^	15.95 ± 0.91 ^a^
GS-H_2_O (50 °C)	46.82 ± 2.26 ^d^	21.78 ± 2.78 ^a^	13.68 ± 2.06 ^a^
GS-70% MeOH (50 °C)	37.43 ± 2.06 ^e^	15.53 ± 1.06 ^b,c^	1.81 ± 0.19 ^b,c^
GM-H_2_O (100 °C)	53.34 ± 1.66 ^c^	18.41 ± 1.82 ^a,b,c^	2.84 ± 3.36 ^b,c^
GM-H_2_O (50 °C)	58.75 ± 1.62 ^b^	13.38 ± 4.11 ^c,d^	0.14 ± 2.07 ^c^
GM-70% MeOH (50 °C)	26.33 ± 1.53 ^f^	8.51 ± 1.16 ^d,e^	n.a.

GP—*G. phaeum*, GS—*G. sanguineum*, GM—*G. macrorrhizum*; n.a.—not active at the tested concentration. Different letters indicate significant differences between groups (*p* < 0.05, one-way ANOVA followed by Tukey post hoc test).

**Table 3:** Tyrosinase inhibition capacity (%) of *Geranium* sp. extracts at concentrations of 5.00, 1.25, and 0.32 mg/mL.

Extract [mg/mL]	Tyrosinase Inhibition Capacity [%]		
	5.00	1.25	0.32
GP-H_2_O (100 °C)	n.m.	18.78 ± 4.66 ^a,b,c^	12.28 ± 7.90 ^a^
GP-H_2_O (50 °C)	53.69 ± 1.08 ^a,b^	2.59 ± 3.60 ^d^	n.a.
GP-70% MeOH (50 °C)	60.57 ± 4.02 ^a^	21.34 ± 2.35 ^a,b^	12.25 ± 5.79 ^a^
GS-H_2_O (100 °C)	37.08 ± 3.20 ^d,e^	18.94 ± 0.21 ^a,b,c^	0.88 ± 1.40 ^b,c^
GS-H_2_O (50 °C)	32.54 ± 4.56 ^e^	12.98 ± 0.85 ^c^	n.a.
GS-70% MeOH (50 °C)	40.97 ± 1.84 ^c,d^	24.78 ± 1.13 ^a^	6.18 ± 0.75 ^a,b,c^
GM-H_2_O (100 °C)	57.90 ± 1.10 ^a^	19.03 ± 1.87 ^a,b,c^	3.62 ± 2.10 ^a,b,c^
GM-H_2_O (50 °C)	53.49 ± 1.39 ^a,b^	17.34 ± 1.23 ^b,c^	6.75 ± 1.8 ^a,b,c^
GM-70% MeOH (50 °C)	47.86 ± 0.99 ^b,c^	25.58 ± 2.69 ^a^	10.89 ± 0.84 ^a,b^

GP—*G. phaeum*, GS—*G. sanguineum*, GM—*G. macrorrhizum*; n.a.—not active at the tested concentration; n.m.—not marked at this concentration. Different letters indicate significant differences between groups (*p* < 0.05, one-way ANOVA followed by Tukey post hoc test).

**Table 4:** Antimicrobial activity of *Geranium* sp. extracts against selected Gram-positive bacterial species.

Extract	*Staphylococcus aureus*			*Streptococcus pyogenes*		
	MIC_50_	MIC_90_	MBC	MIC_50_	MIC_90_	MBC
	[mg/mL]			[mg/mL]		
GP-H_2_O (100 °C)	0.125	>2	>2	1.75	>2	>2
GP-H_2_O (50 °C)	0.125	>2	>2	0.125	>2	>2
GP-70% MeOH (50 °C)	1.5	1.75	>2	1	1	1
GS-H_2_O (100 °C)	0.5	0.5	>2	0.5	0.5	1
GS-H_2_O (50 °C)	0.5	1	>2	0.125	0.5	2
GS-70% MeOH (50 °C)	1	1	>2	1.5	1.5	1.75
GM-H_2_O (100 °C)	1	1	>2	1	1	1.75
GM-H_2_O (50 °C)	1	1	>2	1	1	2
GM-70% MeOH (50 °C)	0.5	0.5	>2	0.5	0.5	1

GP—*G. phaeum*, GS—*G. sanguineum*, GM—*G. macrorrhizum*.

**Table 5:** Antimicrobial activity of *Geranium* sp. extracts against selected Gram-negative bacterial species.

Extract	*Acinetobacter baumannii*			*Klebsiella pneumoniae*			*Pseudomonas aeruginosa*			*Escherichia coli*		
	MIC_50_	MIC_90_	MBC	MIC_50_	MIC_90_	MBC	MIC_50_	MIC_90_	MBC	MIC_50_	MIC_90_	MBC
	[mg/mL]			[mg/mL]			[mg/mL]			[mg/mL]		
GP-H_2_O (100 °C)	1.75	>2	>2	1	>2	>2	1.5	>2	>2	0.25	>2	>2
GP-H_2_O (50 °C)	0.5	>2	>2	0.25	>2	>2	0.25	>2	>2	2	>2	>2
GP-70% MeOH (50 °C)	0.5	0.5	>2	0.25	>2	>2	1	1	>2	1	1	>2
GS-H_2_O (100 °C)	0.125	>2	>2	0.25	>2	>2	0.125	>2	>2	1	>2	>2
GS-H_2_O (50 °C)	1.75	>2	>2	1	>2	>2	1.5	>2	>2	0.5	>2	>2
GS-70% MeOH (50 °C)	0.25	2	>2	0.125	>2	>2	1	>2	>2	0.5	>2	>2
GM-H_2_O (100 °C)	0.25	2	>2	0.25	>2	>2	0.0625	>2	>2	0.25	>2	>2
GM-H_2_O (50 °C)	2	2	>2	0.5	>2	>2	1	>2	>2	0.25	>2	>2
GM-70% MeOH (50 °C)	0.125	>2	>2	0.125	>2	>2	0.25	>2	>2	0.25	>2	>2

GP—*G. phaeum*, GS—*G. sanguineum*, GM—*G. macrorrhizum*.

**Table 6:** Antifungal activity of *Geranium* sp. extracts against selected *Candida* species.

Extract	*Candida albicans*			*Candida glabrata*			*Candida krusei*			*Candida tropicalis*		
	MIC_50_	MIC_90_	MFC	MIC_50_	MIC_90_	MFC	MIC_50_	MIC_90_	MFC	MIC_50_	MIC_90_	MFC
	[mg/mL]			[mg/mL]			[mg/mL]			[mg/mL]		
GP-H_2_O (100 °C)	2	>2	>2	2	2	>2	0.003905	>2	>2	1.75	1.75	>2
GP-H_2_O (50 °C)	2	>2	>2	1.75	1.75	>2	0.0625	>2	>2	1.5	1.75	>2
GP-70% MeOH (50 °C)	0.001953	1.75	>2	1.75	2	>2	1	1	>2	0.125	0.125	>2
GS-H_2_O (100 °C)	0.125	2	>2	0.03125	0.03125	>2	2	2	>2	0.03125	0.03125	>2
GS-H_2_O (50 °C)	0.5	1.75	>2	0.03125	0.03125	>2	0.125	0.5	>2	0.01562	0.03125	>2
GS-70% MeOH (50 °C)	0.25	1	>2	0.03125	0.03125	>2	0.125	0.125	>2	0.03125	0.03125	>2
GM-H_2_O (100 °C)	0.5	1	>2	0.03125	0.03125	>2	0.5	0.5	>2	0.03125	0.03125	>2
GM-H_2_O (50 °C)	0.01562	>2	>2	0.5	0.5	>2	0.00391	1.5	>2	0.0625	0.0625	>2
GM-70% MeOH (50 °C)	0.00781	1.5	>2	0.03125	0.03125	>2	0.125	1	>2	0.03125	0.03125	>2

GP—*G. phaeum*, GS—*G. sanguineum*, GM—*G. macrorrhizum*.

## Data Availability

All data supporting reported results can be found within the manuscript or Supplementary Materials.

## Supplementary Materials

The following supporting information can be downloaded at https://www.mdpi.com/article/10.3390/molecules31142406/s1, Figure S1: MS/MS product ion spectra obtained by LC–MS/MS analysis of extracts from *Geranium phaeum*; Figure S2: MS/MS product ion spectra obtained by LC–MS/MS analysis of extracts from *Geranium sanguineum*; Figure S3: MS/MS product ion spectra obtained by LC–MS/MS analysis of extracts from *Geranium macrorrhizum*; Figure S4: Chromatogram of gallic acid (Rt = 9.117 min; 265 nm); Figure S5: Chromatogram of neochlorogenic acid (Rt = 16.717 min; 240 nm); Figure S6: Chromatogram of chlorogenic acid (Rt = 22.713 min; 240 nm); Figure S7: Chromatogram of rutin (Rt = 37.250 min; 360 nm); Figure S8: Chromatogram of the aqueous extract (100 °C) from *G. phaeum* leaves; gallic acid (Rt = 9.133 min); Figure S9: Chromatogram of the aqueous extract (50 °C) from *G. phaeum* leaves; gallic acid (Rt = 9.123 min); Figure S10: Chromatogram of the 70% methanol extract from *G. phaeum* leaves; gallic acid (Rt = 9.127 min); Figure S11: Chromatogram of the aqueous extract (100 °C) from *G. sanguineum* leaves; gallic acid (Rt = 9.117 min), rutin (Rt = 37.177 min); Figure S12: Chromatogram of the aqueous extract (50 °C) from *G. sanguineum* leaves; Gallic acid (Rt = 9.120 min), rutin (Rt = 37.197 min); Figure S13: Chromatogram of the 70% methanol extract from *G. sanguineum* leaves; Gallic acid (Rt = 9.127 min), rutin (Rt = 37.237 min); Figure S14: Chromatogram of the aqueous extract (100 °C) from *G. macrorrhizum* laves; Galiic acid (Rt = 9.110 min); neochlorogenic acid (Rt = 16.439 min); chlorogenic acid (Rt = 22.613 min); rutin (Rt = 37.250 min); Figure S15: Chromatogram of the aqueous extract (50 °C) from *G. macrorrhizum* leaves; Gallic acid (Rt = 9.120 min); neochlorogenic acid (Rt = 16.463 min); chlorogenic acid (Rt = 22.653 min); rutin (Rt = 37.210 min); Figure S16: Chromatogram of the 70% methanol extract from *G. macrorrhizum* leaves; Gallic acid (Rt = 9.120 min); rutin (Rt = 37.177 min); Figure S17: Standard and response curves for DPPH assay (standard—Trolox concentration range 0.025–0.2 mg/mL; y = 314.05x − 7.8686; r = 0.9998); Figure S18: Standard and response curves for ABTS assay (standard: Trolox; concentration range 0.025–0.2 mg/mL; y = 356.1x + 24.827; r = 0.9994); Figure S19: Standard and response curves for: Fe^2+^ chelation (standard: Quercetin; concentration range 0.25–5.0 mg/mL; y = 10.985x + 34.533; r = 0.9897); Figure S20: Standard and response curves for Cu^2+^ chelation assay (standard: Quercetin; concentration range 0.0625–0.5 mg/mL; y = 31.929ln(x) + 121.24; r = 0.9972); Figure S21: Standard and response curves for CUPRAC assay (standard: Trolox; concentration range 0.0156–0.2 mg/mL; y = 8.2943x + 0.0936; r = 0.9998). Table S1: Results of LC-MS/MS analysis of *Geranium phaeum* (GP) extracts; Table S2: Results of LC-MS/MS analysis of *Geranium sanguineum* (GS) extracts; Table S3: Results of LC-MS/MS analysis of *Geranium macrorrhizum* extracts; Table S4: Validation parameters of the HPLC method used for quantitative analysis of selected phenolic compounds; Table S5: Extraction yields and sample nomenclature of *Geranium* leaf extracts.

## References

[1] Michalak M. (2023). Plant Extracts as Skin Care and Therapeutic Agents. Int. J. Mol. Sci..

[2] Farhan M. (2024). The Promising Role of Polyphenols in Skin Disorders. Molecules.

[3] Čižmárová B., Hubková B., Tomečková V., Birková A. (2023). Flavonoids as Promising Natural Compounds in the Prevention and Treatment of Selected Skin Diseases. Int. J. Mol. Sci..

[4] Mattosinhos P.d.S., Sarandy M.M., Novaes R.D., Esposito D., Gonçalves R.V. (2022). Anti-Inflammatory, Antioxidant, and Skin Regenerative Potential of Secondary Metabolites from Plants of the Brassicaceae Family: A Systematic Review of In Vitro and In Vivo Preclinical Evidence (Biological Activities Brassicaceae Skin Diseases). Antioxidants.

[5] Briganti S., Picardo M. (2003). Antioxidant Activity, Lipid Peroxidation and Skin Diseases. What’s New. J. Eur. Acad. Dermatol. Venereol..

[6] Pittayapruek P., Meephansan J., Prapapan O., Komine M., Ohtsuki M. (2016). Role of Matrix Metalloproteinases in Photoaging and Photocarcinogenesis. Int. J. Mol. Sci..

[7] Działo M., Mierziak J., Korzun U., Preisner M., Szopa J., Kulma A. (2016). The Potential of Plant Phenolics in Prevention and Therapy of Skin Disorders. Int. J. Mol. Sci..

[8] Aedo C., Pando F. (2017). A Distribution and Taxonomic Reference Dataset of *Geranium* in the New World. Sci. Data.

[9] Royal Botanic Gardens, Kew Geraniaceae Juss. https://powo.science.kew.org/taxon/urn:lsid:ipni.org:names:30001521-2/general-information.

[10] Royal Botanic Gardens, Kew Plants of the World Online. *Geranium phaeum* L.. https://powo.science.kew.org/taxon/urn:lsid:ipni.org:names:373470-1.

[11] Royal Botanic Gardens, Kew Plants of the World Online. *Geranium sanguineum* L.. https://powo.science.kew.org/taxon/urn:lsid:ipni.org:names:322494-2.

[12] Royal Botanic Gardens, Kew Plants of the World Online. *Geranium macrorrhizum* L.. https://powo.science.kew.org/taxon/urn:lsid:ipni.org:names:373281-1.

[13] Benzel I.L., Hordiienko O.I., Hroshovyi T.A., Benzel L.V., Pokryshko O.V. (2018). Obtaining of *Geranium sanguineum* Phytoextracts and Study of Their Anti-Microbial Properties. Int. J. Green Pharm..

[14] Abarova S., Alexova R., Dragomanova S., Solak A., Fagone P., Mangano K., Petralia M.C., Nicoletti F., Kalfin R., Tancheva L. (2024). Emerging Therapeutic Potential of Polyphenols from *Geranium sanguineum* L. in Viral Infections, Including SARS-CoV-2. Biomolecules.

[15] Zeljković S.C., Siljak-Yakovlev S., Tan K., Maksimović M. (2020). Chemical Composition and Antioxidant Activity of *Geranium macrorrhizum* in Relation to Ploidy Level and Environmental Conditions. Plant Syst. Evol..

[16] Ilić M., Samardžić S., Kotur-Stevuljević J., Ušjak D., Milenković M., Kovačević N., Drobac M. (2021). Polyphenol Rich Extracts of *Geranium* L. Species as Potential Natural Antioxidant and Antimicrobial Agents. Eur. Rev. Med. Pharmacol. Sci..

[17] Radulović N.S., Stojković M.B., Mitić S.S., Randjelović P.J., Ilić I.R., Stojanović N.M., Stojanović-Radić Z.Z. (2012). Exploitation of the Antioxidant Potential of *Geranium macrorrhizum* (Geraniaceae): Hepatoprotective and Antimicrobial Activities. Nat. Prod. Commun..

[18] Sharopov F., Ahmed M., Satyal P., Setzer W.N., Wink M. (2017). Antioxidant Activity and Cytotoxicity of Methanol Extracts of *Geranium Macrorrhizum* and Chemical Composition of Its Essential Oil. J. Med. Act. Plants.

[19] Bejenaru C., Segneanu A.-E., Biţă A., Bejenaru L.E., Hovaneţ M.-V., Ciocîlteu M.V., Tîrnă A.C., Blendea A., Mogoşanu G.D. (2025). Polyphenols Investigation and In Vitro Antioxidant Capacity of Romanian Wild-Grown *Geranium* Spp. (Geraniaceae). Plants.

[20] Bas O., Kandilli A.T., Çömlekcioğlu N. (2025). A Review on the Phytochemical Properties, Antimicrobial Activity, and Medicinal Potential of the *Geranium* Genus. Int. J. Chem. Technol..

[21] Ilić M., Samardžić S., Kojić V., Jakimov D., Marčetić M., Drobac M. (2026). Phenolic Profiles and Cytotoxic Activities of Methanolic Extracts From Eight *Geranium* L. Species. Chem. Biodivers..

[22] Pantev A., Ivancheva S., Staneva L., Serkedjieva J. (2006). Biologically Active Constituents of a Polyphenol Extract from *Geranium sanguineum* L. with Anti-Influenza Activity. Z. Naturforsch. C.

[23] Sokmen M., Angelova M., Krumova E., Pashova S., Ivancheva S., Sokmen A., Serkedjieva J. (2005). In Vitro Antioxidant Activity of Polyphenol Extracts with Antiviral Properties from *Geranium sanguineum* L.. Life Sci..

[24] Sabotinova D., Boycheva P., Ivanova N., Andonova V., Georgiev V., Zhelev I. (2026). Comparative Phytochemical Analysis of the Aerial Parts of *Pelargonium radula* and *Geranium macrorrhizum* Cultivated in Bulgaria Using GC-MS and HPLC. Pharmaceuticals.

[25] Camarda L., Budriesi R., Corazza I., Frosini M., Marzetti C., Mattioli L.B. (2026). Antioxidant and Health-Related Effects of Tannins: From Agri-Food By-Products to Human and Animal Health. Antioxidants.

[26] Cosme F., Aires A., Pinto T., Oliveira I., Vilela A., Gonçalves B. (2025). A Comprehensive Review of Bioactive Tannins in Foods and Beverages: Functional Properties, Health Benefits, and Sensory Qualities. Molecules.

[27] Kremer D., Košir I.J., Šimunić M., Srečec S., Jurišić Grubešić R. (2023). Phenolic Compounds in *Geranium dalmaticum* (Beck) Rech. f. and *G. macrorrhizum* L. (Geraniaceae) Growing in Croatia. Glas. Futur..

[28] Leucuta S., Vlase L., Gocan S., Radu L., Fodorea C. (2005). Determination of Phenolic Compounds from *Geranium sanguineum* by HPLC. J. Liq. Chromatogr. Relat. Technol..

[29] Mittal M., Siddiqui M.R., Tran K., Reddy S.P., Malik A.B. (2014). Reactive Oxygen Species in Inflammation and Tissue Injury. Antioxid. Redox Signal..

[30] Chapple I.L.C. (1997). Reactive Oxygen Species and Antioxidants in Inflammatory Diseases. J. Clin. Periodontol..

[31] Miguel M.G. (2010). Antioxidant and Anti-Inflammatory Activities of Essential Oils: A Short Review. Molecules.

[32] Nikolova M., Tsvetkova R., Ivancheva S. (2010). Evaluation of Antioxidant Activity in Some Geraniacean Species. Bot. Serbica.

[33] Gulcin İ., Alwasel S.H. (2022). Metal Ions, Metal Chelators and Metal Chelating Assay as Antioxidant Method. Processes.

[34] Mladěnka P., Macáková K., Filipský T., Zatloukalová L., Jahodář L., Bovicelli P., Silvestri I.P., Hrdina R., Saso L. (2011). In Vitro Analysis of Iron Chelating Activity of Flavonoids. J. Inorg. Biochem..

[35] Olennikov D.N., Kashchenko N.I., Chirikova N.K. (2014). A Novel HPLC-Assisted Method for Investigation of the Fe^2+^-Chelating Activity of Flavonoids and Plant Extracts. Molecules.

[36] Ben Jemia M., Aidi Wannes W., Ouchikh O., Bruno M., Kchouk M.E. (2013). Antioxidant Activity of Tunisian *Geranium robertianum* L. (Geraniaceae). Nat. Prod. Res..

[37] Arnø A., Sarmiento V., Elvebø O., Araujo P. (2025). Critical Evaluation and Validation of a High-Throughput Microplate-Based Cupric Reducing Antioxidant Capacity Method for the Analysis of Fish Feed Ingredients. Antioxidants.

[38] Shahidi F., Zhong Y. (2015). Measurement of Antioxidant Activity. J. Funct. Foods.

[39] Halliwell B. (2012). Free Radicals and Antioxidants: Updating a Personal View. Nutr. Rev..

[40] Xiang Z., Guan H., Zhao X., Xie Q., Xie Z., Cai F., Dang R., Li M., Wang C. (2024). Dietary Gallic Acid as an Antioxidant: A Review of Its Food Industry Applications, Health Benefits, Bioavailability, Nano-Delivery Systems, and Drug Interactions. Food Res. Int..

[41] Choi S.-S., Park H.-R., Lee K.-A. (2021). A Comparative Study of Rutin and Rutin Glycoside: Antioxidant Activity, Anti-Inflammatory Effect, Effect on Platelet Aggregation and Blood Coagulation. Antioxidants.

[42] Wang L., Pan X., Jiang L., Chu Y., Gao S., Jiang X., Zhang Y., Chen Y., Luo S., Peng C. (2022). The Biological Activity Mechanism of Chlorogenic Acid and Its Applications in Food Industry: A Review. Front. Nutr..

[43] Kaul A., Short W.D., Wang X., Keswani S.G. (2021). Hyaluronidases in Human Diseases. Int. J. Mol. Sci..

[44] Sklirou A.D., Angelopoulou M.T., Argyropoulou A., Chaita E., Boka V.I., Cheimonidi C., Niforou K., Mavrogonatou E., Pratsinis H., Kalpoutzakis E. (2021). Phytochemical Study and In Vitro Screening Focusing on the Anti-Aging Features of Various Plants of the Greek Flora. Antioxidants.

[45] Lee K.-K., Kim J.-H., Cho J.-J., Choi J.-D. (1999). Inhibitory Effects of 150 Plant Extracts on Elastase Activity, and Their Anti-Inflammatory Effects. Int. J. Cosmet. Sci..

[46] Hossain M.R., Ansary T.M., Komine M., Ohtsuki M. (2021). Diversified Stimuli-Induced Inflammatory Pathways Cause Skin Pigmentation. Int. J. Mol. Sci..

[47] Zhu H., Fan M., Gao W. (2021). Identification of Potential Hub Genes Associated with Skin Wound Healing Based on Time Course Bioinformatic Analyses. BMC Surg..

[48] Yao Y., Xu B. (2022). Skin Health Promoting Effects of Natural Polysaccharides and Their Potential Application in the Cosmetic Industry. Polysaccharides.

[49] Celikler Ozer O., Orhan I.E., Çalışkan B., Senol Deniz F.S., Gokbulut A., Gur Maz T., Aysal A., Emerce E., Shekfeh S., Kahraman A. (2021). Exploration of Anti-Tyrosinase Effect of *Geranium glaberrimum* Boiss. & Heldr. with in Silico Approach and Survey of 21 *Geranium* Species. J. Herb. Med..

[50] Elmaidomy A.H., Shady N.H., Abdeljawad K.M., Elzamkan M.B., Helmy H.H., Tarshan E.A., Adly A.N., Hussien Y.H., Sayed N.G., Zayed A. (2022). Antimicrobial Potentials of Natural Products against Multidrug Resistance Pathogens: A Comprehensive Review. RSC Adv..

[51] Pane Y.S. (2024). Effectiveness of Traditional Herbal Extracts Against Multidrug-Resistant Bacteria: A Review. bioRxiv.

[52] Mustafa Y.F., Jebir R.M. (2025). Plant-Derived Extracts and Conventional Drugs: A New Frontier in Antimicrobial Therapy. J. Herbmed Pharmacol..

[53] Jeong J.-Y., Jung I.-G., Yum S.-H., Hwang Y.-J. (2023). In Vitro Synergistic Inhibitory Effects of Plant Extract Combinations on Bacterial Growth of Methicillin-Resistant *Staphylococcus aureus*. Pharmaceuticals.

[54] Alshehri B. (2024). The *Geranium* Genus: A Comprehensive Study on Ethnomedicinal Uses, Phytochemical Compounds, and Pharmacological Importance. Saudi J. Biol. Sci..

[55] Ghai I. (2023). A Barrier to Entry: Examining the Bacterial Outer Membrane and Antibiotic Resistance. Appl. Sci..

[56] Matheus G.G., Chamoun M.N., Khosrotehrani K., Sivakumaran Y., Wells T.J. (2025). Understanding the Pathophysiology of *Pseudomonas aeruginosa* Colonization as a Guide for Future Treatment for Chronic Leg Ulcers. Burn. Trauma.

[57] Yamberla I., Pupiales C., Chiliquinga A.J., Sulca-Villamarín T., Plasencia A., Cabrera Aulestia F., Díaz R.F., Caicedo A., Barba P.M. (2025). *Pseudomonas aeruginosa* Pathogenicity and Its Interaction with Other Microorganisms During the Skin Wound Healing Process. Int. J. Mol. Sci..

[58] Boutzoukas A., Doi Y. (2025). The Global Epidemiology of Carbapenem-Resistant *Acinetobacter baumannii*. JAC-Antimicrob. Resist..

[59] Bigos M., Wasiela M., Kalemba D., Sienkiewicz M. (2012). Antimicrobial Activity of *Geranium* Oil against Clinical Strains of Staphylococcus Aureus. Molecules.

[60] Chen K., Peng C., Chi F., Yu C., Yang Q., Li Z. (2022). Antibacterial and Antibiofilm Activities of Chlorogenic Acid Against *Yersinia enterocolitica*. Front. Microbiol..

[61] Miklasińska-Majdanik M., Kępa M., Wąsik T.J., Zapletal-Pudełko K., Klim M., Wojtyczka R.D. (2023). The Direction of the Antibacterial Effect of Rutin Hydrate and Amikacin. Antibiotics.

[62] Nguyen T.L.A., Bhattacharya D. (2022). Antimicrobial Activity of Quercetin: An Approach to Its Mechanistic Principle. Molecules.

[63] Bangar S.P., Chaudhary V., Sharma N., Bansal V., Ozogul F., Lorenzo J.M. (2023). Kaempferol: A Flavonoid with Wider Biological Activities and Its Applications. Crit. Rev. Food Sci. Nutr..

[64] Delisle-Houde M., Blais M., Tweddell R.J., Rioux D. (2021). Antibacterial Activity of Geraniin from Sugar Maple Leaves: An Ultrastructural Study with the Phytopathogen Xanthomonas Campestris Pv. Vitians. J. Plant Pathol. Int. J. Ital. Phytopathol. Soc..

[65] Leite M.C.A., de Brito Bezerra A.P., de Sousa J.P., de Oliveira Lima E. (2015). Investigating the Antifungal Activity and Mechanism(s) of Geraniol against *Candida albicans* Strains. Med. Mycol..

[66] Jain K., Wadhwa K., Malik M., Haque S., Prieto M.A., Kaur H. (2026). Genomic Insights of *Candida krusei*, an Emerging Fungal Pathogen With Intrinsic Antifungal Resistance. Open Forum Infect. Dis..

[67] Zhang J., Xu S., Tian J., Zhou Z. (2025). Investigating in Vitro Antifungal Mechanisms of Geraniol against *Candida albicans*. Arch. Microbiol..

[68] Vasconcelos P.G.S., Lee K.M., Abuna G.F., Costa E.M.M.B., Murata R.M. (2024). Monoterpene Antifungal Activities: Evaluating Geraniol, Citronellal, and Linalool on *Candida* Biofilm, Host Inflammatory Responses, and Structure–Activity Relationships. Front. Pharmacol..

[69] Singh S., Fatima Z., Ahmad K., Hameed S. (2018). Fungicidal Action of Geraniol against Candida Albicans Is Potentiated by Abrogated CaCdr1p Drug Efflux and Fluconazole Synergism. PLoS ONE.

[70] Mahboubi M., Mahdizadeh E., HeidaryTabar R. (2018). The Anti-Candidal Activity of Pelargonium Graveolens Essential Oils against Clinical Isolates of *Candida albicans*. Infectio.

[71] Singhal M., Shaha S., Katsikogianni M. (2025). Comparative Analysis of Cytotoxicity Assays, from Traditional to Modern Approaches. Cytotoxicity—A Crucial Toxicity Test for In Vitro Experiments.

[72] (2009). Biological Evaluation of Medical Devices—Part 5: Tests for In Vitro Cytotoxicity.

[73] Sergazy S., Vetrova A., Orhan I.E., Senol Deniz F.S., Kahraman A., Zhang J.-Y., Aljofan M. (2022). Antiproliferative and Cytotoxic Activity of Geraniaceae Plant Extracts Against Five Tumor Cell Lines. Futur. Sci. OA.

[74] Agyare C., Lechtenberg M., Deters A., Petereit F., Hensel A. (2011). Ellagitannins from Phyllanthus Muellerianus (Kuntze) Exell.: Geraniin and Furosin Stimulate Cellular Activity, Differentiation and Collagen Synthesis of Human Skin Keratinocytes and Dermal Fibroblasts. Phytomedicine.

[75] Wang P., Peng X., Wei Z.-F., Wei F.-Y., Wang W., Ma W.-D., Yao L.-P., Fu Y.-J., Zu Y.-G. (2015). Geraniin Exerts Cytoprotective Effect against Cellular Oxidative Stress by Upregulation of Nrf2-Mediated Antioxidant Enzyme Expression via PI3K/AKT and ERK1/2 Pathway. Biochim. Biophys. Acta.

[76] Studzińska-Sroka E., Galanty A., Gościniak A., Wieczorek M., Kłaput M., Dudek-Makuch M., Cielecka-Piontek J. (2021). Herbal Infusions as a Valuable Functional Food. Nutrients.

[77] Paczkowska-Walendowska M., Gościniak A., Szymanowska D., Szwajgier D., Baranowska-Wójcik E., Szulc P., Dreczka D., Simon M., Cielecka-Piontek J. (2021). Blackberry Leaves as New Functional Food? Screening Antioxidant, Anti-Inflammatory and Microbiological Activities in Correlation with Phytochemical Analysis. Antioxidants.

[78] Studzińska-Sroka E., Bulicz M., Henkel M., Rosiak N., Paczkowska-Walendowska M., Szwajgier D., Baranowska-Wójcik E., Korybalska K., Cielecka-Piontek J. (2024). Pleiotropic Potential of *Evernia prunastri* Extracts and Their Main Compounds Evernic Acid and Atranorin: In Vitro and In Silico Studies. Molecules.

[79] Chanaj-Kaczmarek J., Osmałek T., Szymańska E., Winnicka K., Karpiński T.M., Dyba M., Bekalarska-Dębek M., Cielecka-Piontek J. (2021). Development and Evaluation of Thermosensitive Hydrogels with Binary Mixture of *Scutellariae baicalensis radix* Extract and Chitosan for Periodontal Diseases Treatment. Int. J. Mol. Sci..

[80] Dinis T.C.P., Madeira V.M.C., Almeida L.M. (1994). Action of Phenolic Derivatives (Acetaminophen, Salicylate, and 5-Aminosalicylate) as Inhibitors of Membrane Lipid Peroxidation and as Peroxyl Radical Scavengers. Arch. Biochem. Biophys..

[81] Santos J.S., Brizola V.R.A., Granato D. (2017). High-Throughput Assay Comparison and Standardization for Metal Chelating Capacity Screening: A Proposal and Application. Food Chem..

[82] Apak R., Güçlü K., Özyürek M., Çelik S.E. (2008). Mechanism of Antioxidant Capacity Assays and the CUPRAC (Cupric Ion Reducing Antioxidant Capacity) Assay. Microchim. Acta.

[83] Studzińska-Sroka E., Dudek-Makuch M., Chanaj-Kaczmarek J., Czepulis N., Korybalska K., Rutkowski R., Łuczak J., Grabowska K., Bylka W., Witowski J. (2018). Anti-Inflammatory Activity and Phytochemical Profile of *Galinsoga parviflora* Cav. Molecules.

[84] Studzińska-Sroka E., Majchrzak-Celińska A., Bańdurska M., Rosiak N., Szwajgier D., Baranowska-Wójcik E., Szymański M., Gruszka W., Cielecka-Piontek J. (2022). Is Caperatic Acid the Only Compound Responsible for Activity of Lichen *Platismatia glauca* within the Nervous System?. Antioxidants.

[85] Bieth J., Spiess B., Wermuth C.G. (1974). The Synthesis and Analytical Use of a Highly Sensitive and Convenient Substrate of Elastase. Biochem. Med..

[86] Lim T.Y., Lim Y.Y., Yule C.M. (2009). Evaluation of Antioxidant, Antibacterial and Anti-Tyrosinase Activities of Four *Macaranga* Species. Food Chem..

