# Supplementary material for: Phytochemical Characterisation and Skin-Relevant In Vitro Biological Activity of Leaf Extracts from Selected Geranium Species
Source: Molecules. 2026 Jul 8;31(14):2406. doi: 10.3390/molecules31142406 (PMC13416177; doi:10.3390/molecules31142406)
Supplement: Supplementary file 1 [file molecules-31-02406-s001.zip › molecules-4340673-supplementary.pdf]

molecules

Supplementary Materials: Phytochemical Characterisation and Skin-Relevant In Vitro Biological Activity of Leaf Extracts from Selected *Geranium* Species

Maciej Książkiewicz [1], Emil Paluch [2], Jarosław Widelski [3], Justyna Stefanowicz-Hajduk [4], Kinga Kochan-Jamrozy [4], Olga Bortkiewicz [2], Krzysztof Kamil Wojtanowski [3], Magdalena Gucwa [4], Judyta Cielecka-Piontek [1] and Elżbieta Studzińska-Sroka [1,*]

[1] Department of Pharmacognosy and Biomaterials, Poznan University of Medical Sciences, 3 Rokietnicka Str., 60-806 Poznan, Poland
[2] Department of Microbiology, Faculty of Medicine, Wroclaw Medical University, 4 T. Chałubinskiego Str., 50-376 Wroclaw, Poland
[3] Department of Pharmacognosy with Medicinal Plants Garden, Lublin Medical University, 20-093 Lublin, Poland
[4] Department of Biology and Pharmaceutical Botany, Medical University of Gdańsk, 107 Al. Gen. J. Hallera Str., 80-416 Gdańsk, Poland

* Correspondence: elastudzinska@ump.edu.pl

Compound_1_GP:

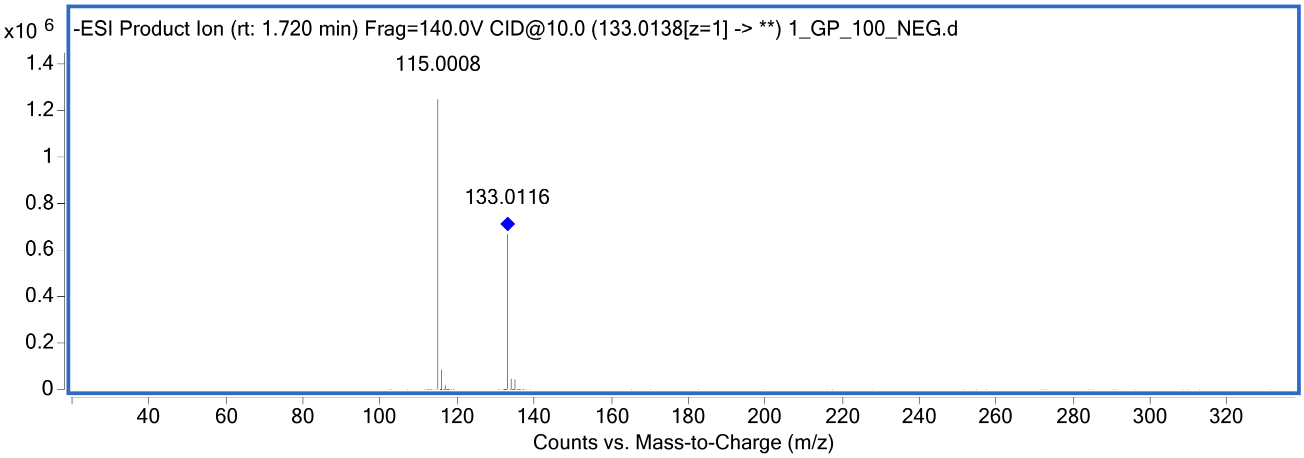

Compound_2_GP:

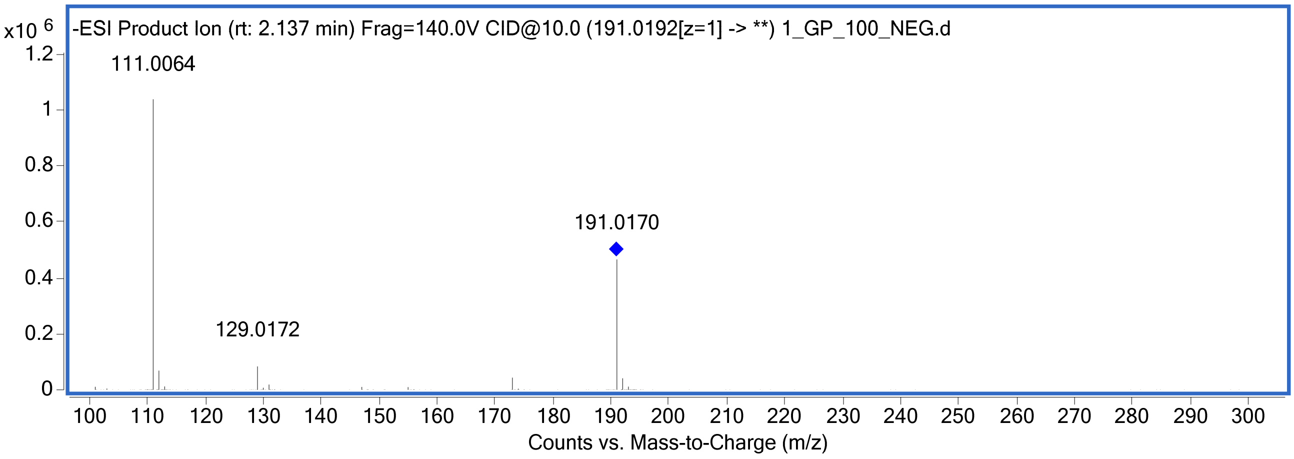

Compound_3_GP:

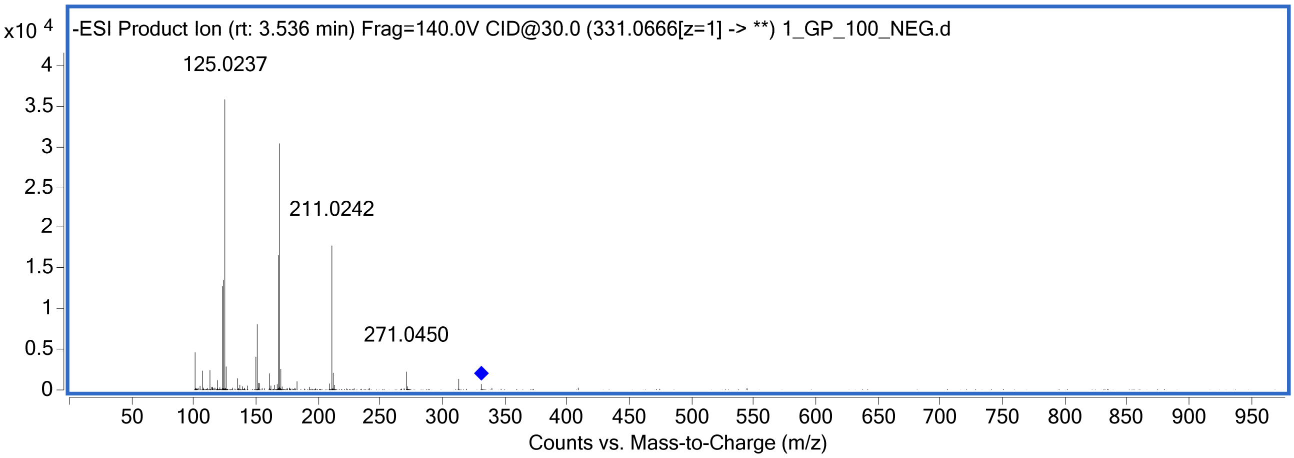

Compound_4_GP:

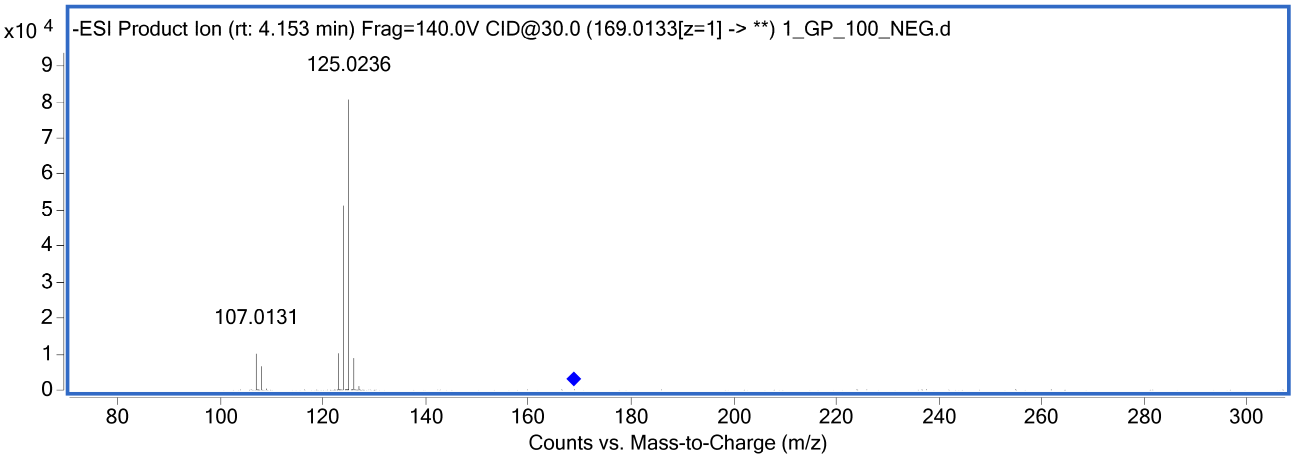

Compound_5_GP:

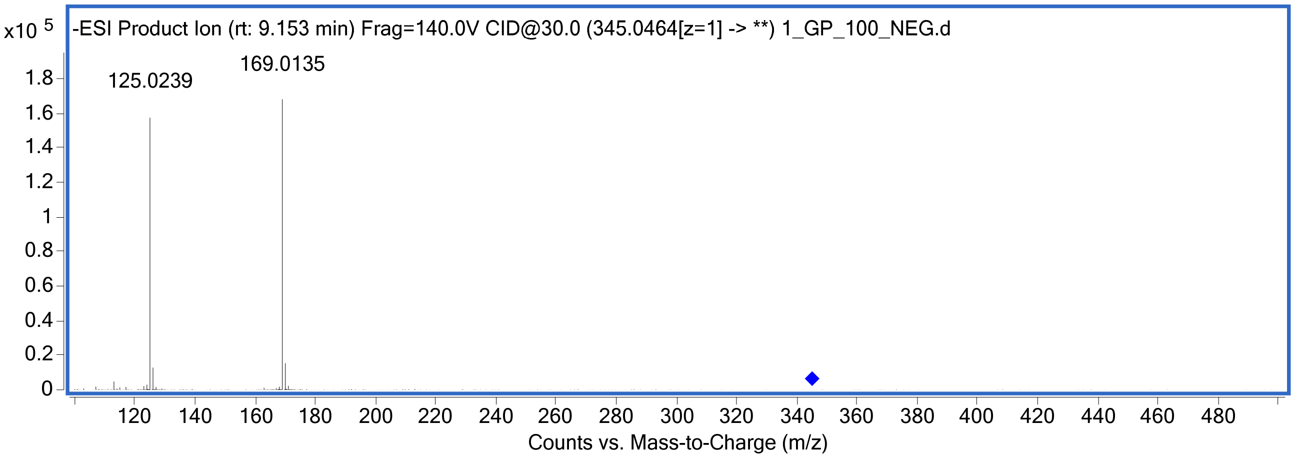

Compound_6_GP:

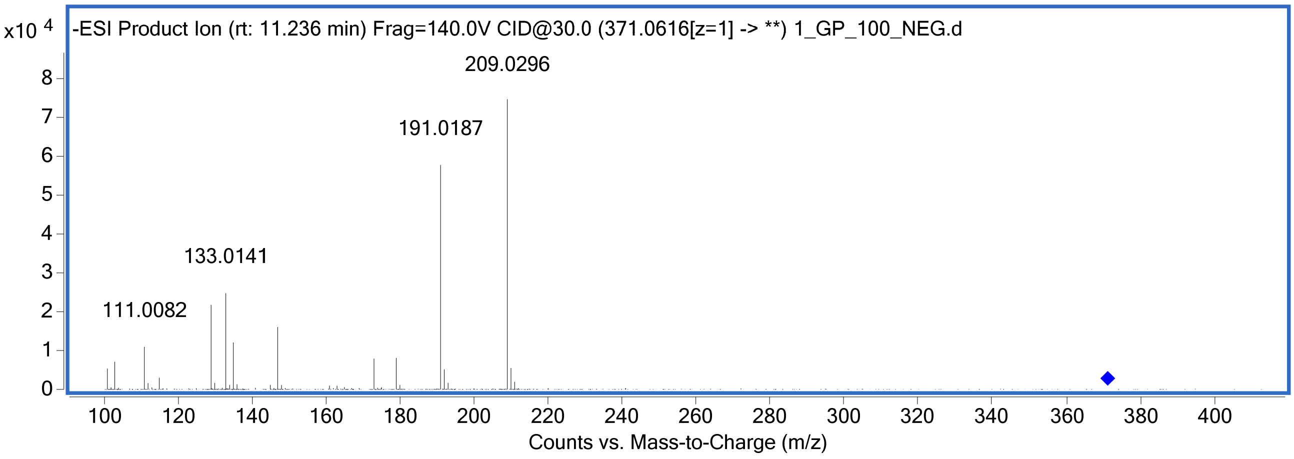

Compound_7_GP:

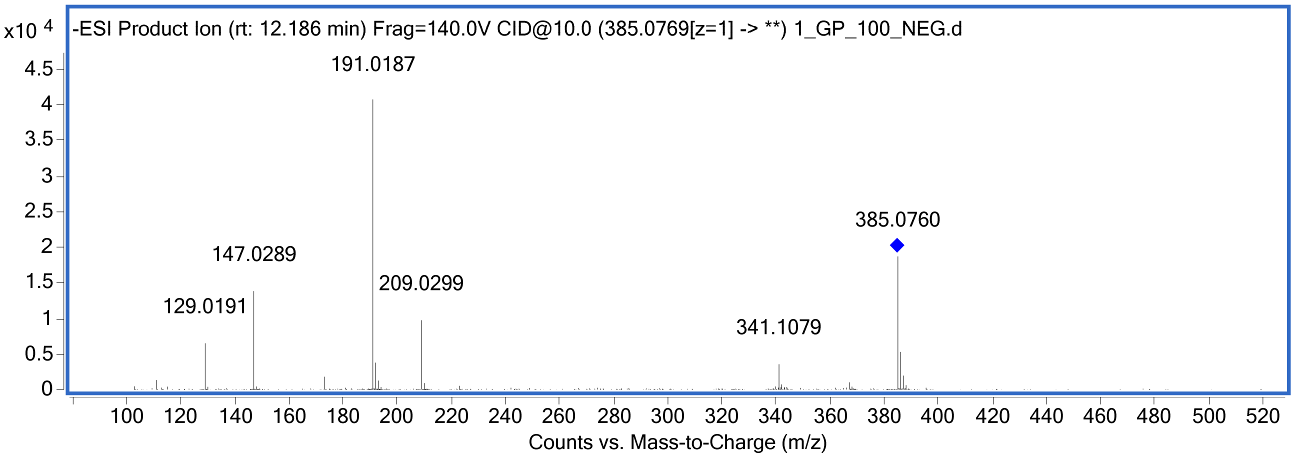

Compound_8_GP:

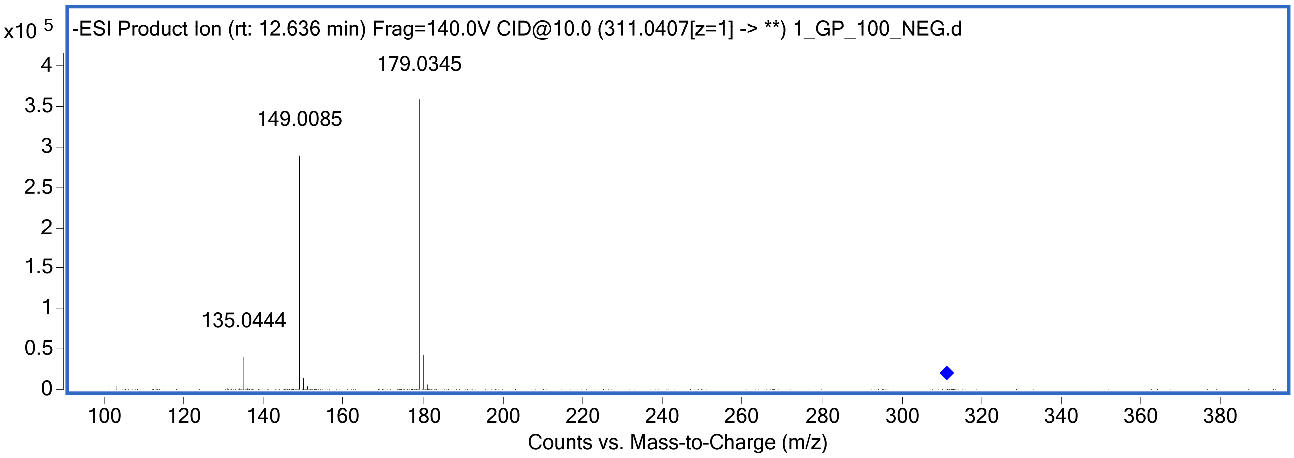

Compound_9_GP:

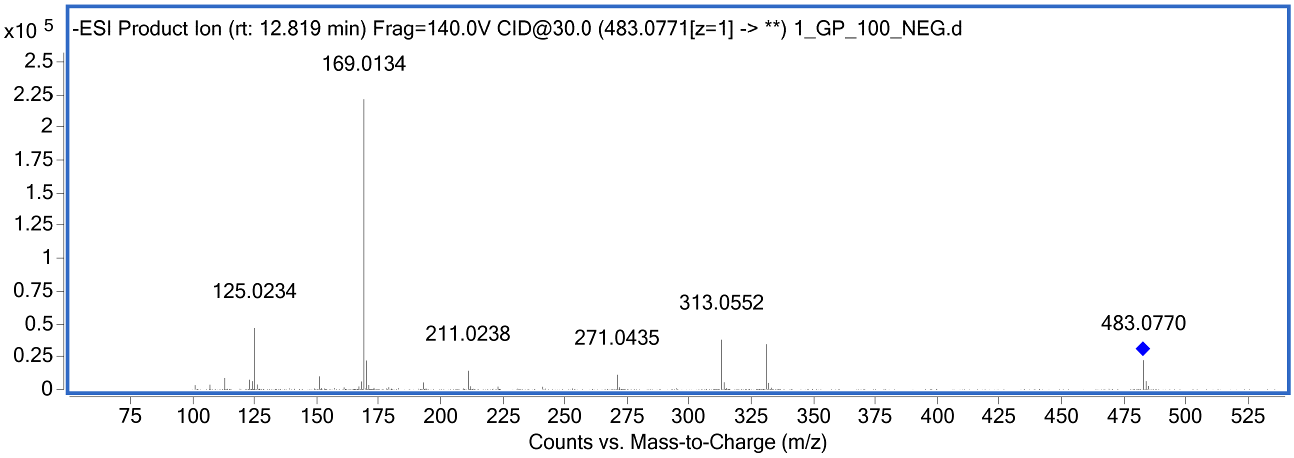

Compound_10_GP:

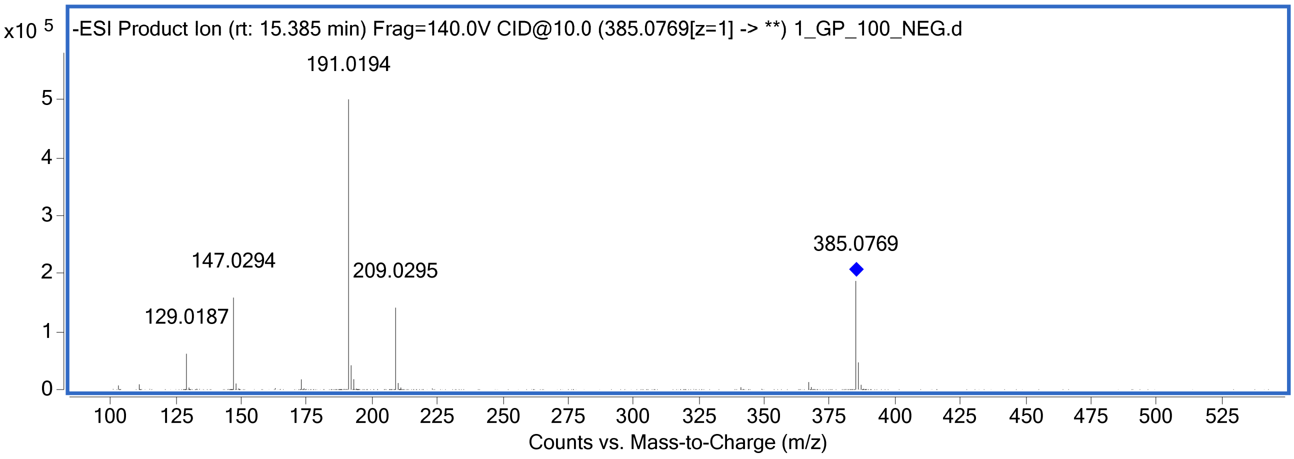

Compound_11_GP:

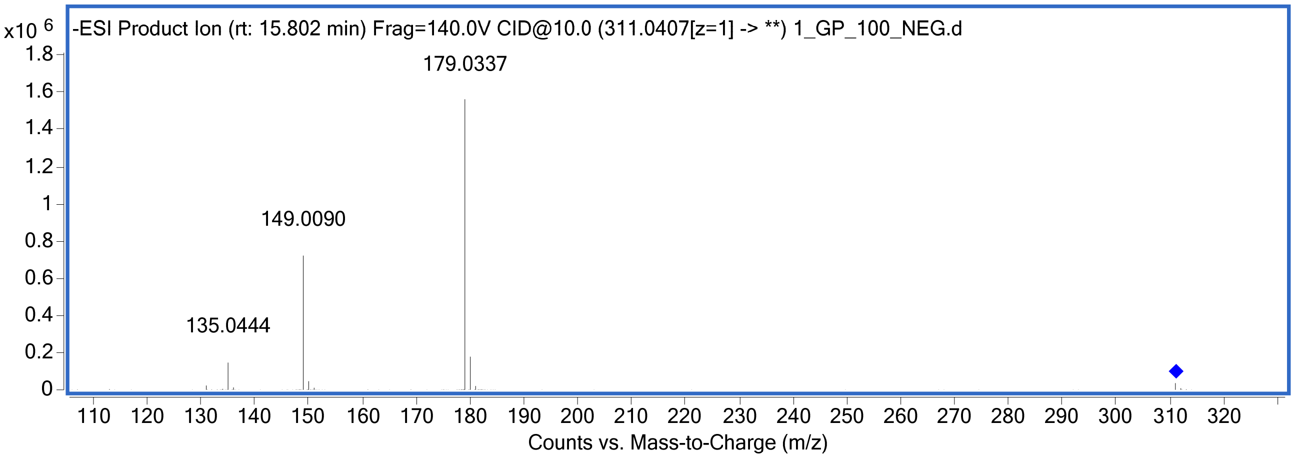

Compound_12_GP:

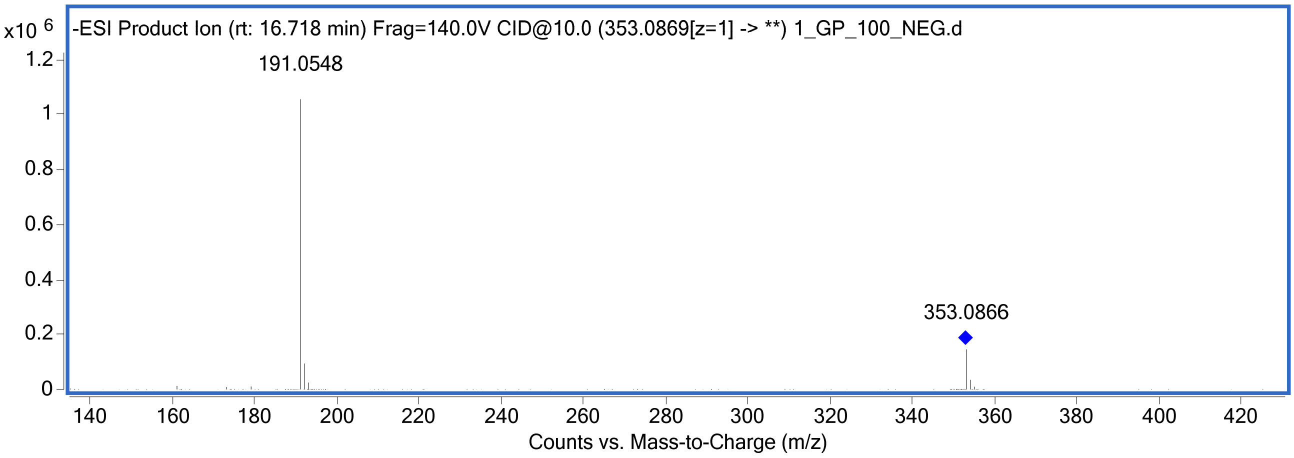

Compound_13_GP:

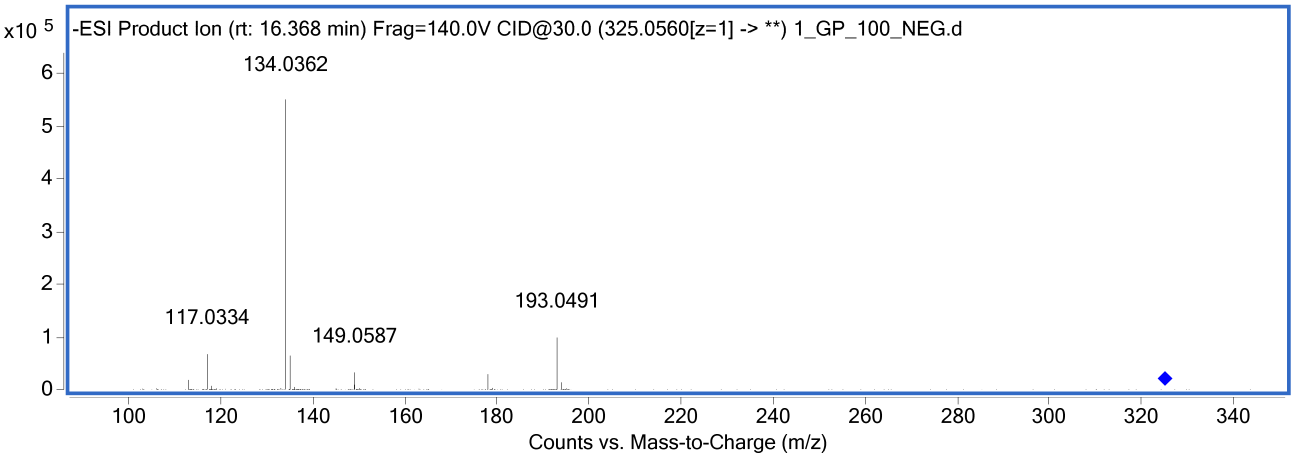

Compound_14_GP:

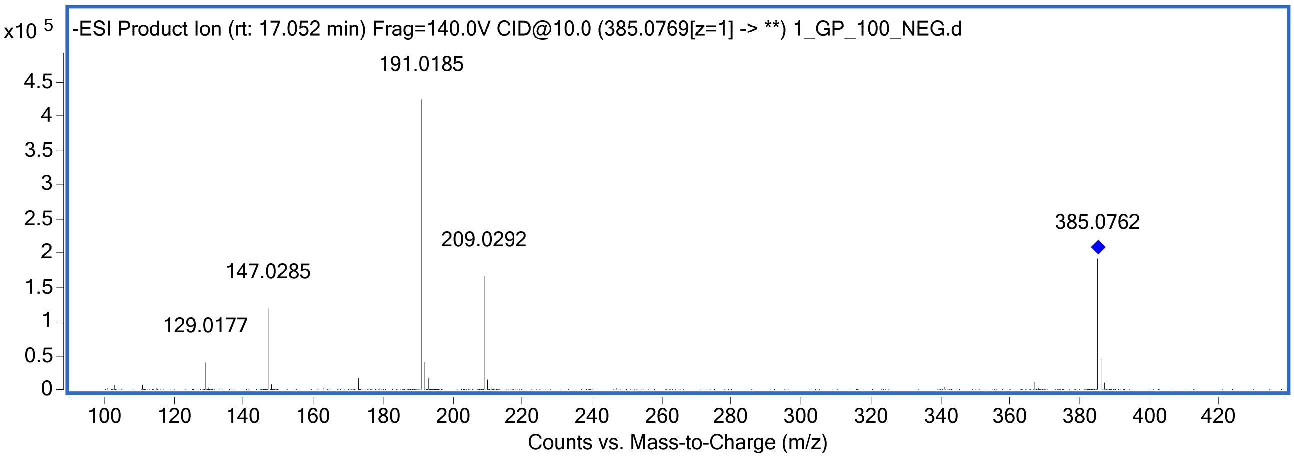

Compound_15_GP:

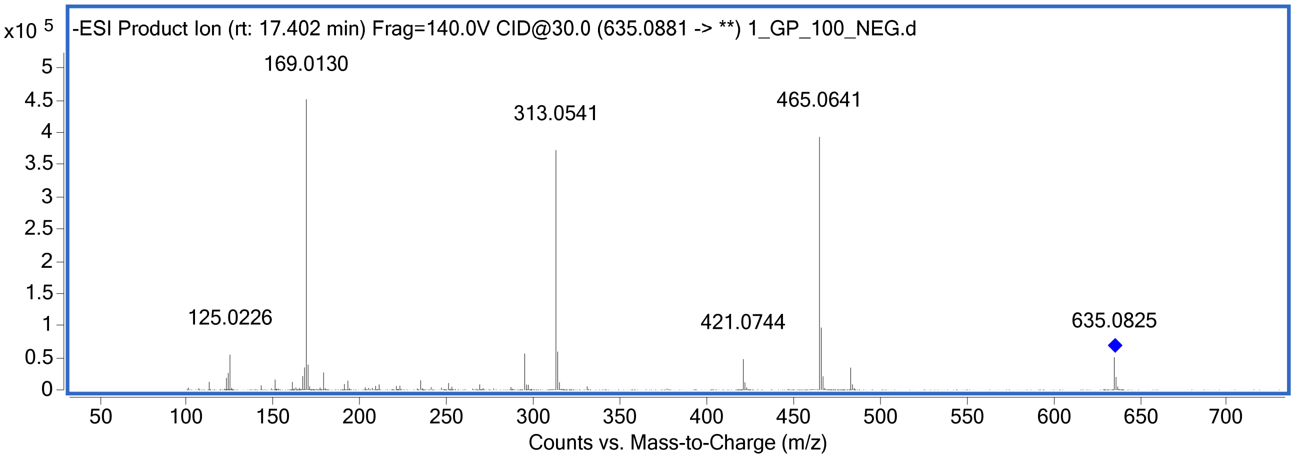

Compound_16_GP:

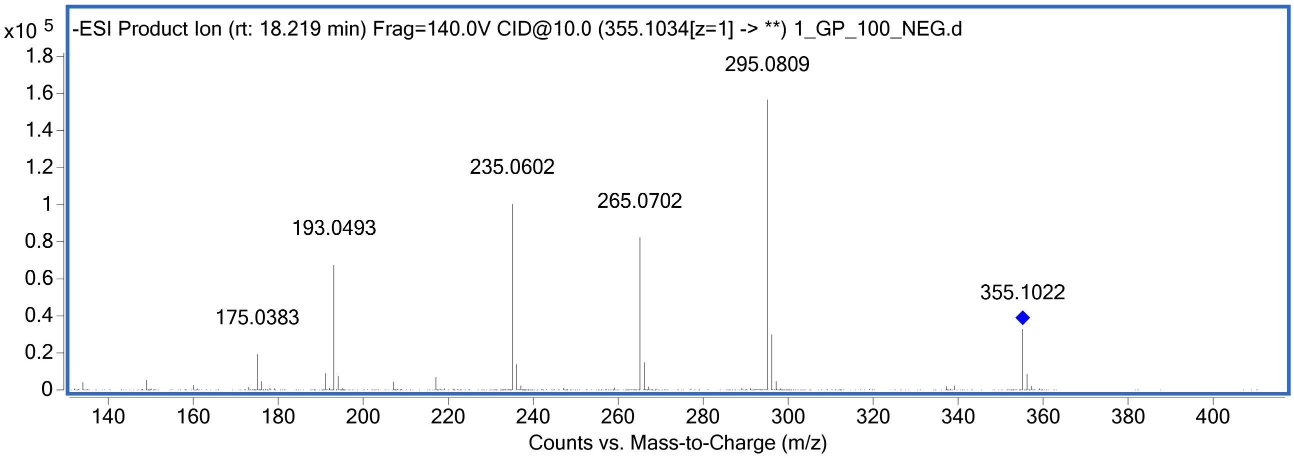

Compound_17_GP:

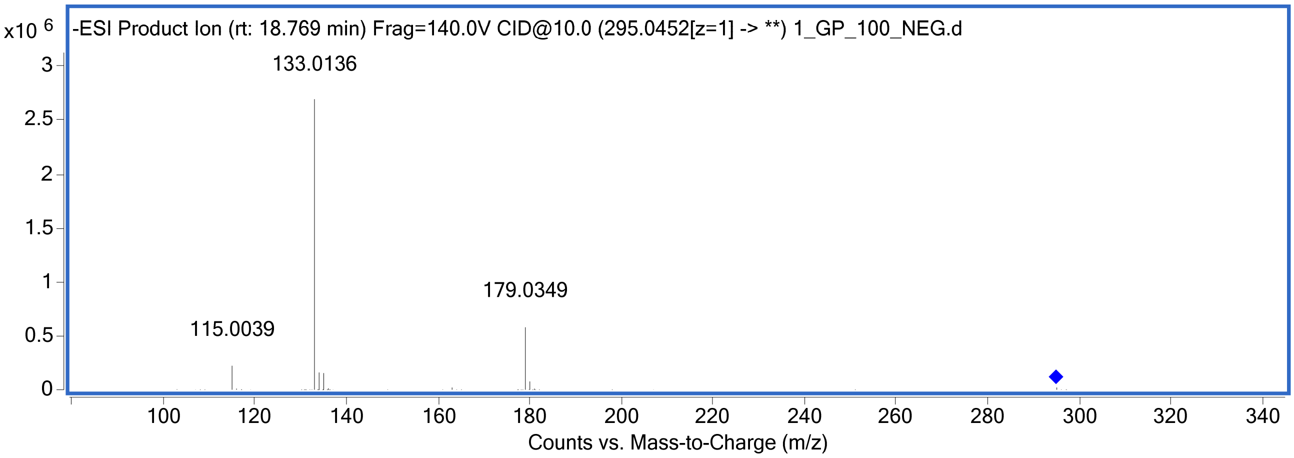

Compound_18_GP:

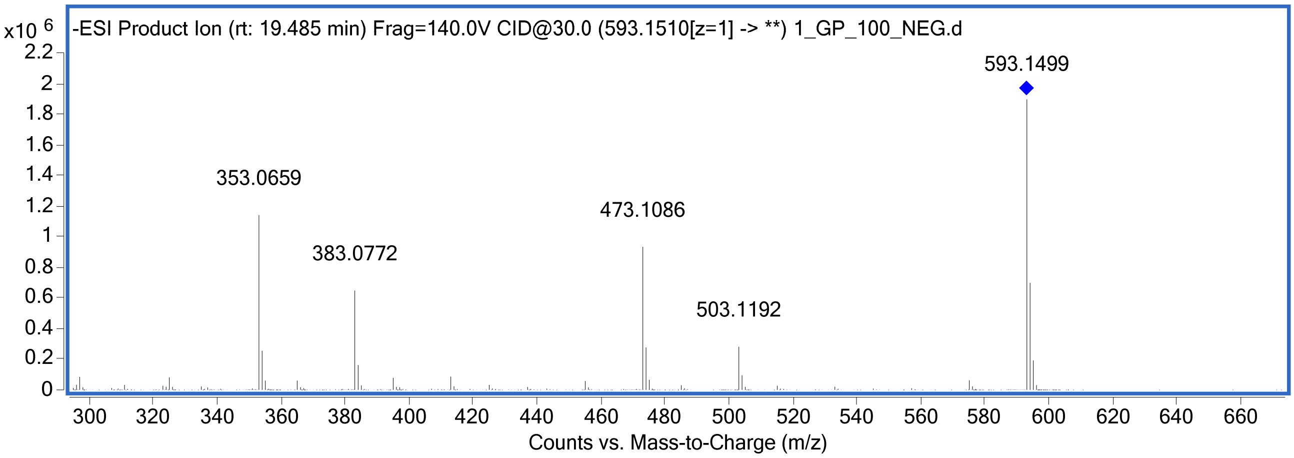

Compound_19_GP:

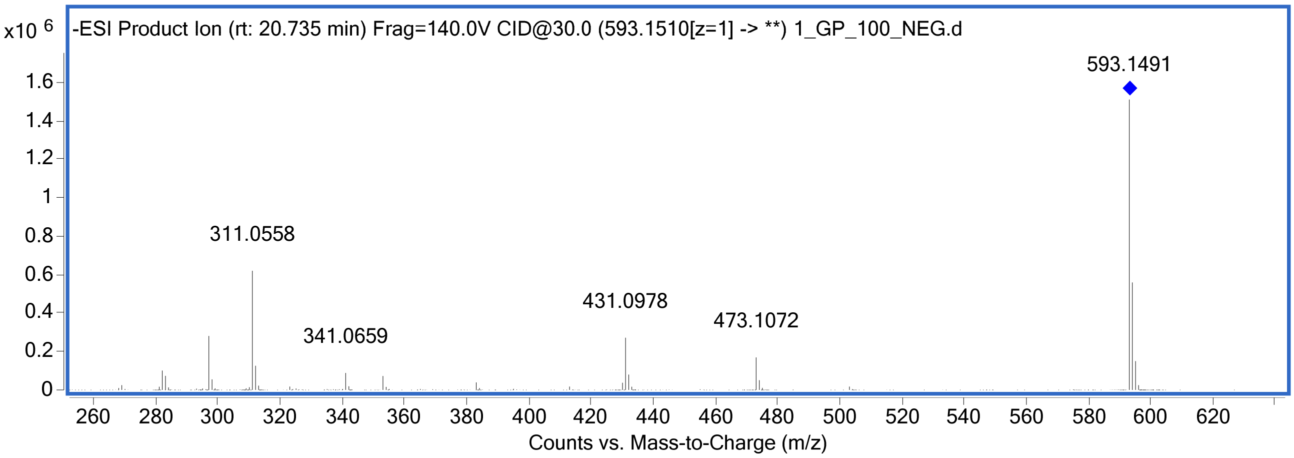

Compound_20_GP:

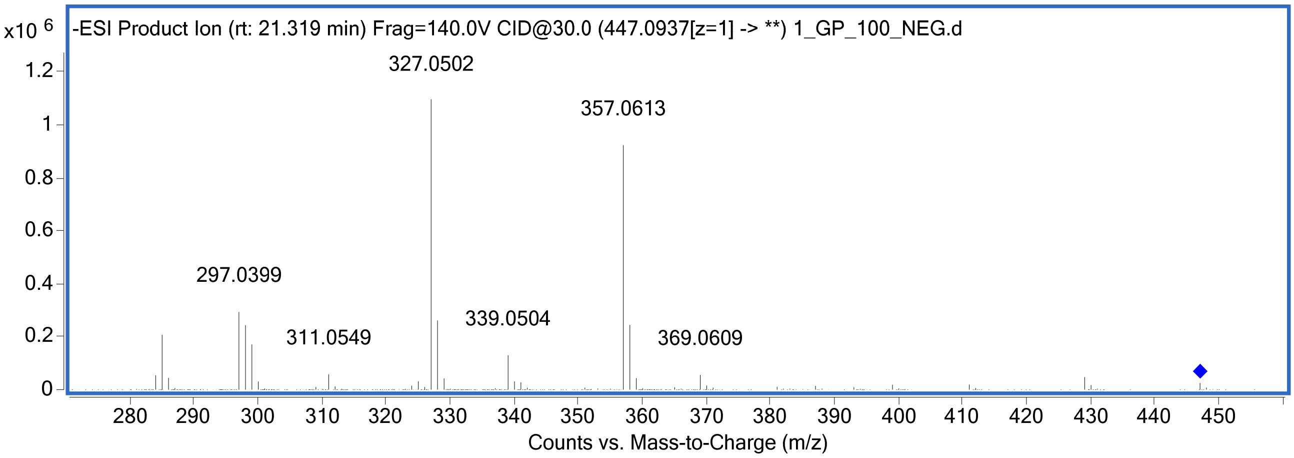

Compound_21_GP:

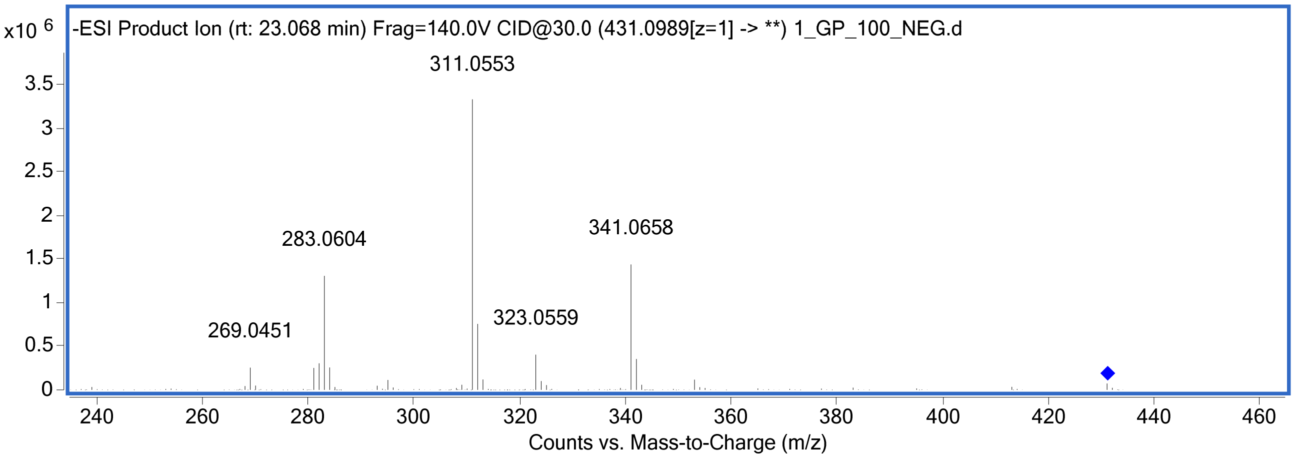

Compound_22_GP:

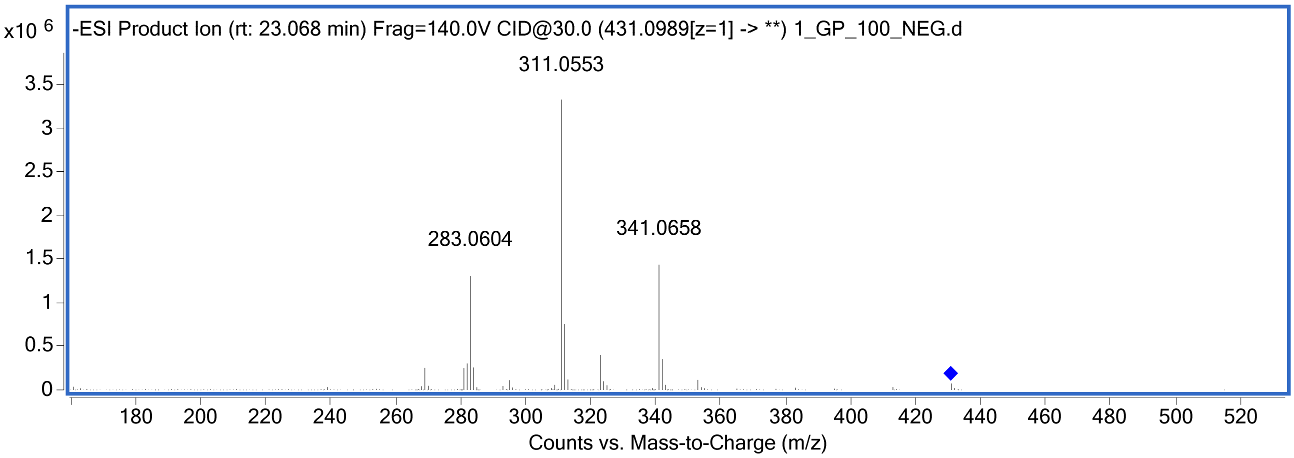

Compound_23_GP:

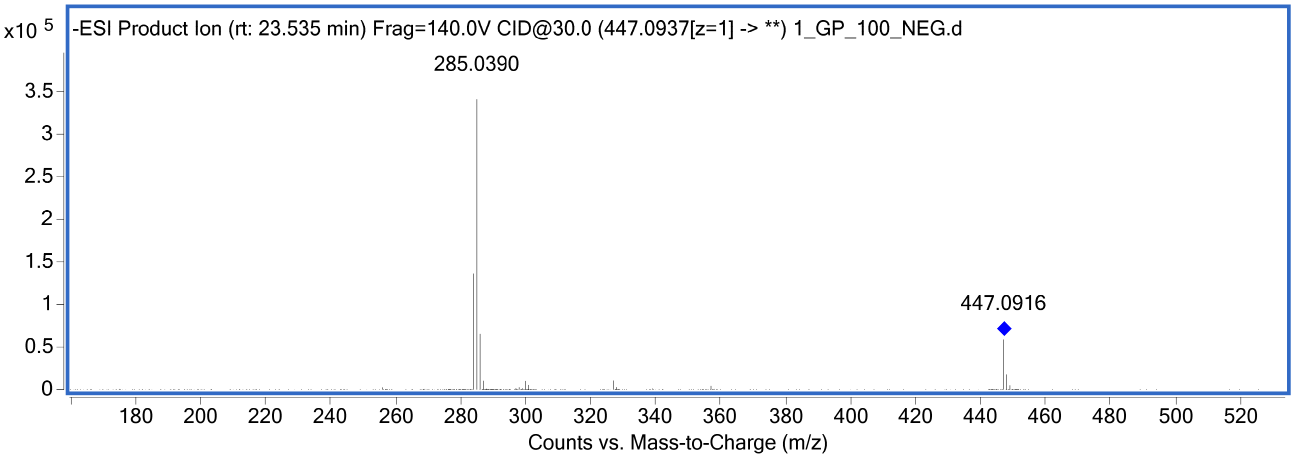

Compoun_24_GP:

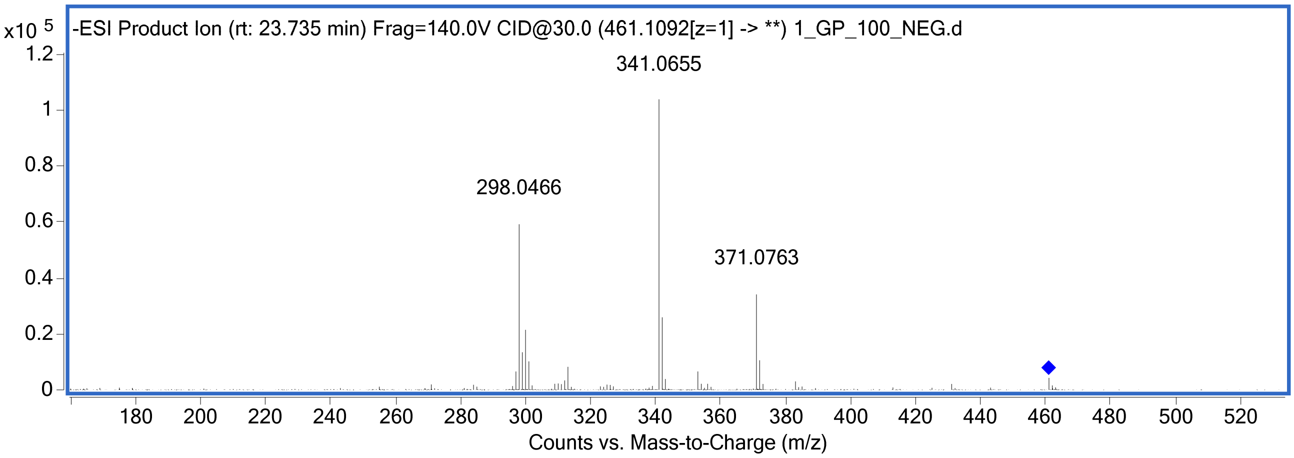

Compound_25_GP:

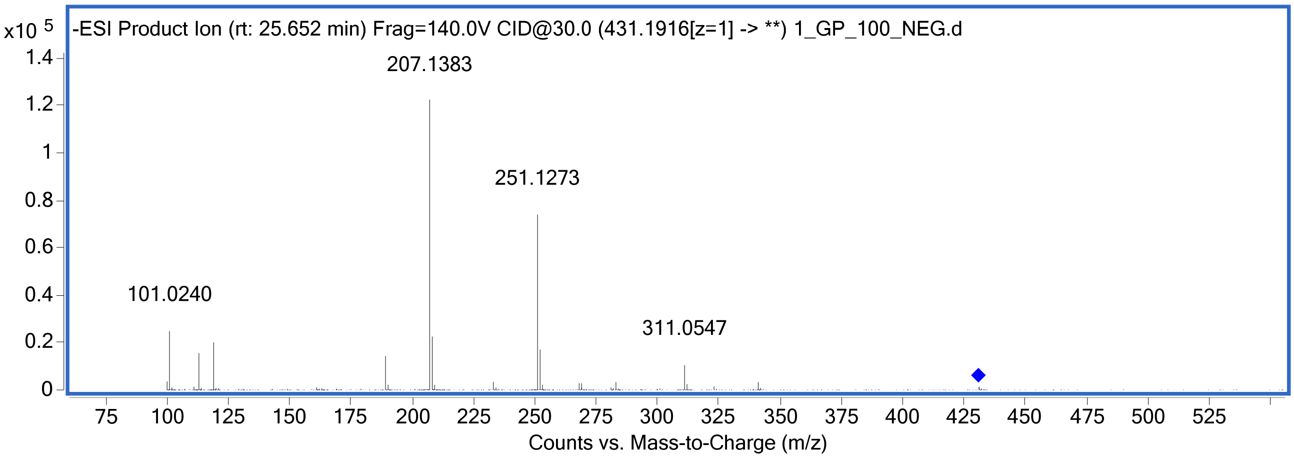

Figure S1. MS/MS product ion spectra obtained by LC–MS/MS analysis of extracts from *Geranium phaeum*.

Table S1. Results of LC-MS/MS analysis of *Geranium phaeum* (GP) extracts.

Number of GP Compound	Tentative assignment	Retention time [min]	Formula	Molecular Ion [m/z] [M-H]$^-$	Error [ppm]	MS/MS fragments [m/z]	MSI level of annotation
1	Malic acid	1.720	$C_4H_6O_5$	133.0138	-3.33	115.0008	2
2	Citric acid	2.137	$C_6H_8O_7$	191.0192	-2.74	129.0172; 111.0064	2
3	Glucogallin	3.536	$C_{13}H_{16}O_{10}$	331.0666	-1.42	271.0450; 211.0242; 169.0131; 151.0026; 125.0237	2
4	Gallic acid	4.153	$C_7H_6O_5$	169.0133	-5.57	125.0236; 107.0131	2
5	Galloyl derivative	9.153	$C_{13}H_{14}O_{11}$	345.0464	0.19	169.0135; 125.0239	2
6	Caffeoylglucaric acid	11.236	$C_{15}H_{16}O_{11}$	371.0616	-1.03	209.0296; 191.0187; 133.0141; 129.0198; 111.0082	2
7	Feruloylglucaric acid isomer 1	12.186	$C_{16}H_{18}O_{11}$	385.0760	-4.23	341.1079; 209.0299; 191.0187; 147.0289; 129.0191	2
8	Caftaric acid	12.636	$C_{13}H_{12}O_9$	311.0407	-0.50	179.0345; 149.0085; 135.0444	2
9	Digallyoylglucose	12.819	$C_{20}H_{20}O_{14}$	483.0770	-2.13	331.0649; 313.0552; 271.0435; 211.0238; 169.0134; 125.0234	2
10	Feruloylglucaric acid isomer 2	15.385	$C_{16}H_{18}O_{11}$	385.0769	-1.90	209.0295; 191.0194; 147.0294;	2

						129.0187	
11	Caffeoyl tartaric acid	15.802	$C_{13}H_{12}O_9$	311.0407	-7.12	179.0337 149.0090; 135.0444	2
12	Feruloyl tartaric acid	16.368	$C_{14}H_{14}O_9$	325.0560	-1.55	193.0491; 149.0587; 134.0362; 117.0334	2
13	Cryptochlorogenic acid	16.718	$C_{16}H_{18}O_9$	353.0869	-2.56	191.0548	2
14	Feruloylglucaric acid isomer 3	17.052	$C_{16}H_{18}O_{11}$	385.0762	-3.72	209.0292; 191.0185; 147.0285; 129.0177	2
15	Trigalloylglucose	17.402	$C_{27}H_{24}O_{18}$	635.0875	-2.34	465.0641; 465.0641; 421.0744; 313.0541; 169.0130; 125.0226	2
16	1-O-feruloyl-beta-D-glucose	18.219	$C_{16}H_{20}O_9$	355.1022	-3.53	295.0809; 265.0702; 235.0602; 193.0493; 175.0383	2
17	Caffeoylmalic acid	18.769	$C_{13}H_{12}O_8$	295.0452	-2.50	179.0349; 133.0136; 115.0039	2
18	Apigenin 6,8-di-C-glucoside	19.485	$C_{27}H_{30}O_{15}$	593.1499	-2.18	503.1192; 473.1083; 383.0772; 353.0659	2
19	Vitexin glucoside	20.735	$C_{27}H_{30}O_{15}$	593.1497	-2.51	473.1072; 431.0978; 341.0659; 311.0558; 297.0401	2
20	Isoorientin	21.319	$C_{21}H_{20}O_{11}$	447.0937	0.93	369.0609; 357.0613; 339.0504; 327.0502; 297.0399;	2

21	Cyanidin 3-O-beta-D-(caffeoyl)-sambubioside	22.152	$C_{35}H_{34}O_{18}$	741.1666	-0.86	431.0968; 311.0555; 193.0487; 115.0028	2
22	Vitexin	23.068	$C_{21}H_{20}O_{10}$	431.0989	1.23	341.0658; 323.0559; 311.0553; 283.0604; 269.0451	2
23	Kaempferol glucoside	23.535	$C_{21}H_{20}O_{11}$	447.0916	-3.76	285.0390	2
24	4-methoxy-kaempferol-3-O-hexoside	23.652	$C_{22}H_{22}O_{11}$	461.1079	-2.24	371.0768; 353.0667; 341.0661; 313.0699; 298.0475	2
25	Isovitexin	25.652	$C_{21}H_{20}O_{10}$	431.1916	-1.55	311.0547; 251.1273; 207.1383; 101.0240	2

Compound_1_GS:

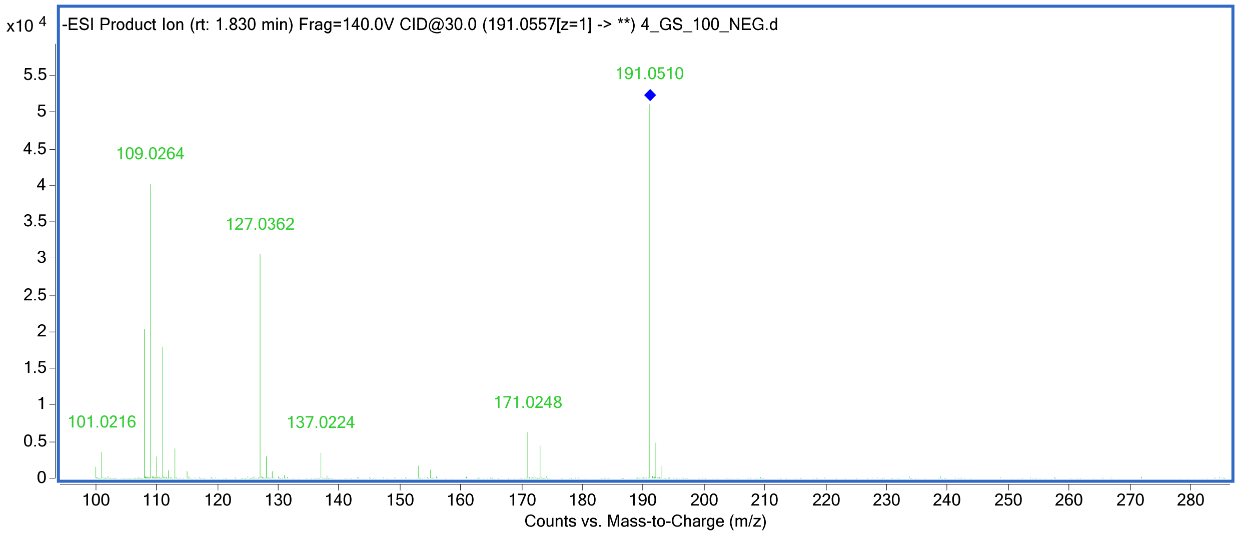

Compound_2_GS:

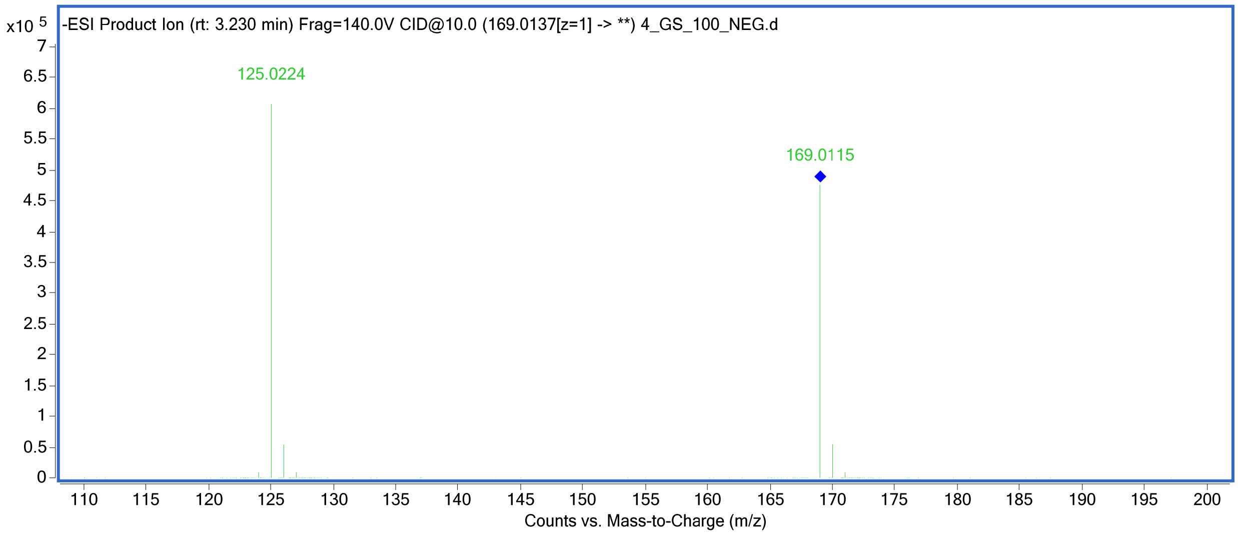

Compound_3_GS:

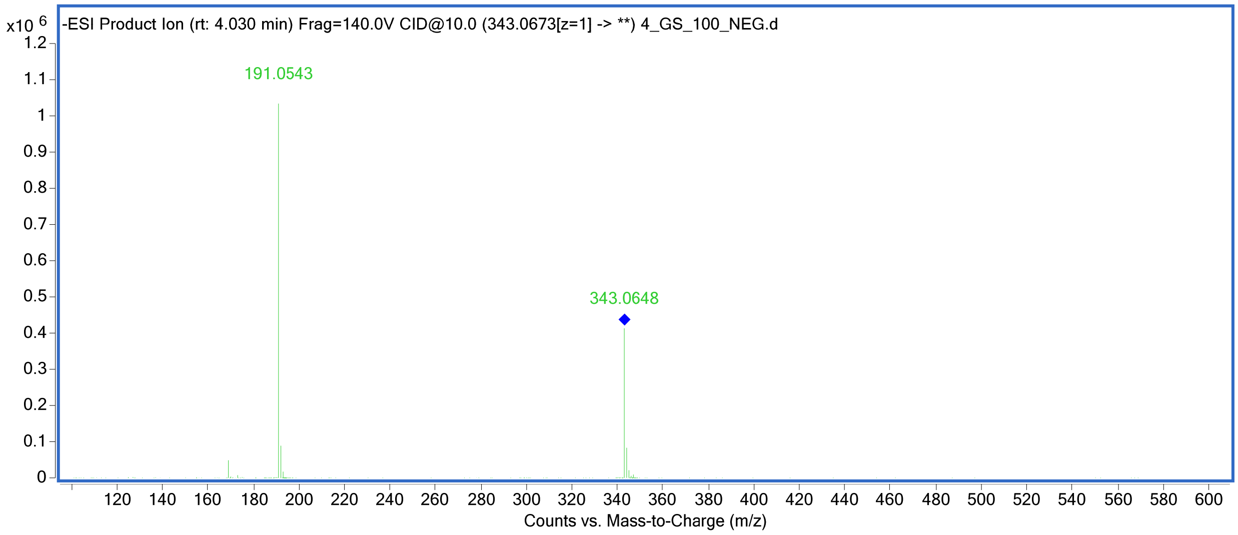

Compound_4_GS:

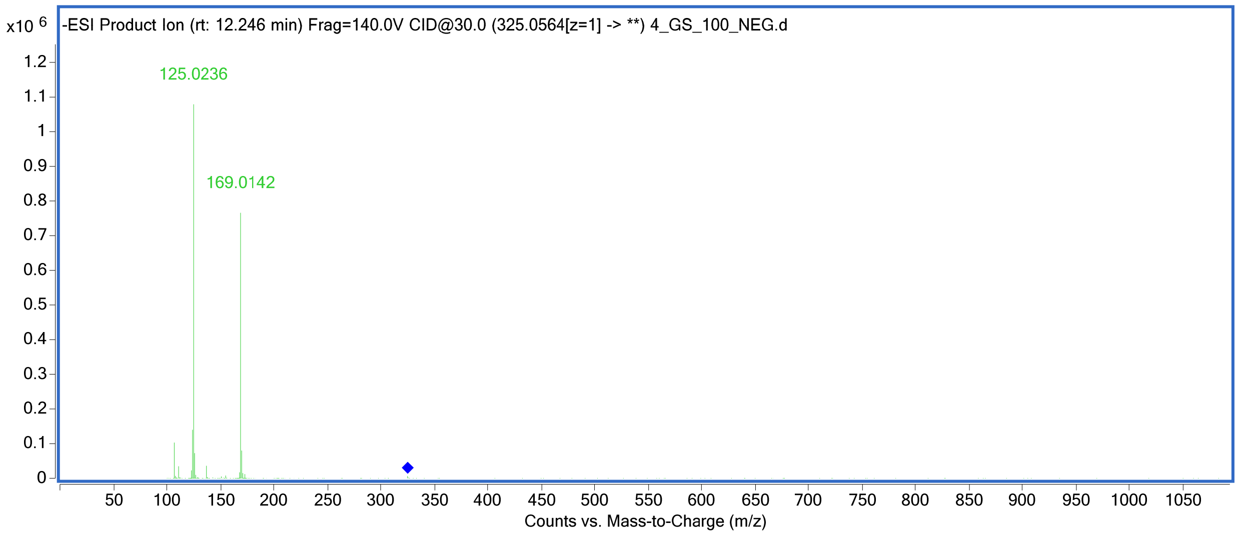

Compound_5_GS:

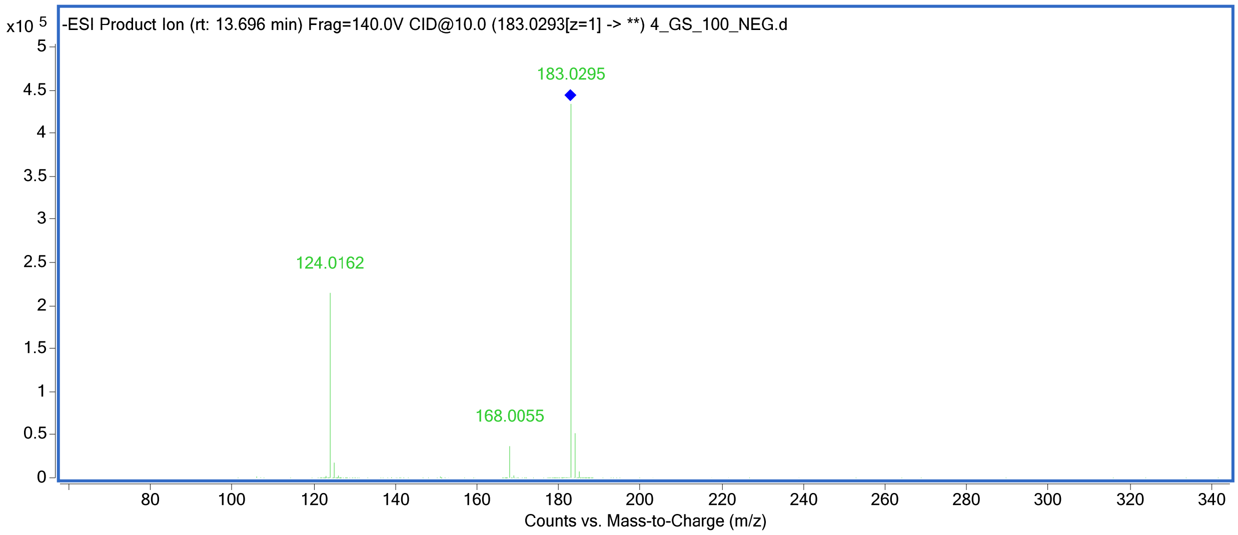

Compound_6_GS:

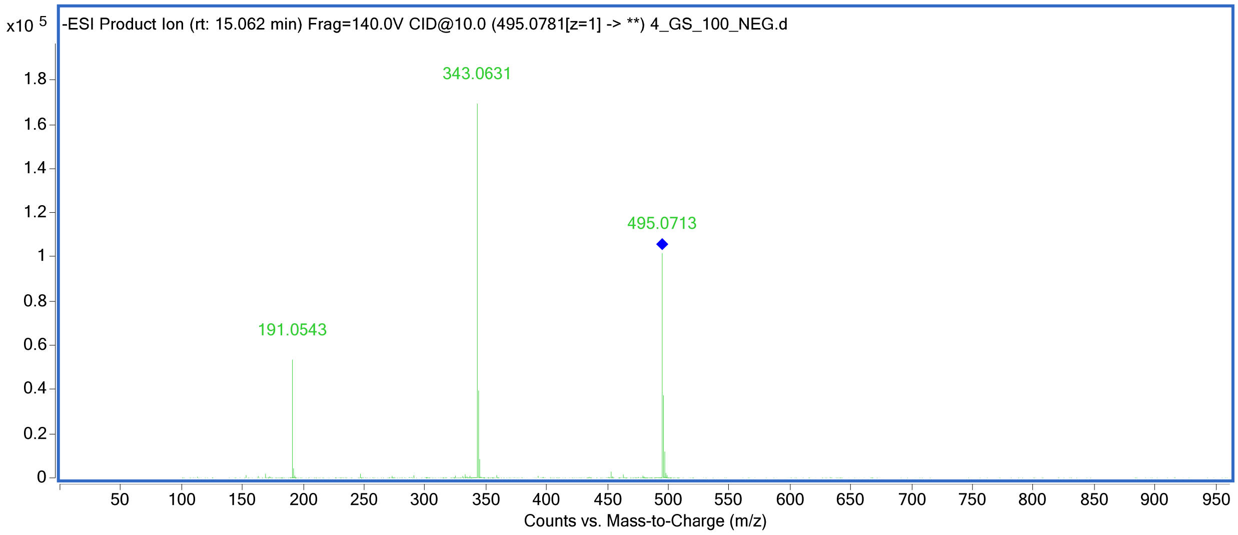

Compound_7_GS:

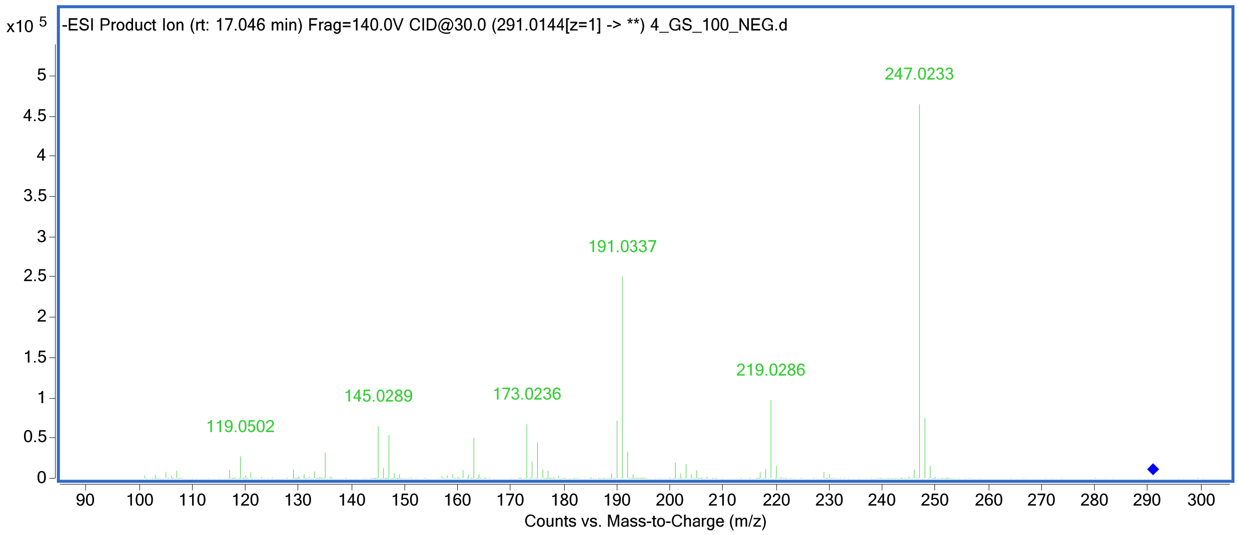

Compound_8_GS:

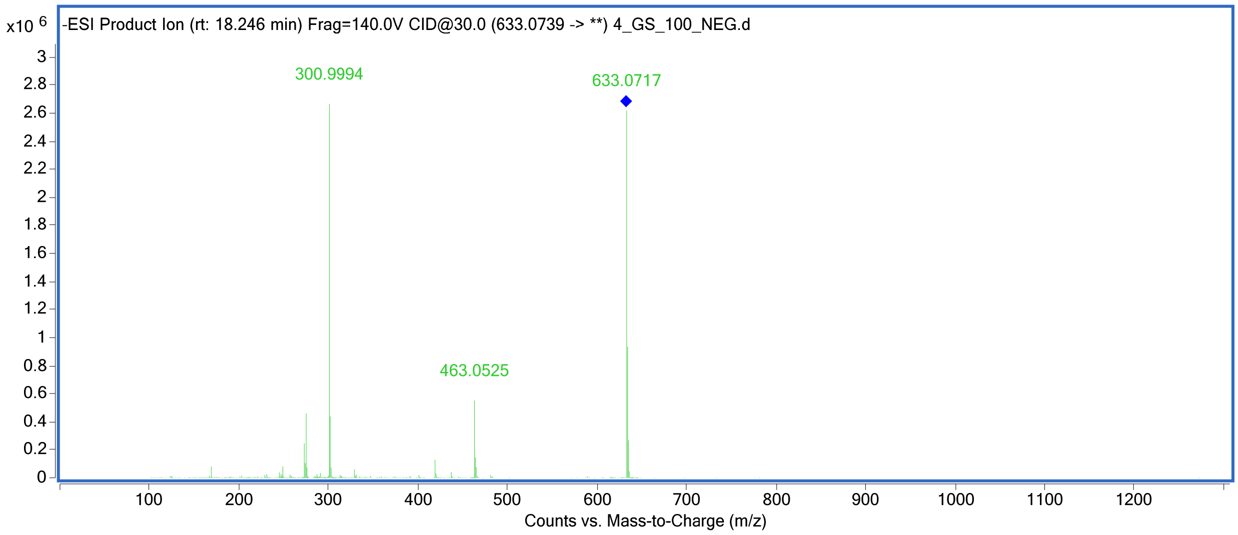

Compound_9_GS:

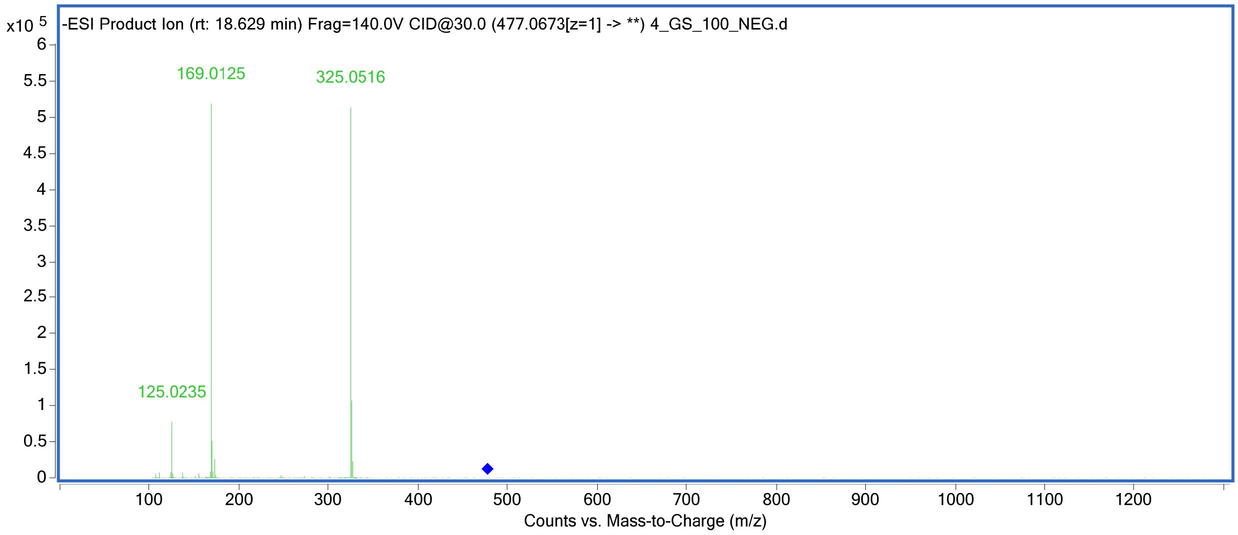

Compound_10_GS:

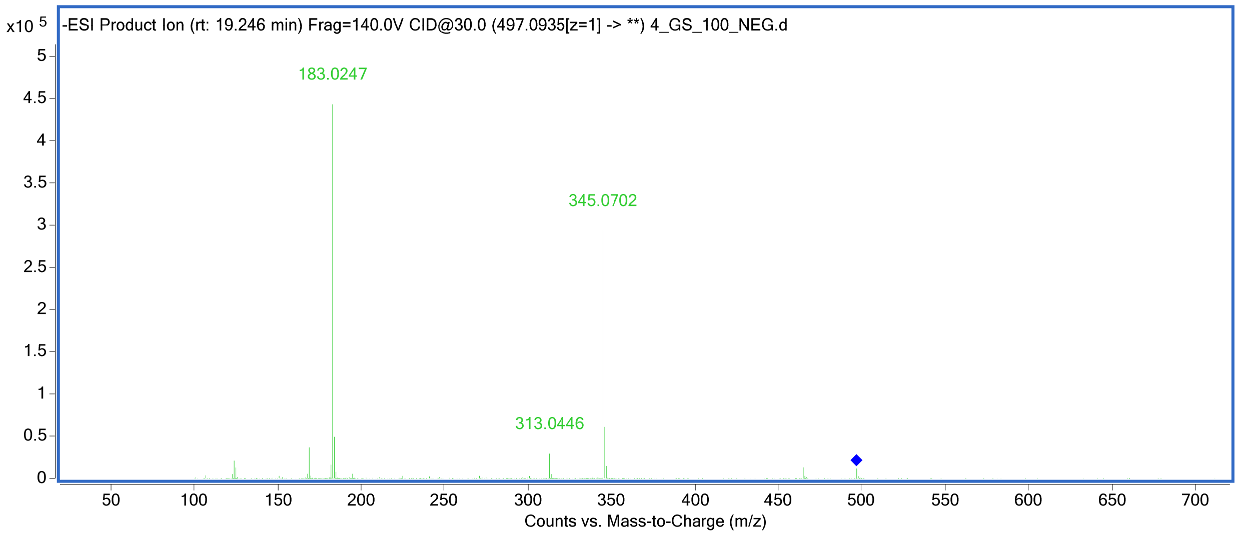

Compound_11_GS:

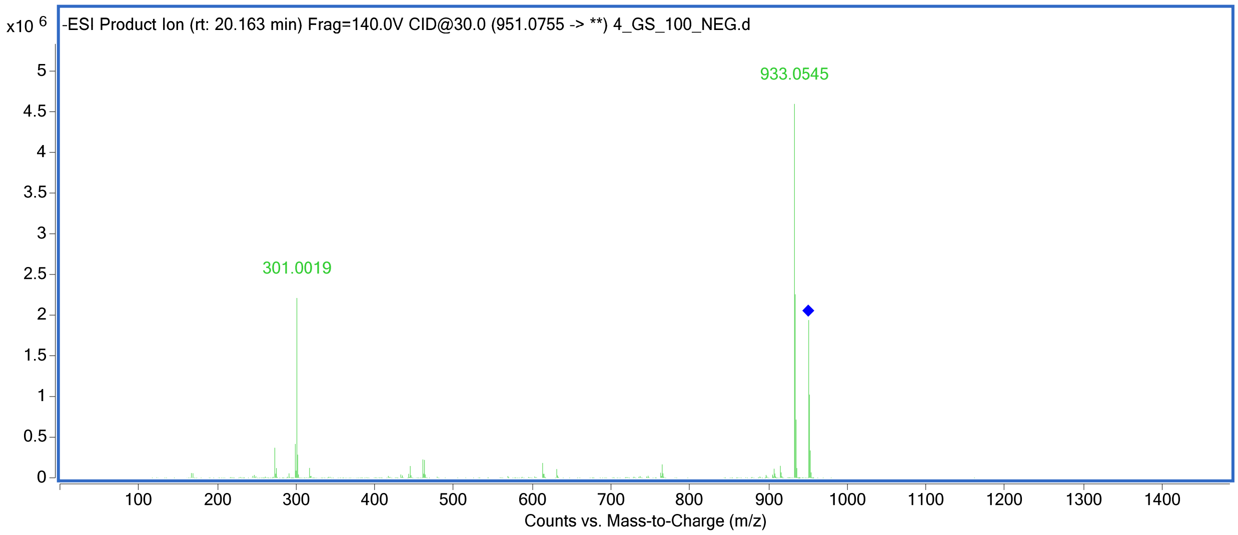

Compound_12_GS:

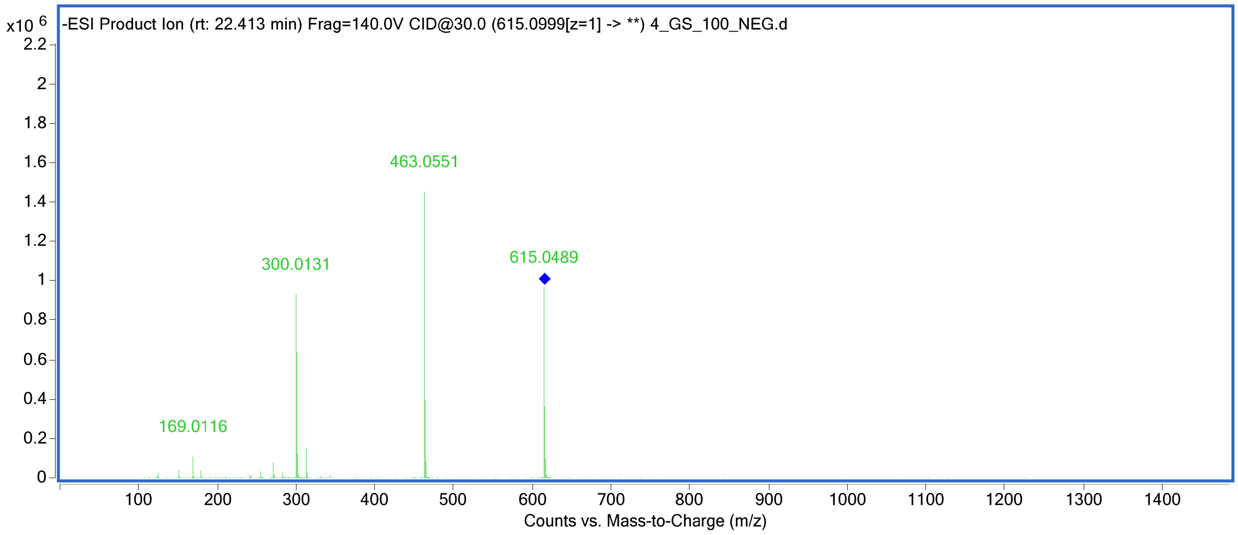

Compound_13_GS:

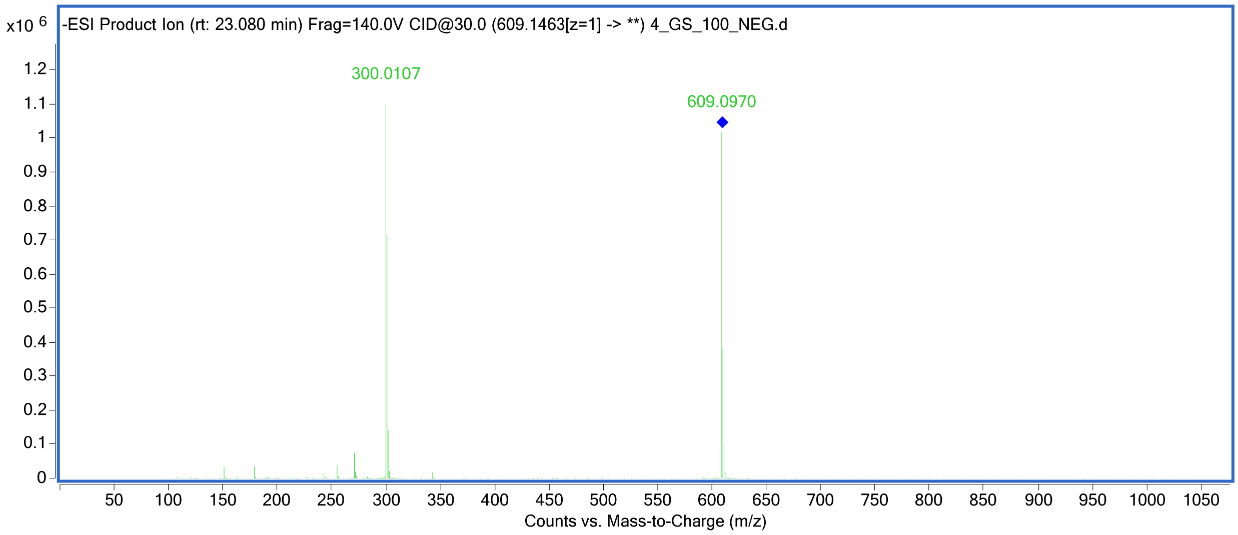

Compound_14_GS:

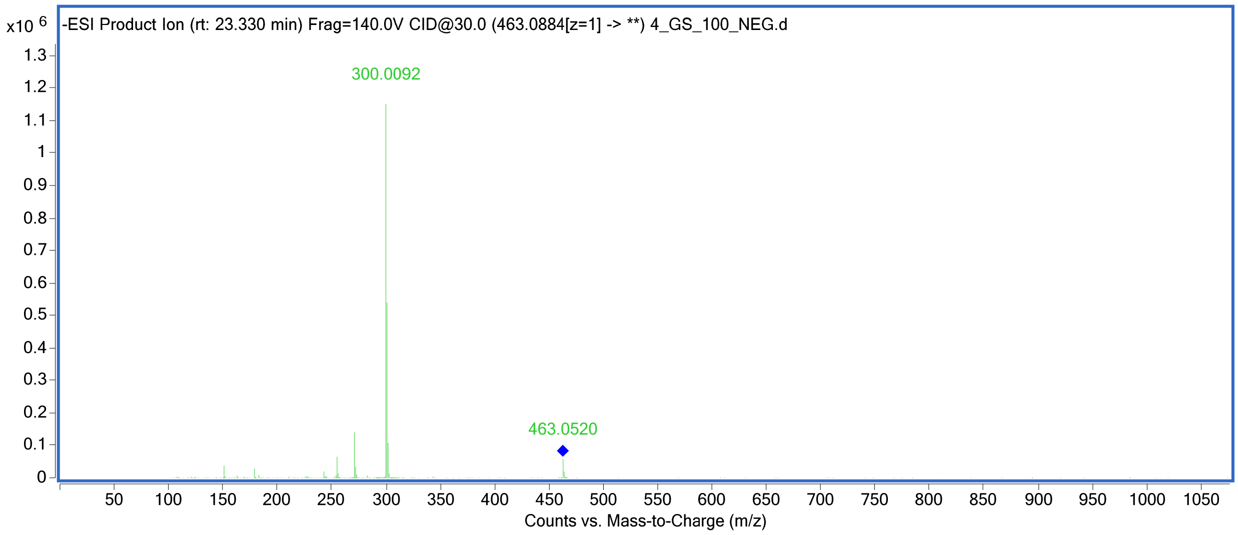

Compound_15_GS:

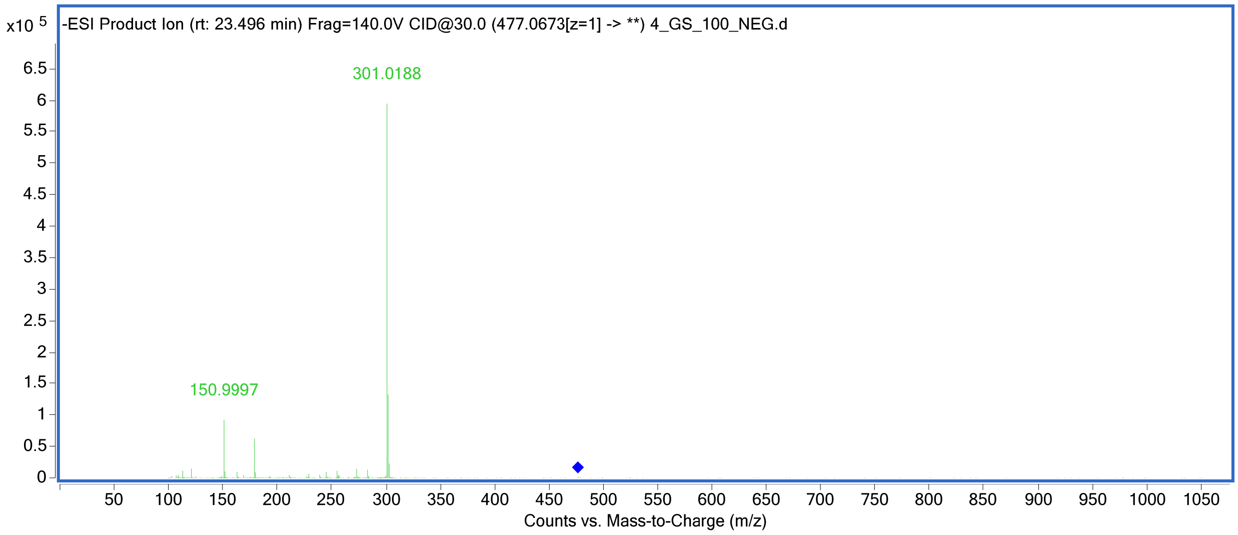

Compound_16_GS:

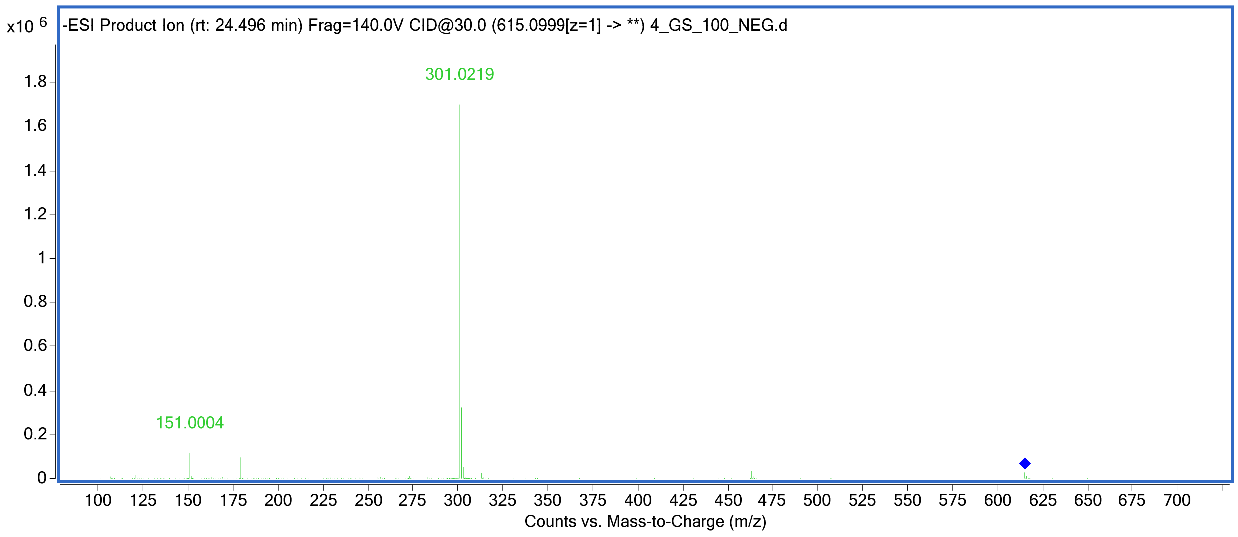

Compound_17_GS:

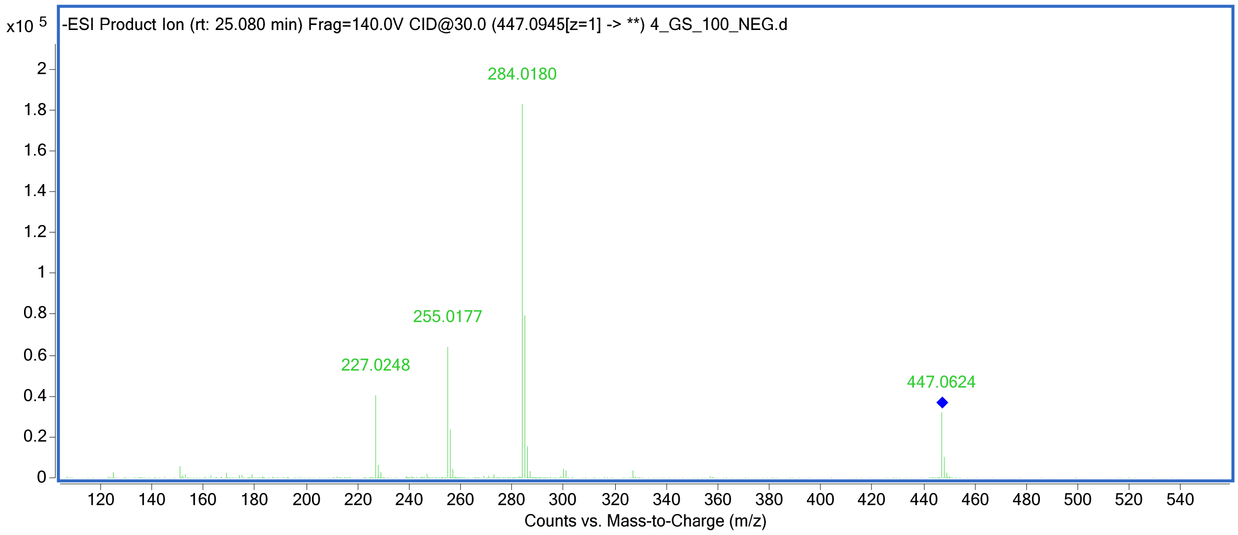

Figure S2. MS/MS product ion spectra obtained by LC–MS/MS analysis of extracts from *Geranium sanguineum*.

Table S2. Results of LC-MS/MS analysis of *Geranium sanguineum* (GS) extracts.

**Number of GS Compound**	Tentative assignment	Retention time [min]	Formula	Molecular Ion [m/z] $[M-H]^-$	Error [ppm]	MS/MS fragments [m/z]	MSI level of annotation
1	Quinic acid	1.747	$C_7H_{12}O_6$	191.0557	-2.14	171.0223; 127.0346; 109.0246	2
2	Gallic acid	3.230	$C_7H_6O_5$	169.0137	-3.22	125.0224	2
3	Galloylquinic acid	4.030	$C_{14}H_{16}O_{10}$	343.0648	-6.60	191.0543	2
4	Galloylshikimic acid	12.246	$C_{14}H_{14}O_9$	325.0564	-0.32	169.0142; 125.0236	2
5	Methyl gallate	13.696	$C_8H_8O_5$	183.0295	-2.16	168.0055; 124.0162	2
6	Di-galloylo-quinic acid	15.062	$C_{21}H_{20}O_{14}$	495.0781	0.14	343.0631; 191.0543	2
7	Brevifolincarboxylic acid	17.046	$C_{13}H_8O_8$	291.0144	-0.82	247.0233; 219.0286; 191.0337; 173.0236; 145.0289; 119.0502	2
8	HDDP-galloyl-glucose isomer 1- (Corilagin structure)	18.246	$C_{27}H_{22}O_{18}$	633.0739	0.89	463.0525; 300.9994	3
9	3,5-Di-O-galloylshikimic acid	18.629	$C_{21}H_{18}O_{13}$	477.0673	-0.34	325.0516; 169.0125; 125.0235	2
10	Methyl di-galloyl glucoside	19.246	$C_{21}H_{22}O_{14}$	497.0935	-0.36	345.0702; 313.0446; 183.0252; 169.0098	2
11	Geraniin	20.163	$C_{41}H_{28}O_{27}$	951.0755	1.03	933.0545; 301.0019	2

12	Quercetin 3-O galloylgalactoside	22.413	$C_{28}H_{24}O_{16}$	615.0999	1.20	463.0551; 300.0131; 169.0116	2
13	Rutin	23.107	$C_{27}H_{30}O_{16}$	609.1470	1.46	300.0107	2
14	Quercetin 3-O Glucoside	23.330	$C_{21}H_{20}O_{12}$	463.0884	0.43	300.0092	2
15	Quercetin 3-O Glucuronide	23.496	$C_{21}H_{18}O_{13}$	477.0673	-0.34	301.0188; 178.9922; 150.9997;	2
16	Quercetin 7-O galloylgalactoside	24.496	$C_{28}H_{24}O_{16}$	615.0999	1.20	301.0219; 178.9934; 151.0004	2
17	Kaempferol 3-O-glucoside	25.080	$C_{21}H_{20}O_{11}$	447.0924	-1.98	284.0180; 255.0177; 227.0248	2

Compound_1_GM:

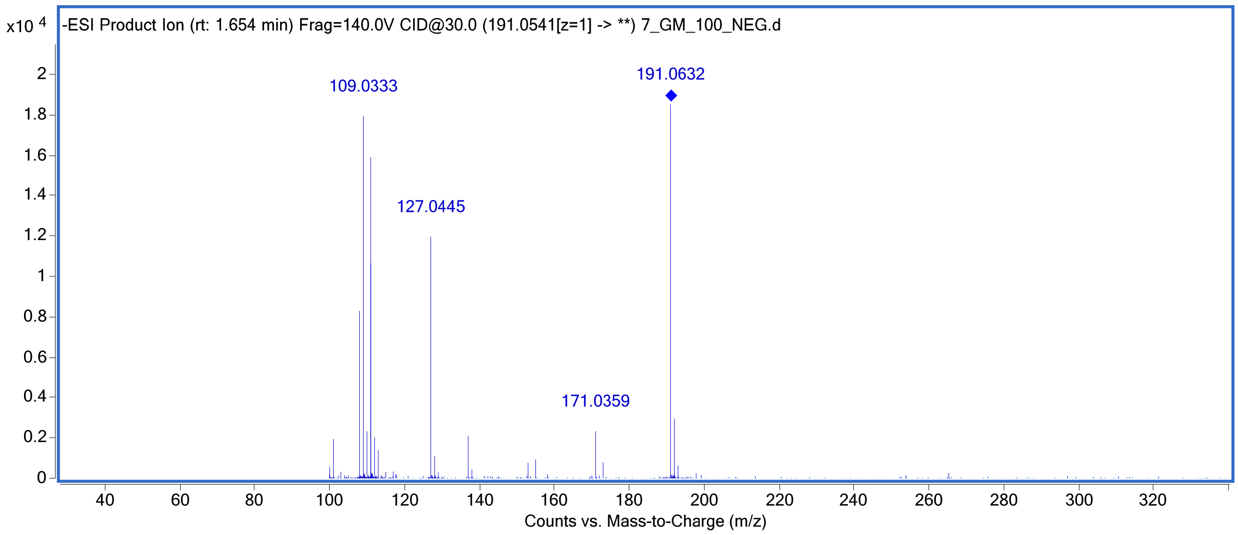

Compound_2_GM:

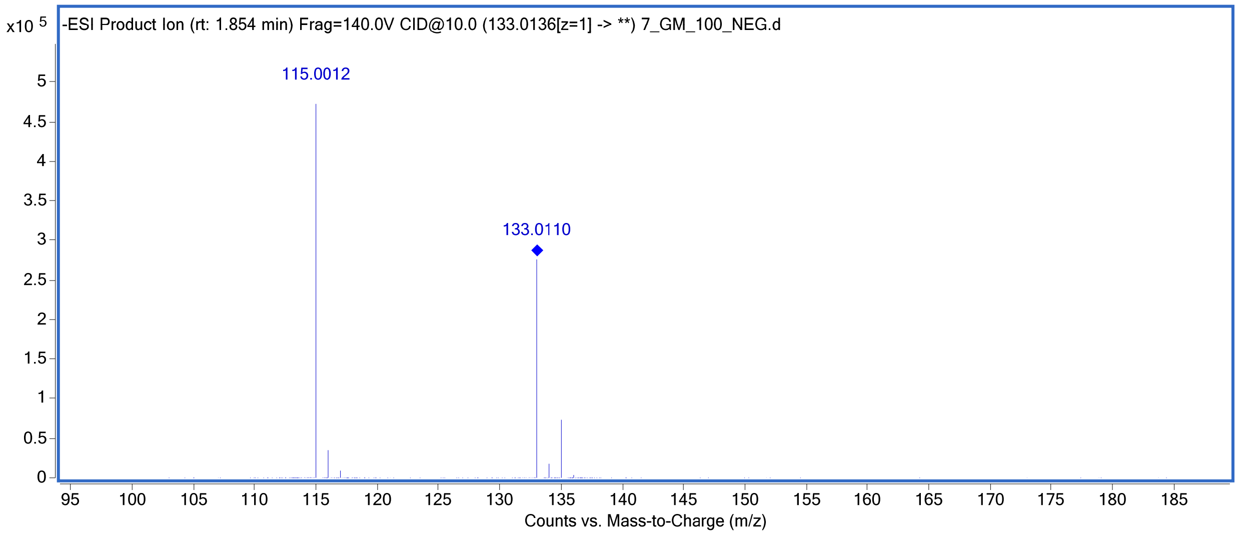

Compound_3_GM:

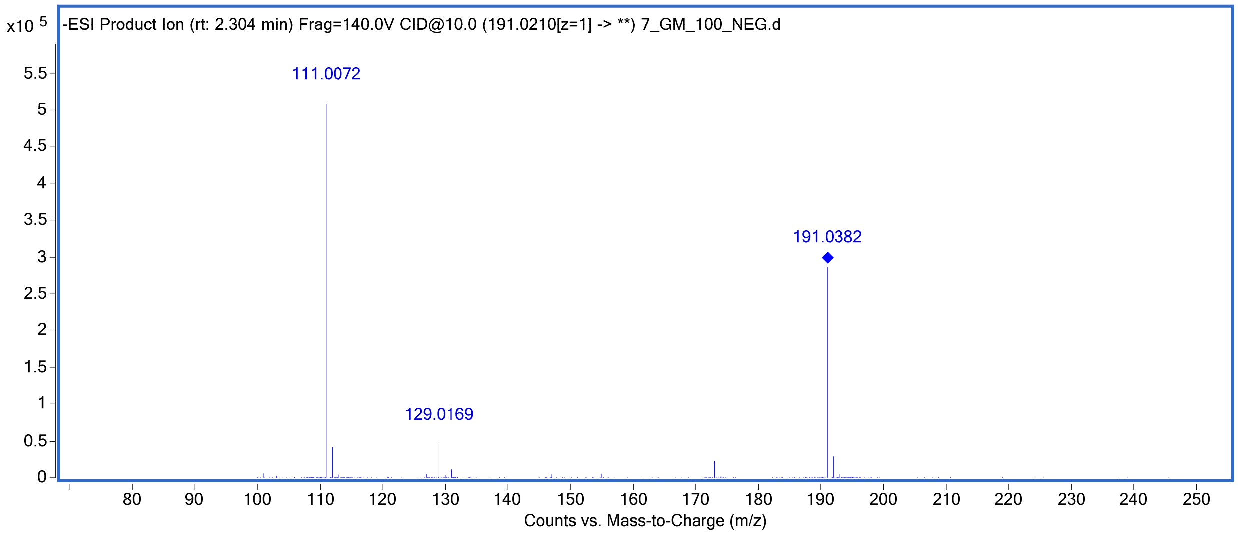

Compound_4_GM:

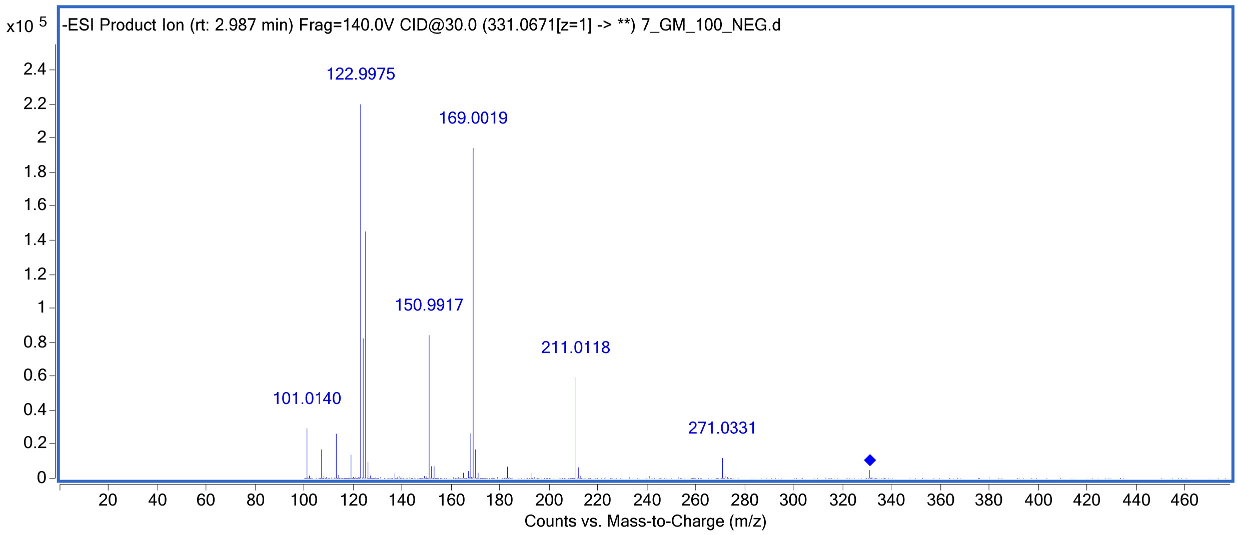

Compound_5_GM:

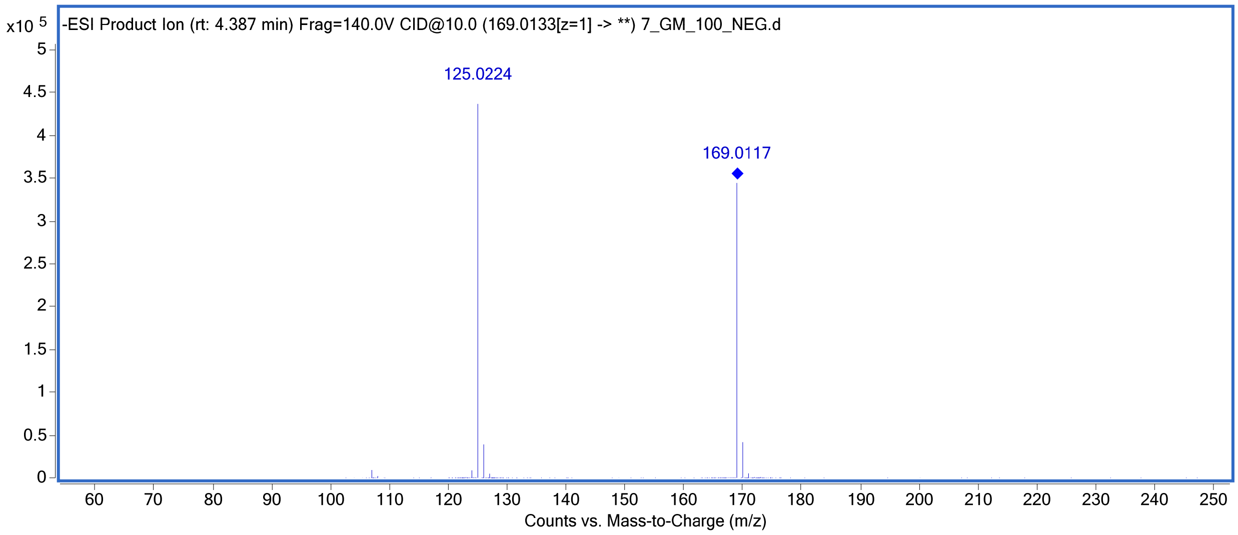

Compound_6_GM:

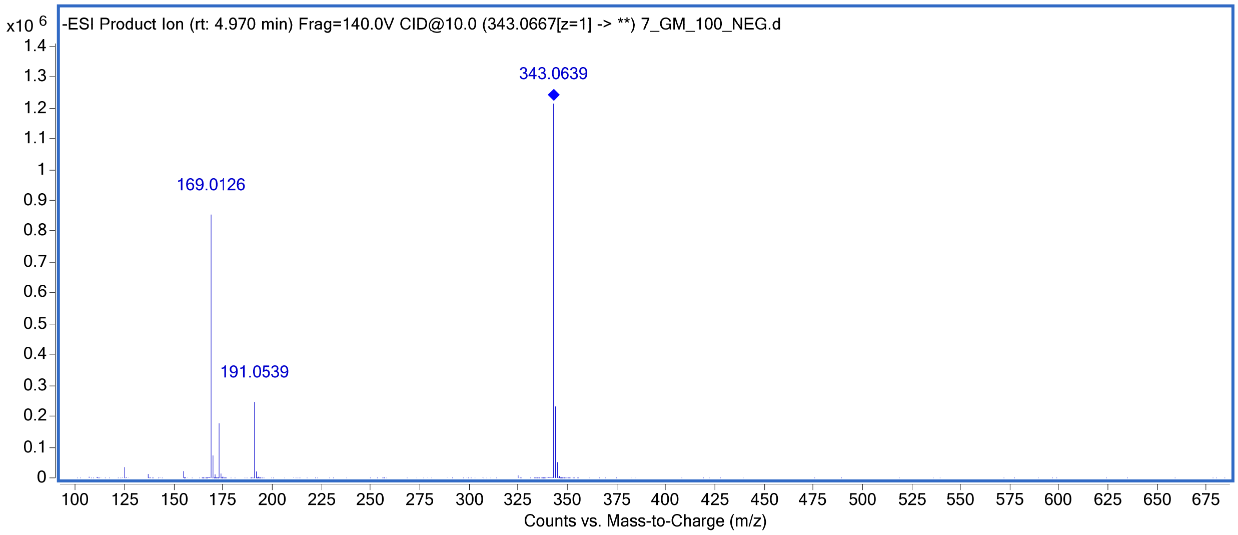

Compound_7_GM:

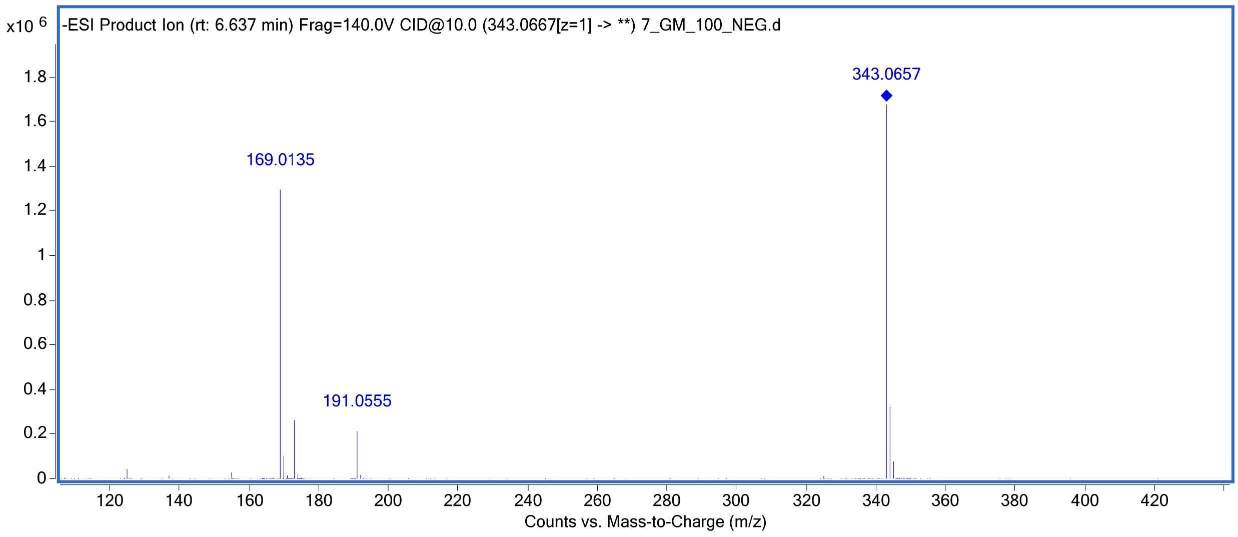

Compound_8_GM:

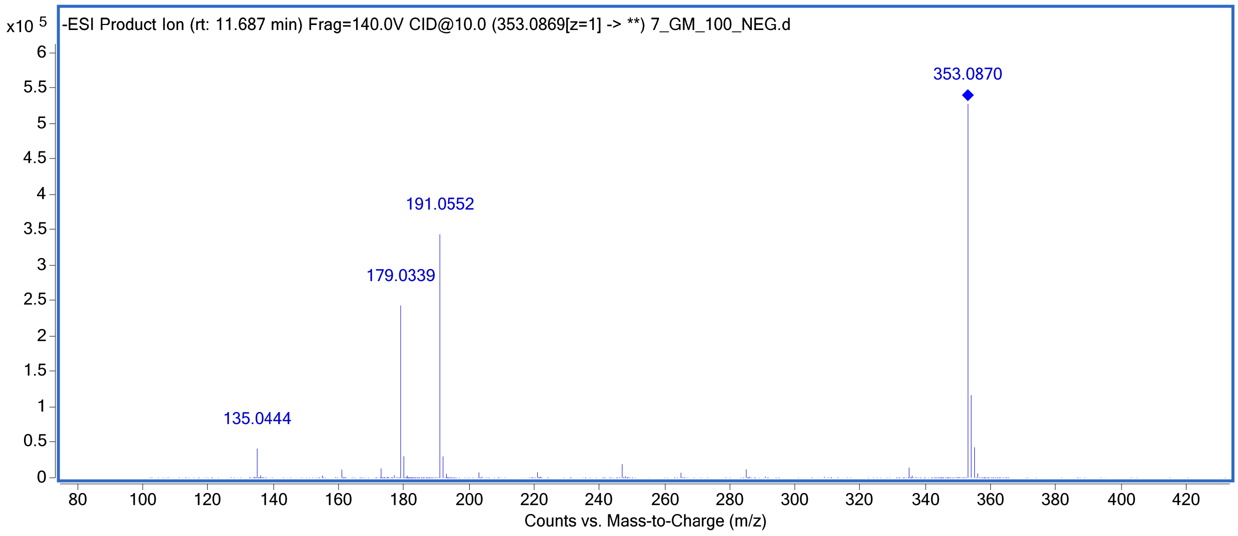

Compound_9_GM:

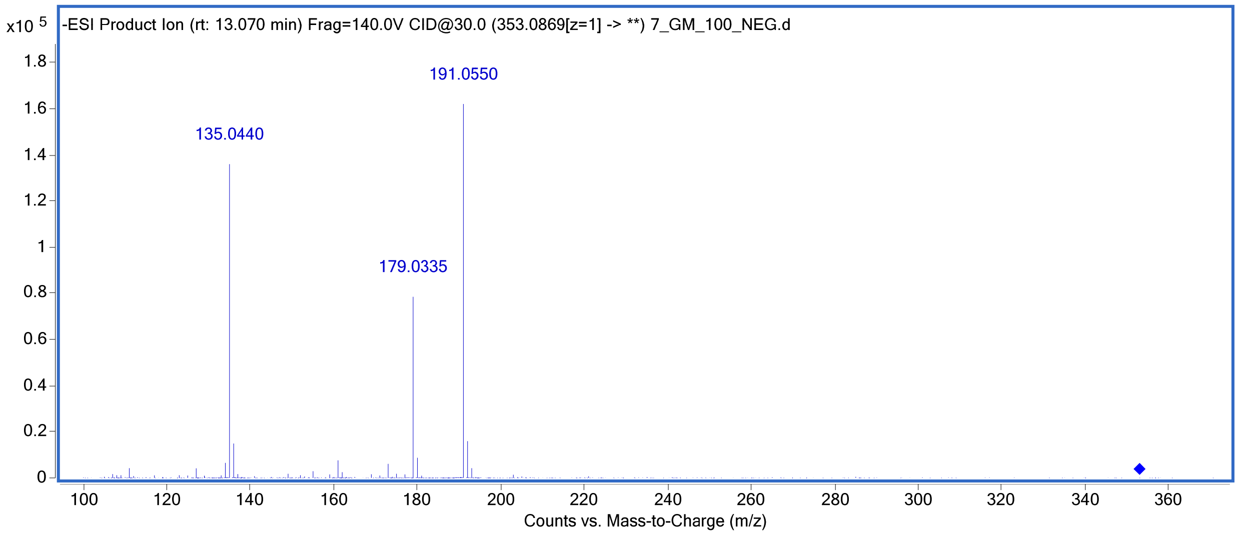

Compound_10_GM:

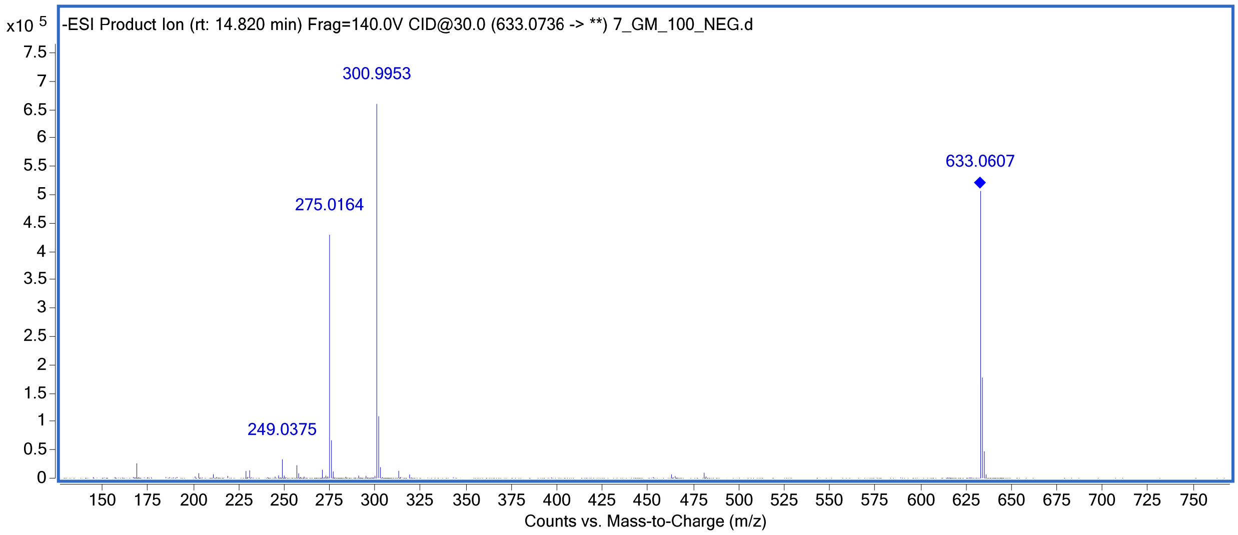

Compound_11_GM:

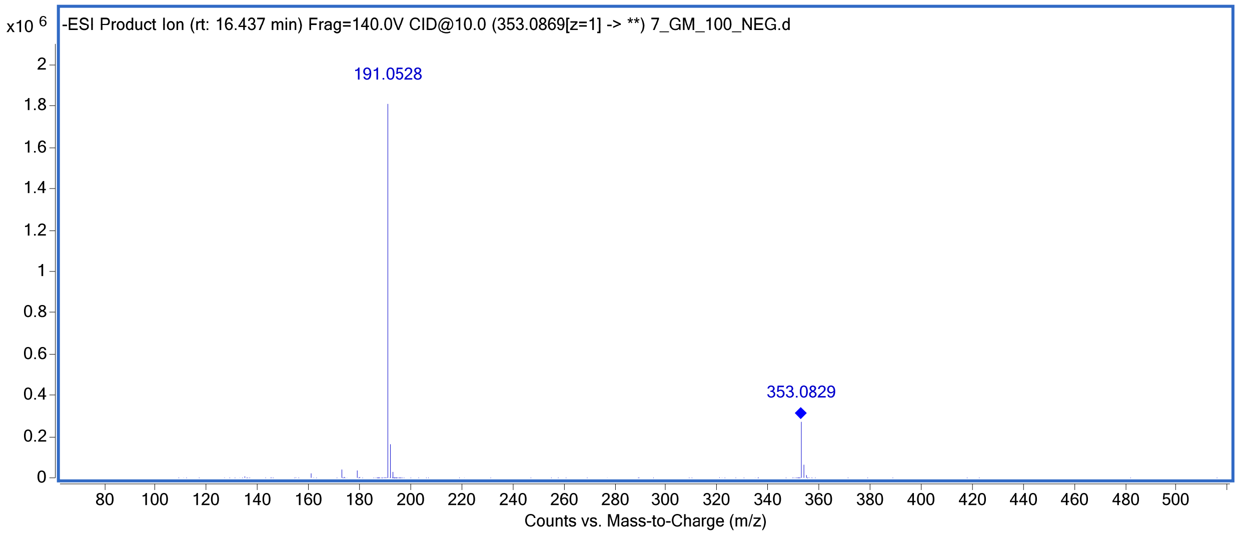

Compound_12_GM:

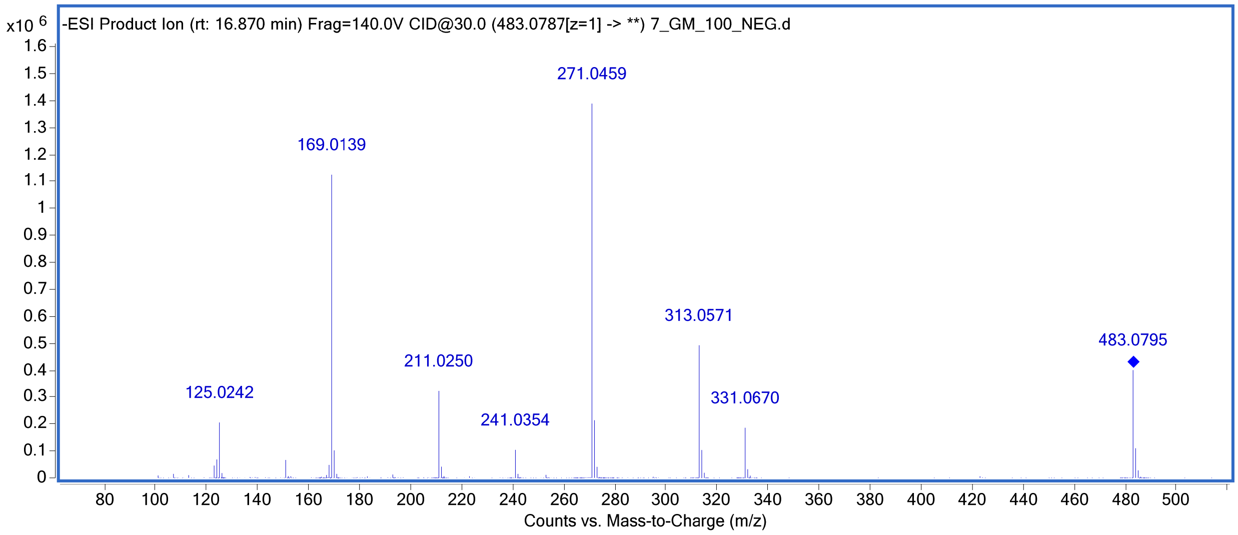

Compound_13_GM:

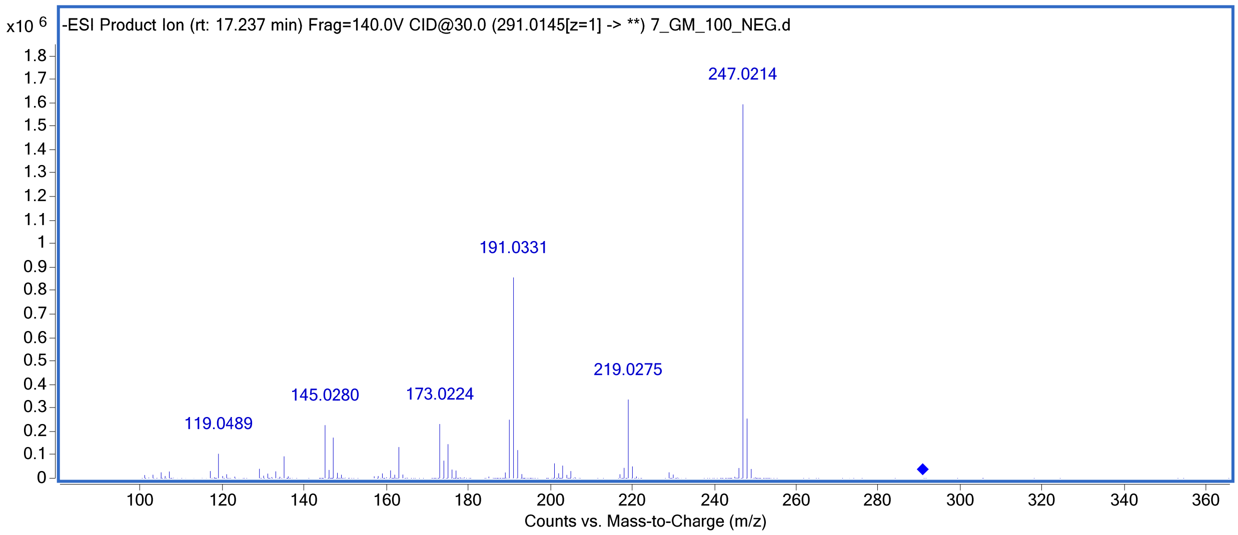

Compound_14_GM:

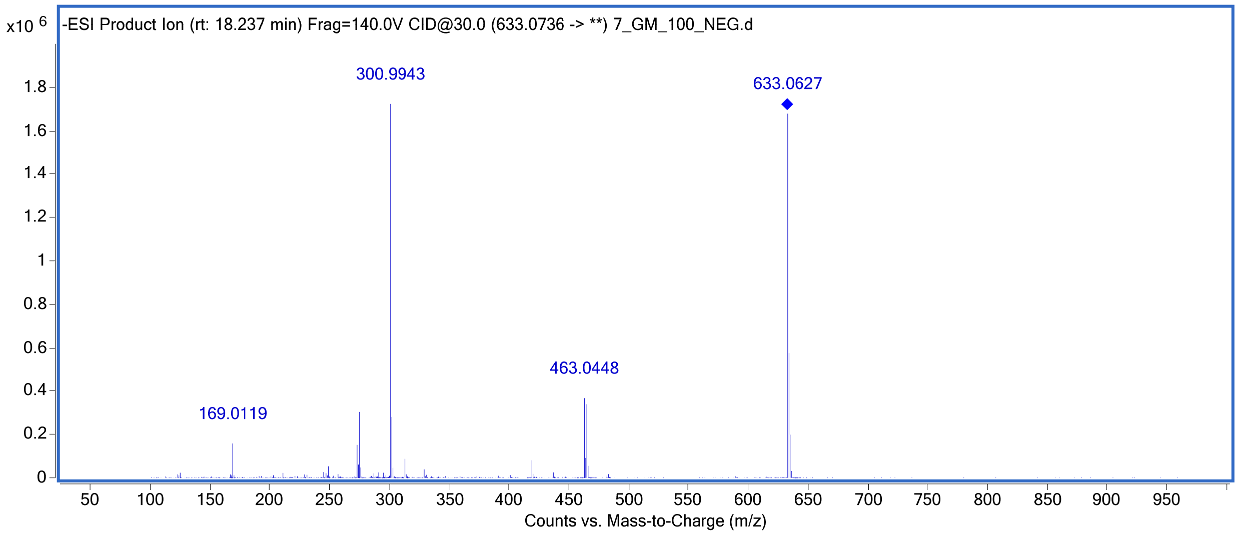

Compound_15_GM:

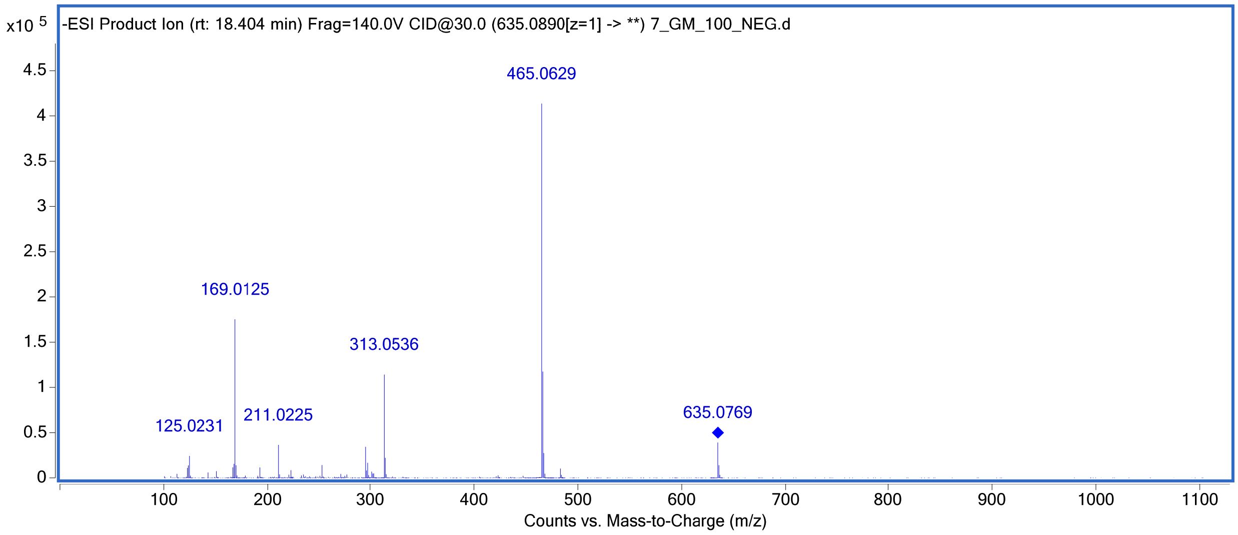

Compound_16_GM:

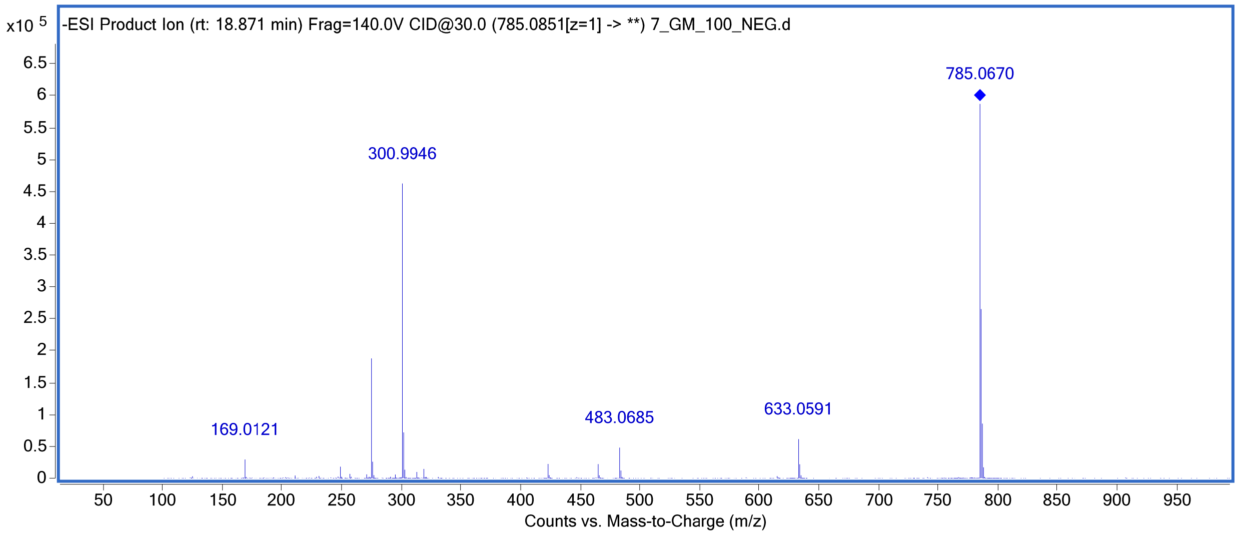

Compound_17_GM:

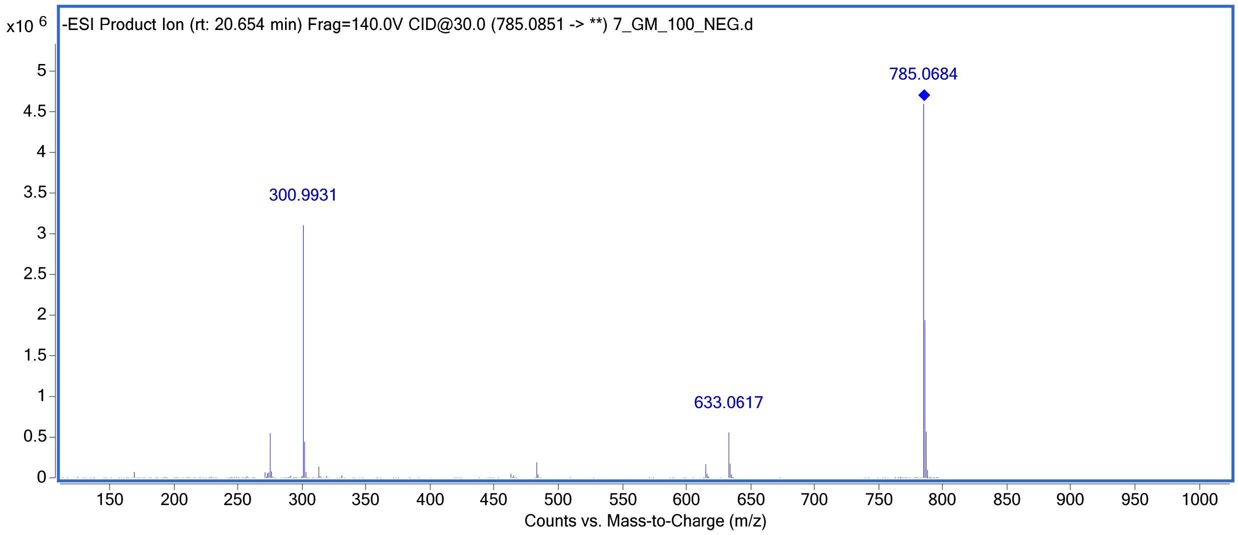

Compound_18_GM:

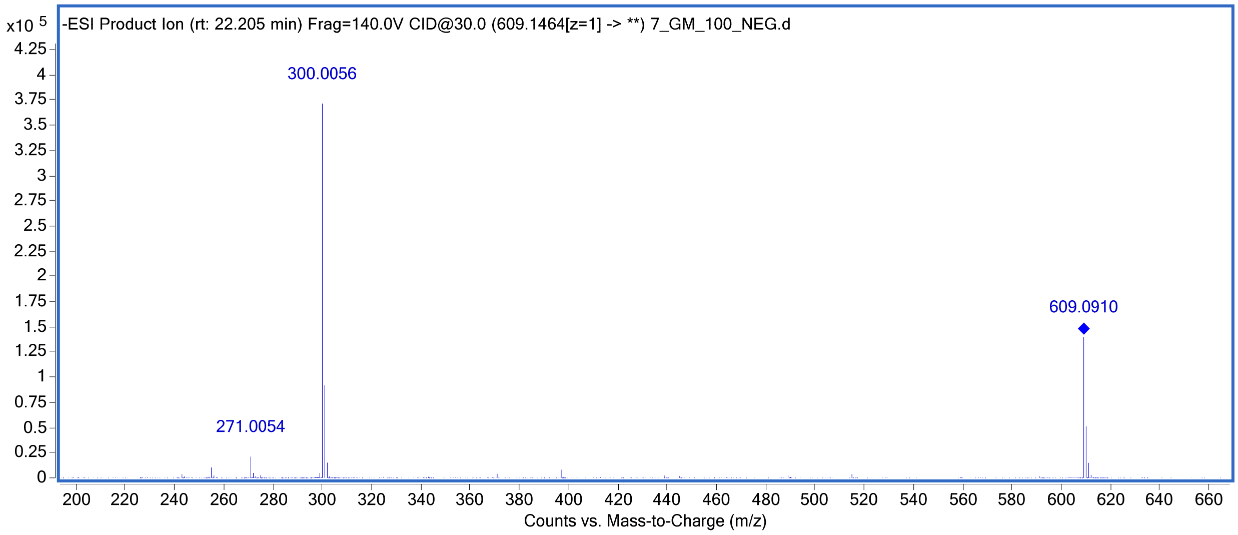

Compound_19_GM:

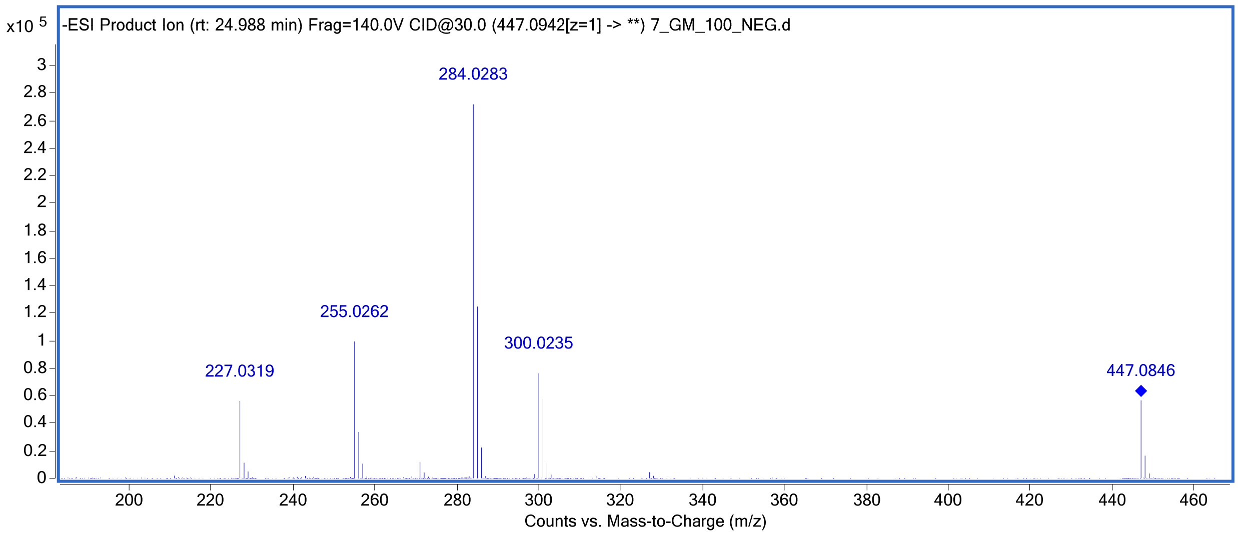

Compound_20_GM:

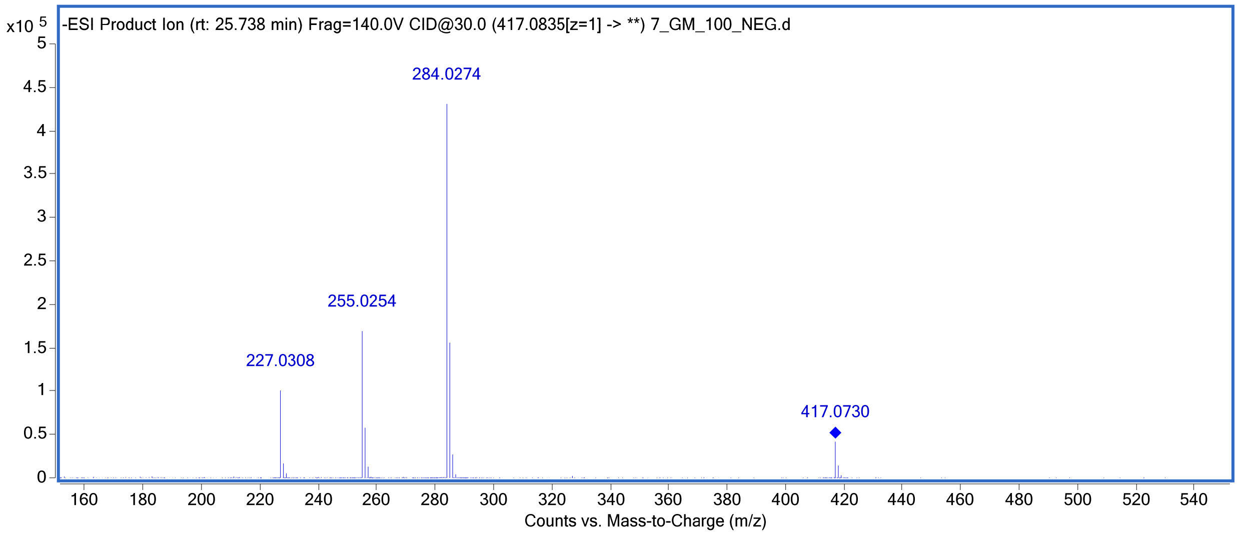

Compound_21_GM:

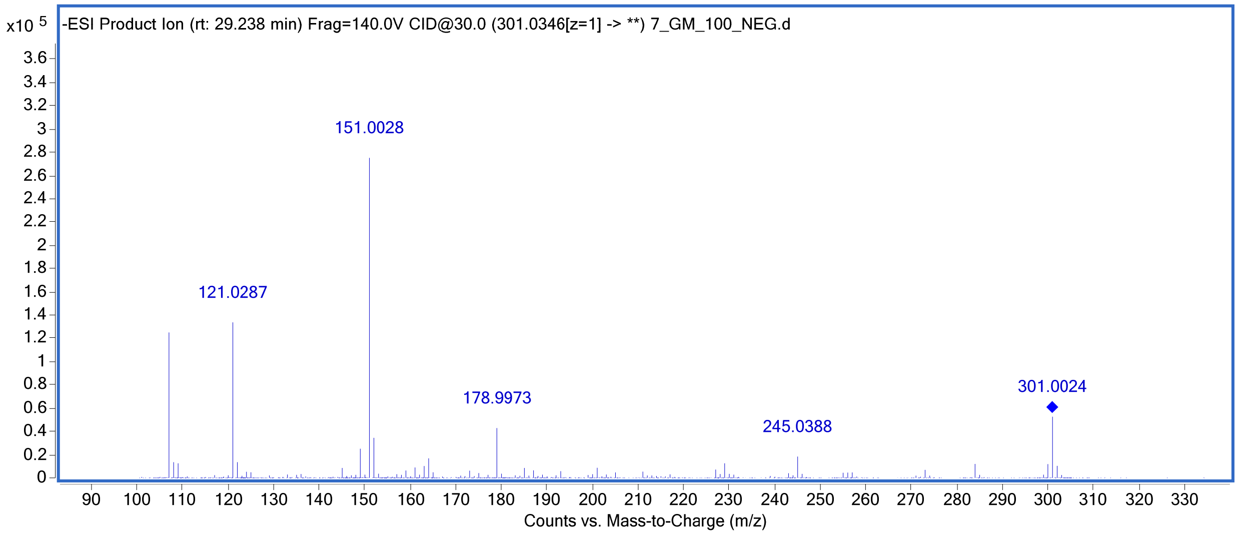

Compound_22_GM:

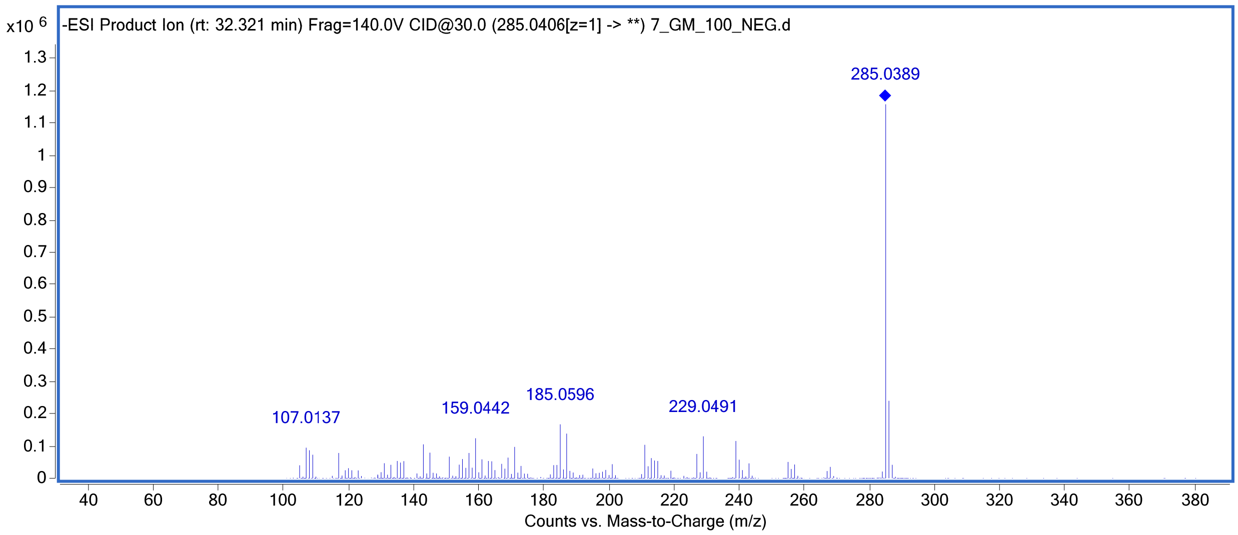

Compound_23_GM:

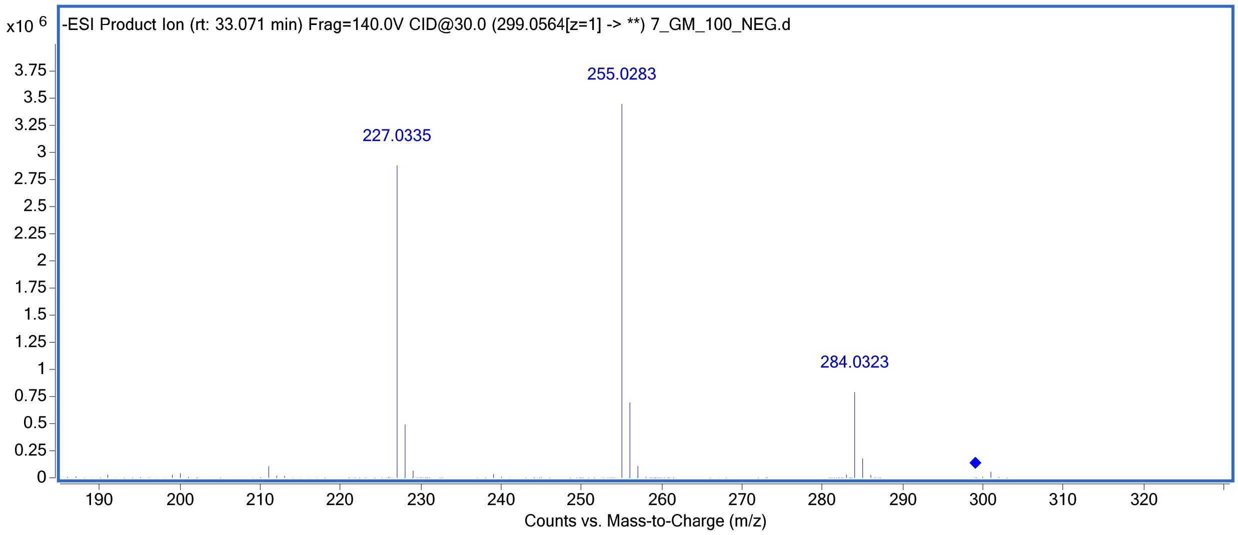

Compound_24_GM:

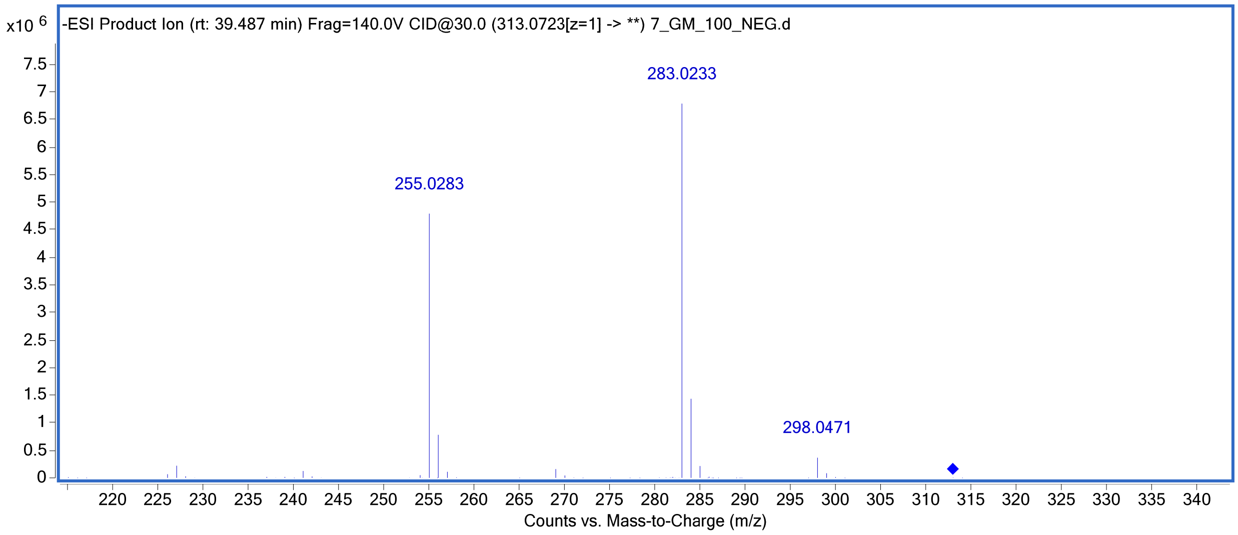

Figure S3. MS/MS product ion spectra obtained by LC–MS/MS analysis of extracts from *Geranium macrorrhizum*.

Table S3. Results of LC-MS/MS analysis of *Geranium macrorrhizum* extracts.

Number of GM Compound	Tentative assignment	Retention time [min]	Formula	Molecular Ion [m/z] [M-H]-	Error [ppm]	MS/MS fragments [m/z]	MSI level of annotation
1	Quinic acid	1.654	$C_7H_{12}O_6$	191.0541	-10.47	171.0359; 127.0445; 109.0333	2
2	Malic acid	1.854	$C_4H_6O_5$	133.0136	-4.83	115.0012	2
3	Citric acid	2.304	$C_6H_8O_7$	191.0210	6.63	129.0169; 111.0072	2
4	Glucogallin	3.536	$C_{13}H_{16}O_{10}$	331.0671	0.09	271.0331; 211.0118; 169.0019; 150.9917; 122.9957	2
5	Gallic acid	4.153	$C_7H_6O_5$	169.0133	-5.57	125.0224	2
6	Galloylquinic acid isomer 1	4.970	$C_{14}H_{16}O_{10}$	343.0667	-1.08	191.0539; 169.0126	2
7	Galloylquinic acid isomer 2	6.637	$C_{14}H_{16}O_{10}$	343.0667	-1.08	191.0555; 169.0124	2
8	Neochlorogenic acid	11.687	$C_{16}H_{18}O_9$	353.0869	-2.56	191.0552; 179.0339; 135.0444	2
9	Chlorogenic acid	13.070	$C_{16}H_{18}O_9$	353.0869	-2.56	191.0550; 179.0335; 135.0440	2
10	HDDP-galloyl-glu-cose isomer 2- (Corilagin structure)	14.820	$C_{27}H_{22}O_{18}$	633.0736	0.41	300.9953; 275.0164; 249.0375	3
11	Cryptochlorogenic acid	16.437	$C_{16}H_{18}O_9$	353.0849	-8.21	191.0528	2

12	Digallyoylglucose isomer	16.870	$C_{20}H_{20}O_{14}$	483.0787	1.39	331.0670; 313.0571; 271.0459; 241.0354; 211.0250; 169.0139; 125.0242	2
13	Brevifolincarboxylic acid	17.237	$C_{13}H_8O_8$	291.0145	-0.48	247.0214; 219.0275; 191.0331; 173.0234; 145.0280; 119.0489	2
14	HDDP-galloyl-glu-cose isomer 1- (Corilagin structure)	18.237	$C_{27}H_{22}O_{18}$	633.0736	0.41	463.0448; 300.9943; 275.0155 169.0119	2
15	Trigalloylglucose	18.404	$C_{27}H_{24}O_{18}$	635.0890	0.02	465.0629; 313.0536; 211.0225; 169.0125; 125.0231	2
16	di-galloyl-HHDP-glu-coside isomer 1 (Tellimagrandin I structure)	18.871	$C_{34}H_{26}O_{22}$	785.0870	3.44	633.0591; 483.0685; 300.9946; 275.0154; 169.0121	3
17	di-galloyl-HHDP-glu-coside isomer 2 (Tellimagrandin I structure)	20.654	$C_{34}H_{26}O_{22}$	785.0884	5.22	633.0617; 300.9931; 275.0152	2
18	Rutin	22.205	$C_{27}H_{30}O_{16}$	609.1464	0.48	300.0056; 271.0054	2
19	Quercetin 3-O-rhamnoside	24.988	$C_{21}H_{20}O_{11}$	447.0942	2.04	300.0235 284.0283; 255.0262; 227.0319	2
20	Kaempferol 3-O-pentoside	25.738	$C_{20}H_{18}O_{10}$	417.0835	1.86	284.0274; 255.0254;	2

						227.0308;	
21	Quercetin	29.238	$C_{15}H_{10}O_7$	301.0346	-2.57	178.9973; 151.0028; 121.0287; 107.0133	2
22	Kaempferol	32.321	$C_{15}H_{10}O_6$	285.0406	0.48	229.0491 185.0596; 107.0137	2
23	Kaempferide	33.071	$C_{16}H_{12}O_6$	299.0564	0.96	284.0323; 255.0283; 227.0335	2
24	Kaempferol dimethyl ether	39.487	$C_{17}H_{14}O_6$	313.0723	1.71	298.0471; 283.0233; 255.0283	2

Table S4. Validation parameters of the HPLC method used for quantitative analysis of selected phenolic compounds.

Validation parameter	Neochlorogenic acid	Chlorogenic acid	Gallic acid	Rutin
Detection wavelength [nm]	240	240	265	360
Regression model	y = ax	y = ax	y = ax	y = ax
Slope, *a* ± Sa	17.1965 ± 1.3440	22.3050 ± 0.4010	46.3318 ± 1.4335	16.9684 ± 0.5747
Intercept, *b*	insignificant	insignificant	insignificant	insignificant
Standard error of estimation, Sy	3.5949	5.1582	6.4853	19.1042
Correlation coefficient, *r*	0.9847	0.9986	0.9965	0.9967
Linearity [µg/injection]	0.364–3.640	0.400–12.000	0.020–5.000	2.500–40.000
Precision levels [µg]	0.364; 0.728; 1.820	4.000; 6.000; 8.000	0.200; 0.500; 1.000	2.500; 5.000; 10.000
Repeatability, RSD [%]	1.324–2.884	1.040–2.232	0.384–4.141	0.419–2.727
Intermediate precision, RSD [%]	2.360–2.426	1.259–4.334	0.699–4.086	1.913–2.418
Limit of detection, LOD [µg]	0.690	0.763	0.462	3.715
Limit of quantification, LOQ [µg]	2.091	2.313	1.400	11.259

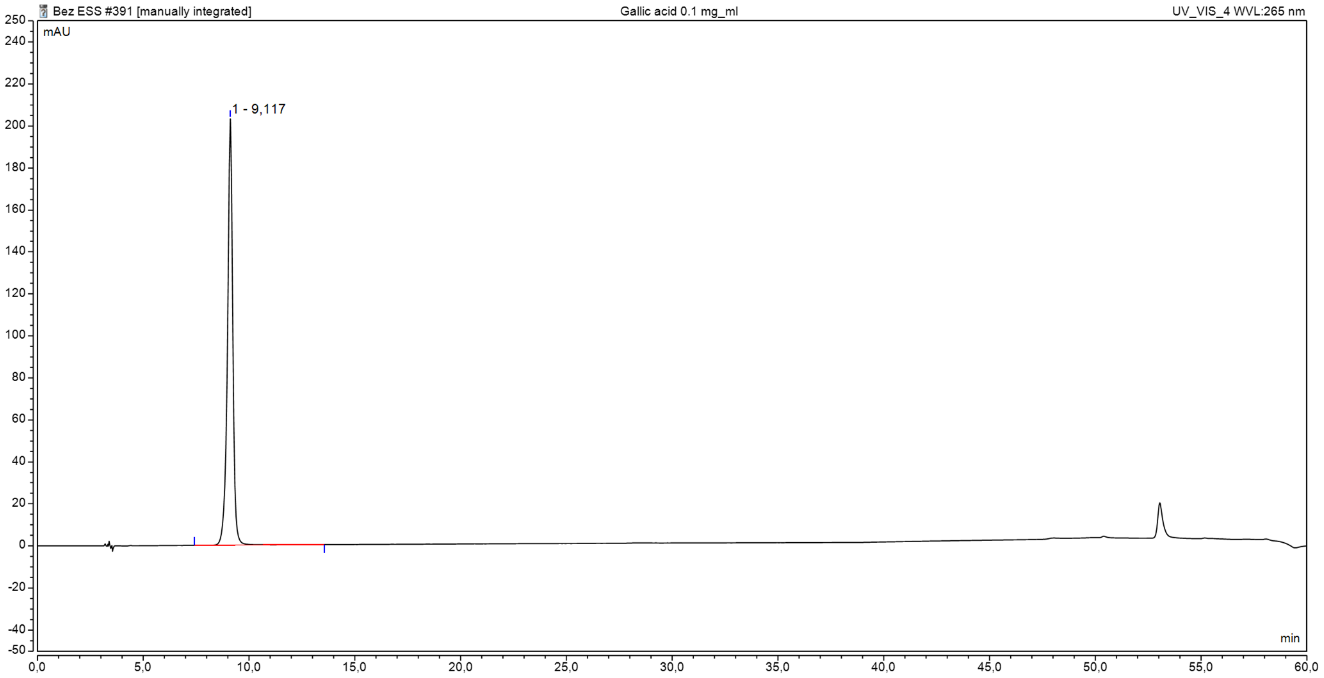

Figure S4. Chromatogram of gallic acid (Rt = 9.117 min; 265 nm).

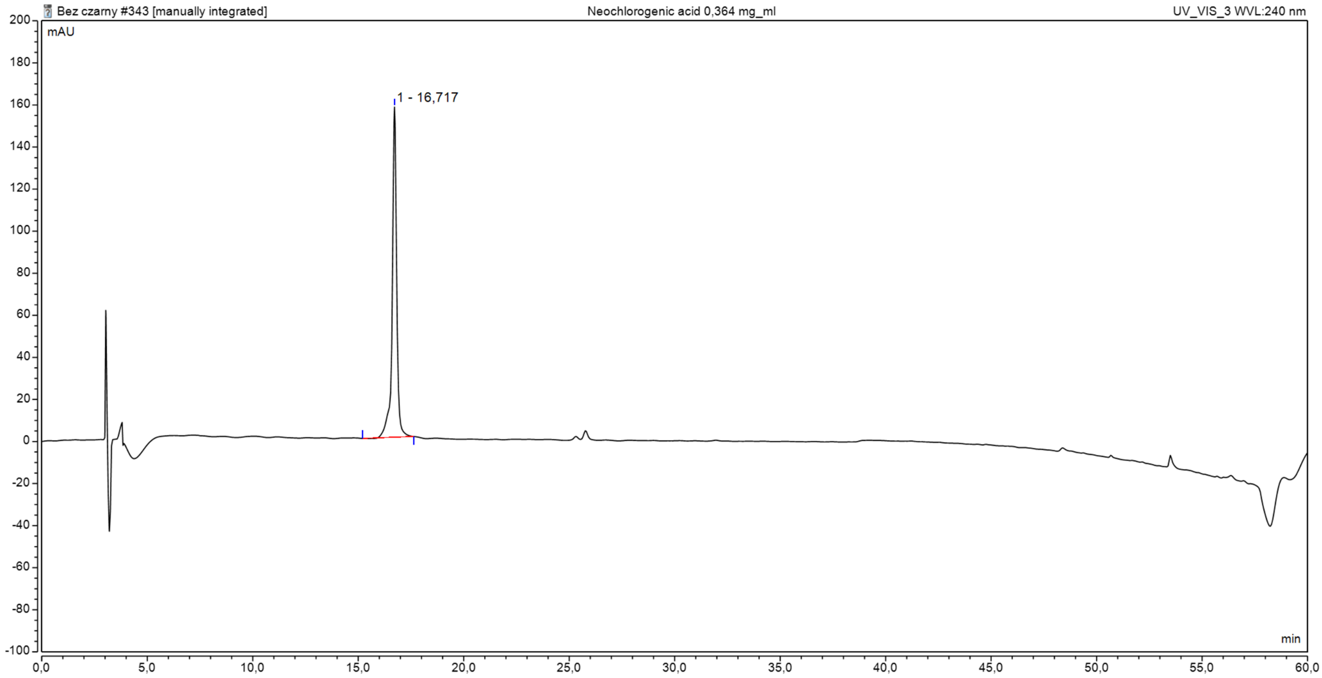

Figure S5. Chromatogram of neochlorogenic acid (Rt = 16.717 min; 240 nm).

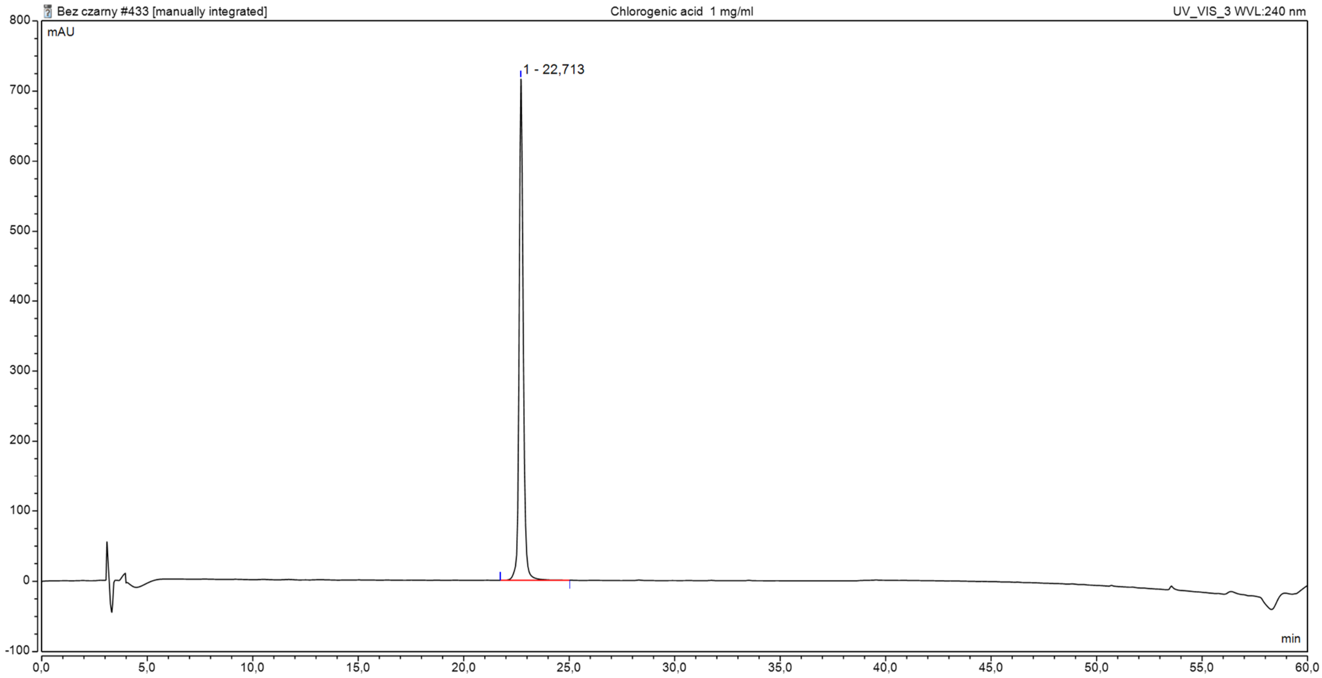

Figure S6. Chromatogram of chlorogenic acid (Rt = 22.713 min; 240 nm).

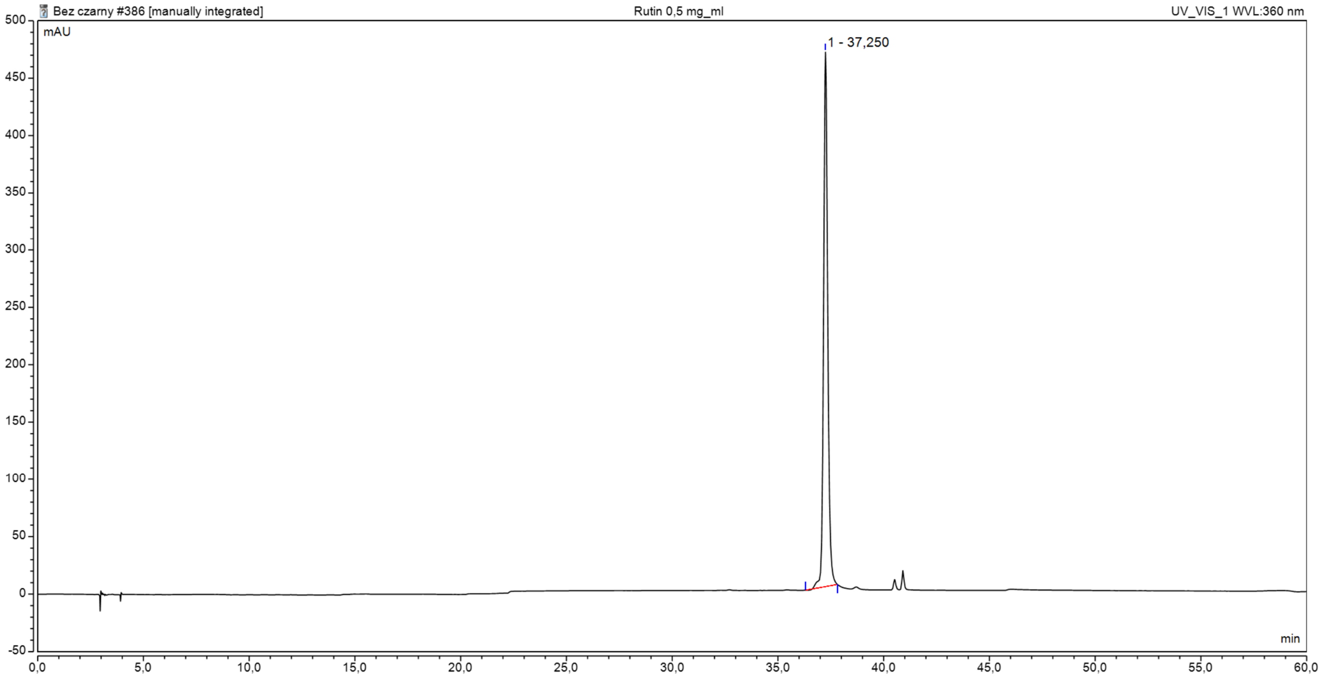

Figure S7. Chromatogram of rutin (Rt = 37.250 min; 360 nm).

Representative HPLC-DAD chromatograms of the investigated *Geranium* extracts are shown at 265 nm to illustrate the general chromatographic profiles of the samples. Quantification of individual analytes was performed at compound-specific wavelengths, as indicated in the standard chromatograms shown above.

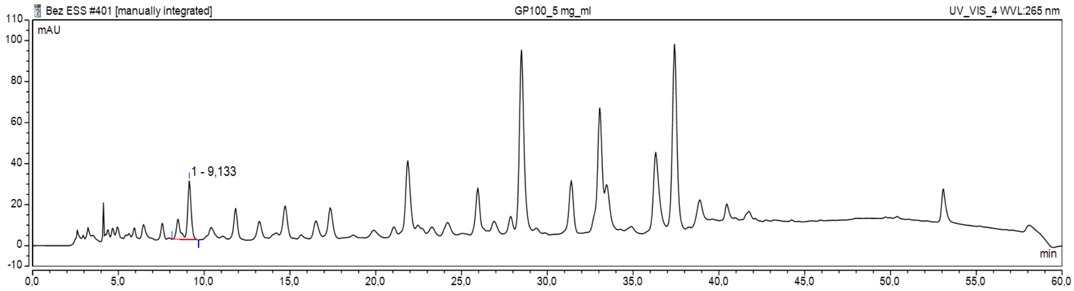

Figure S8. Chromatogram of the aqueous extract (100 °C) from *G. phaeum* leaves; gallic acid (Rt = 9.133 min).

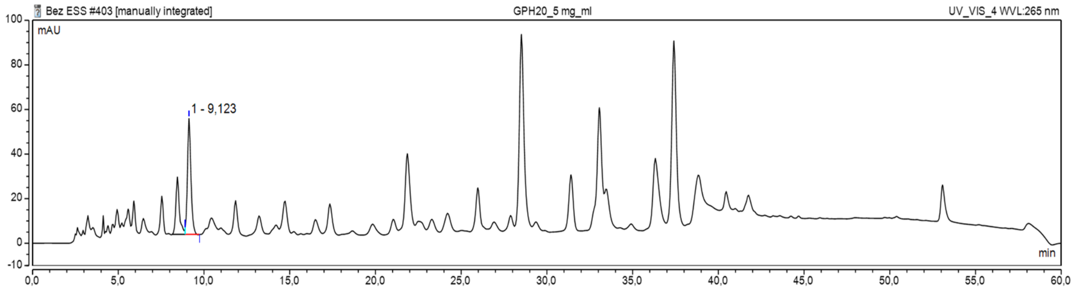

Figure S9. Chromatogram of the aqueous extract (50 °C) from *G. phaeum* leaves; gallic acid (Rt = 9.123 min).

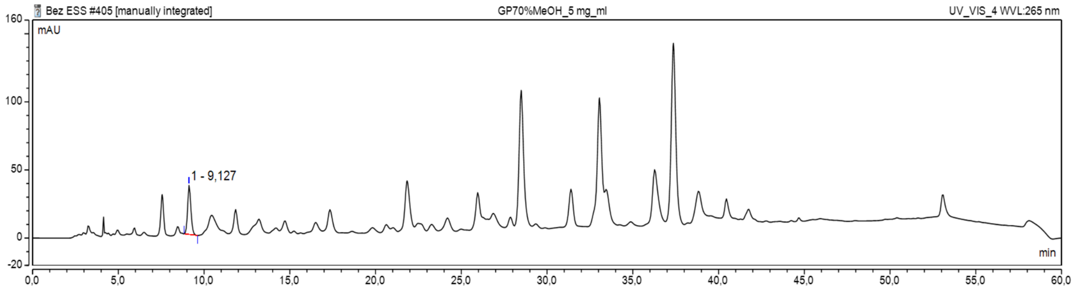

Figure S10. Chromatogram of the 70% methanol extract from *G. phaeum* leaves; gallic acid (Rt = 9.127 min).

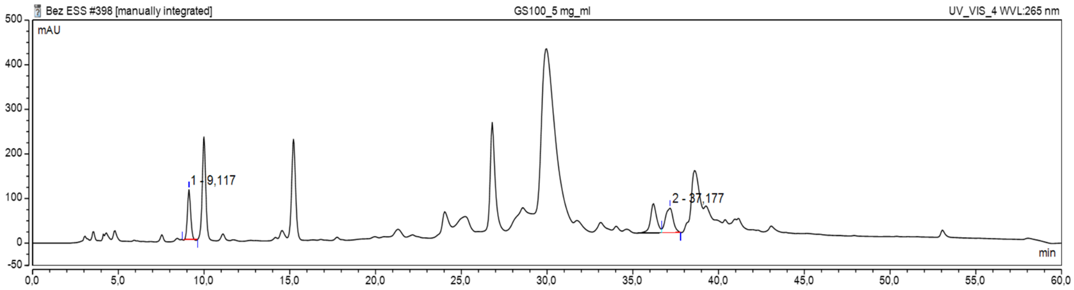

Figure S11. Chromatogram of the aqueous extract (100 °C) from *G. sanguineum* leaves; gallic acid (Rt = 9.117 min.), rutin (Rt = 37.177 min).

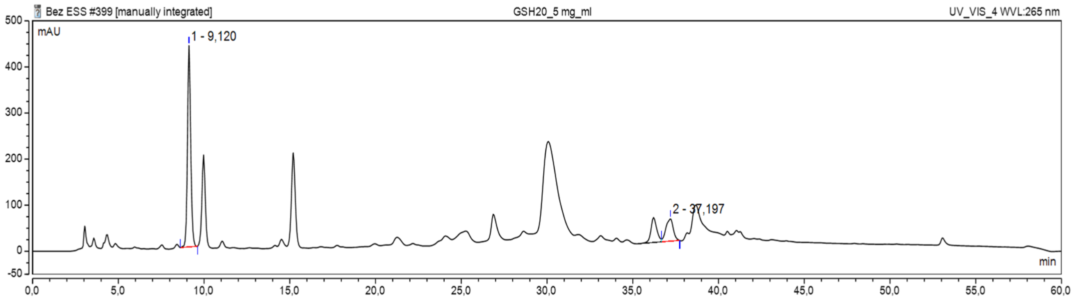

Figure S12. Chromatogram of the aqueous extract (50 °C) from *G. sanguineum* leaves; Gallic acid (Rt = 9.120 min), rutin (Rt = 37.197 min).

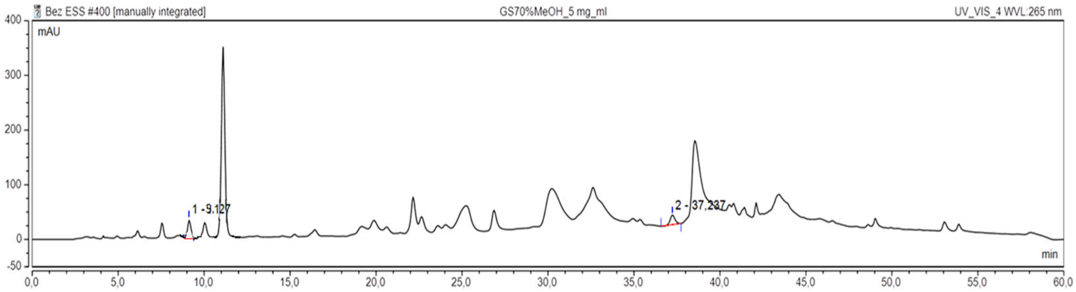

Figure S13. Chromatogram of the 70% methanol extract from *G. sanguineum* leaves; Gallic acid (Rt = 9.127 min), rutin (Rt = 37.237 min).

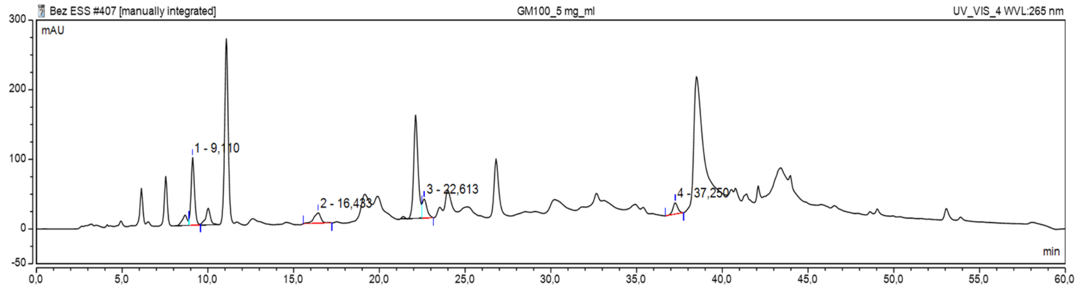

Figure S14. Chromatogram of the aqueous extract (100 °C) from *G. macrorrhizum* laves; Galiic acid (Rt = 9.110 min); neochlorogenic acid (Rt = 16.439 min); chlorogenic acid (Rt = 22.613 min); rutin (Rt = 37.250 min).

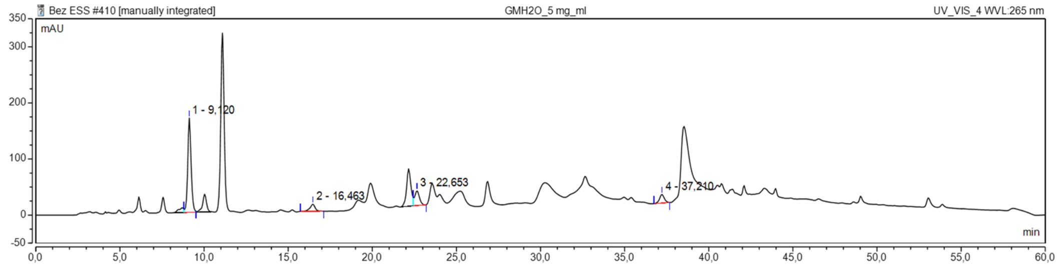

Figure S15. Chromatogram of the aqueous extract (50 °C) from *G. macrorrhizum* leaves; Gallic acid (Rt = 9.120 min); neochlorogenic acid (Rt = 16.463 min); chlorogenic acid (Rt = 22.653 min); rutin (Rt = 37.210 min).

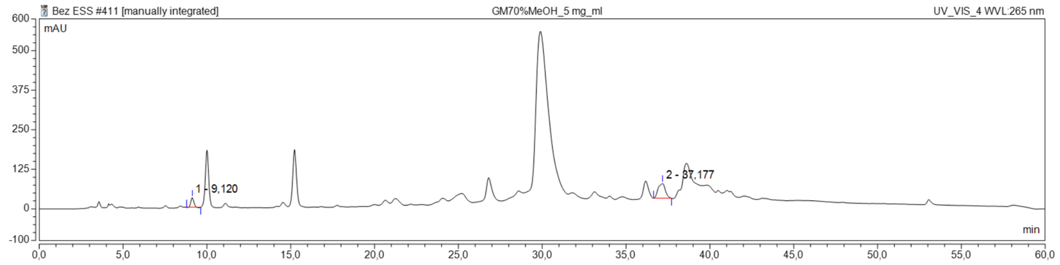

Figure S16. Chromatogram of the 70% methanol extract from *G. macrorrhizum* leaves; Gallic acid (Rt = 9.120 min); rutin (Rt = 37.177 min).

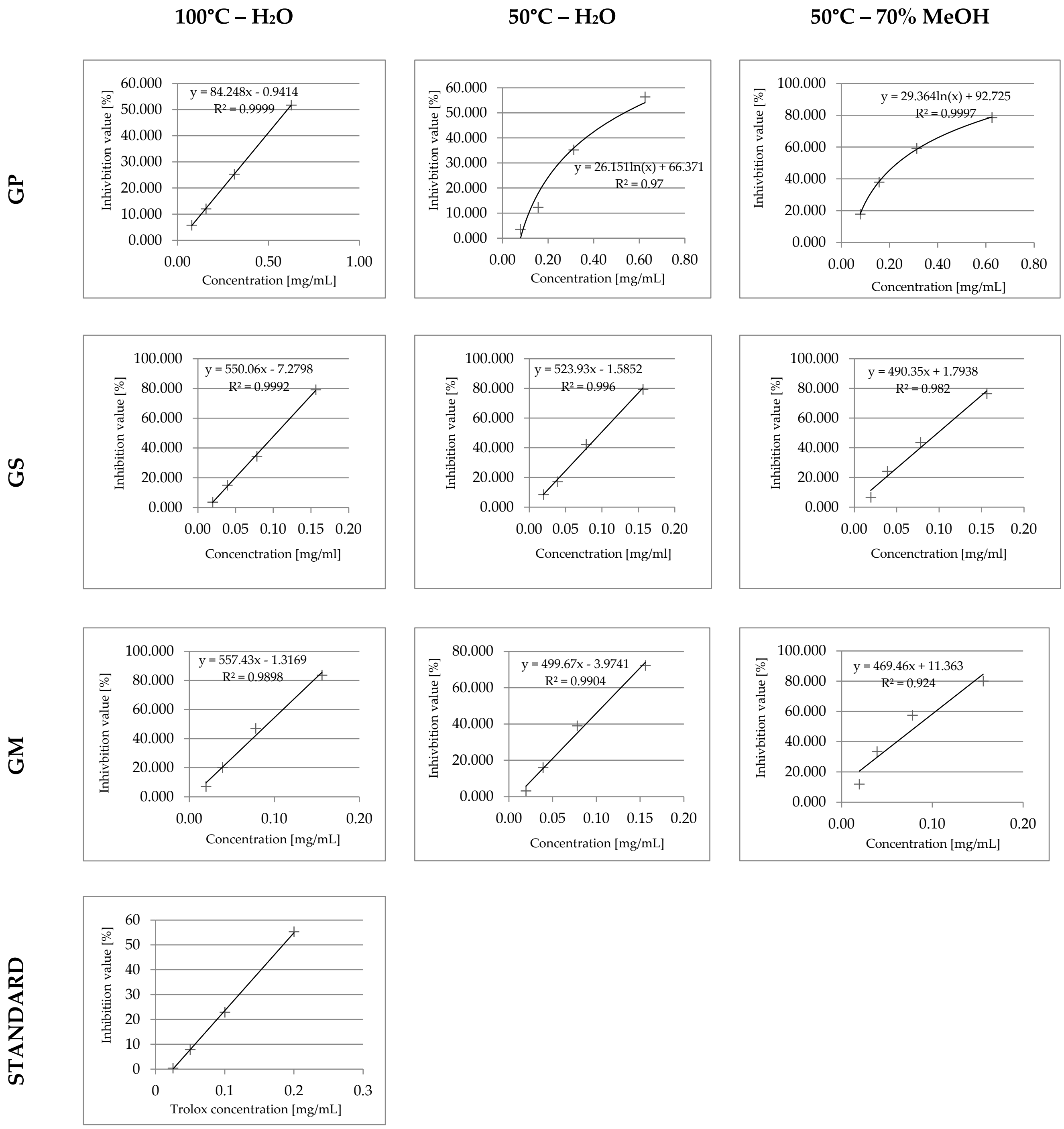

Figure S17. Standard and response curves for **DPPH assay** (standard—Trolox concentration range 0.025–0.2 mg/mL; y = 314.05x – 7.8686; r = 0.9998).

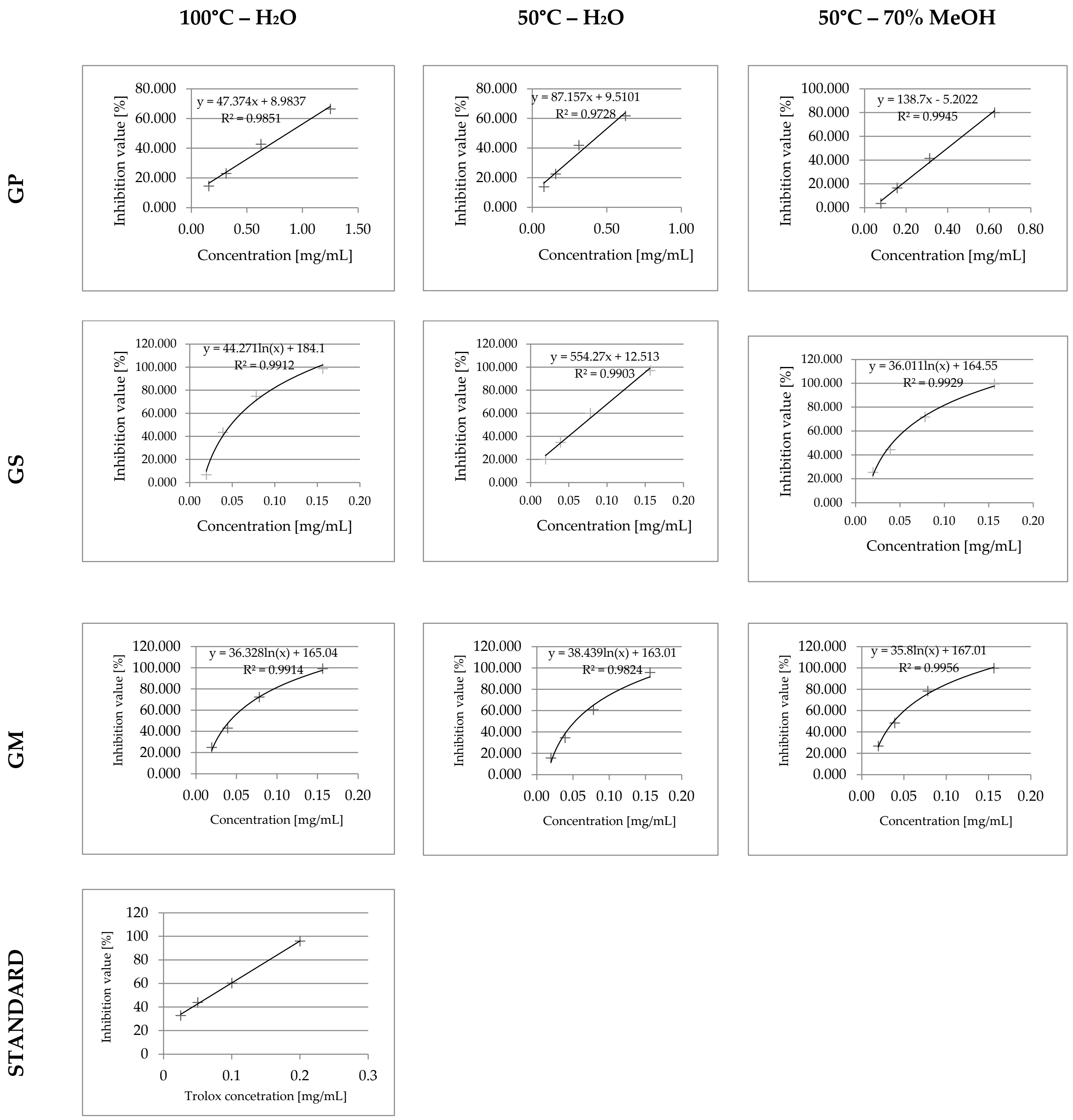

Figure S18. Standard and response curves for **ABTS assay** (standard: Trolox; concentration range 0.025–0.2 mg/mL; y = 356.1x + 24,827; r = 0.9994).

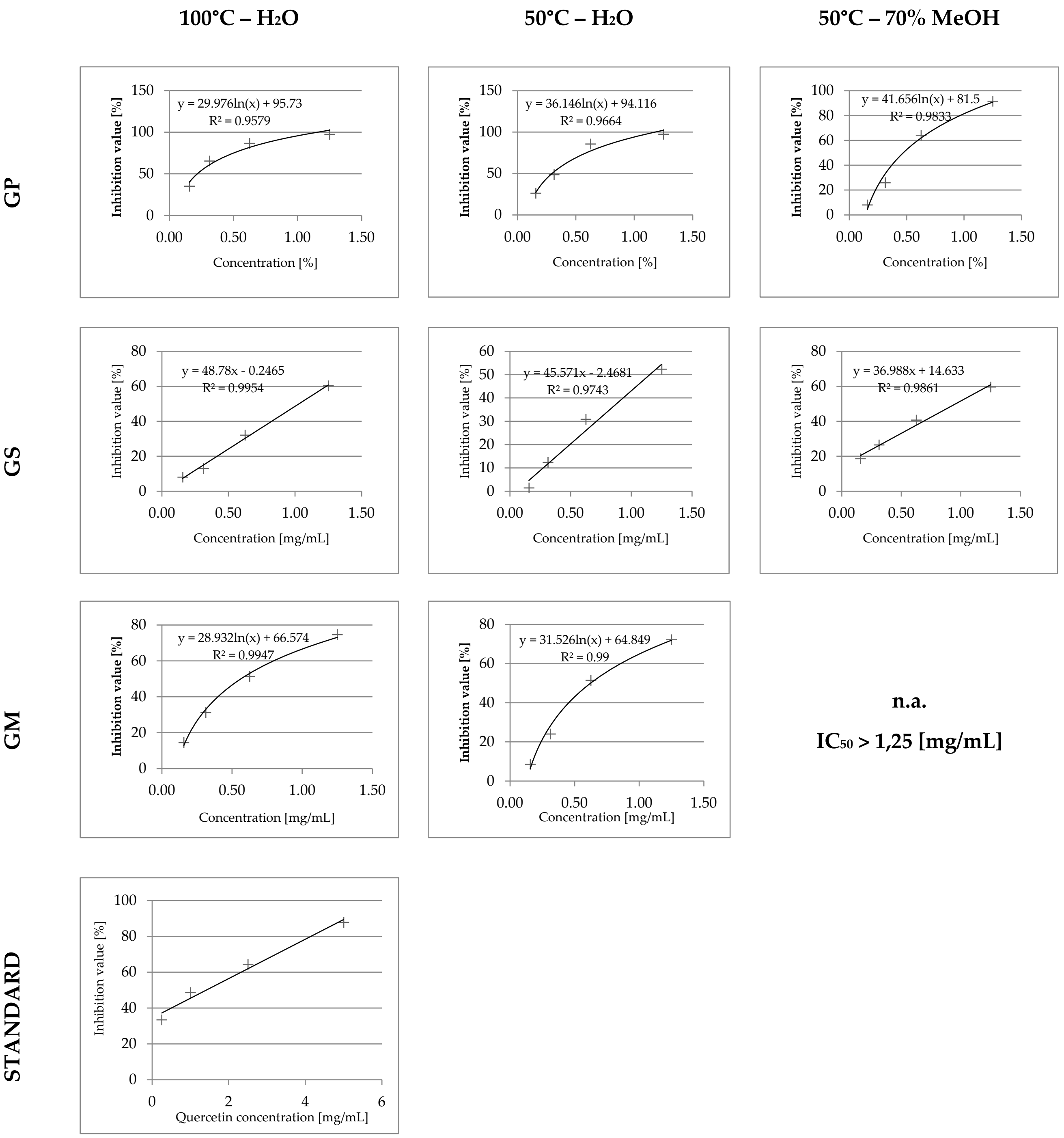

Figure S19. Standard and response curves for: **Fe^{2+} chelation** (standard: Quercetin; concentration range 0.25–5.0 mg/mL; y = 10.985x + 34.533; r = 0.9897).

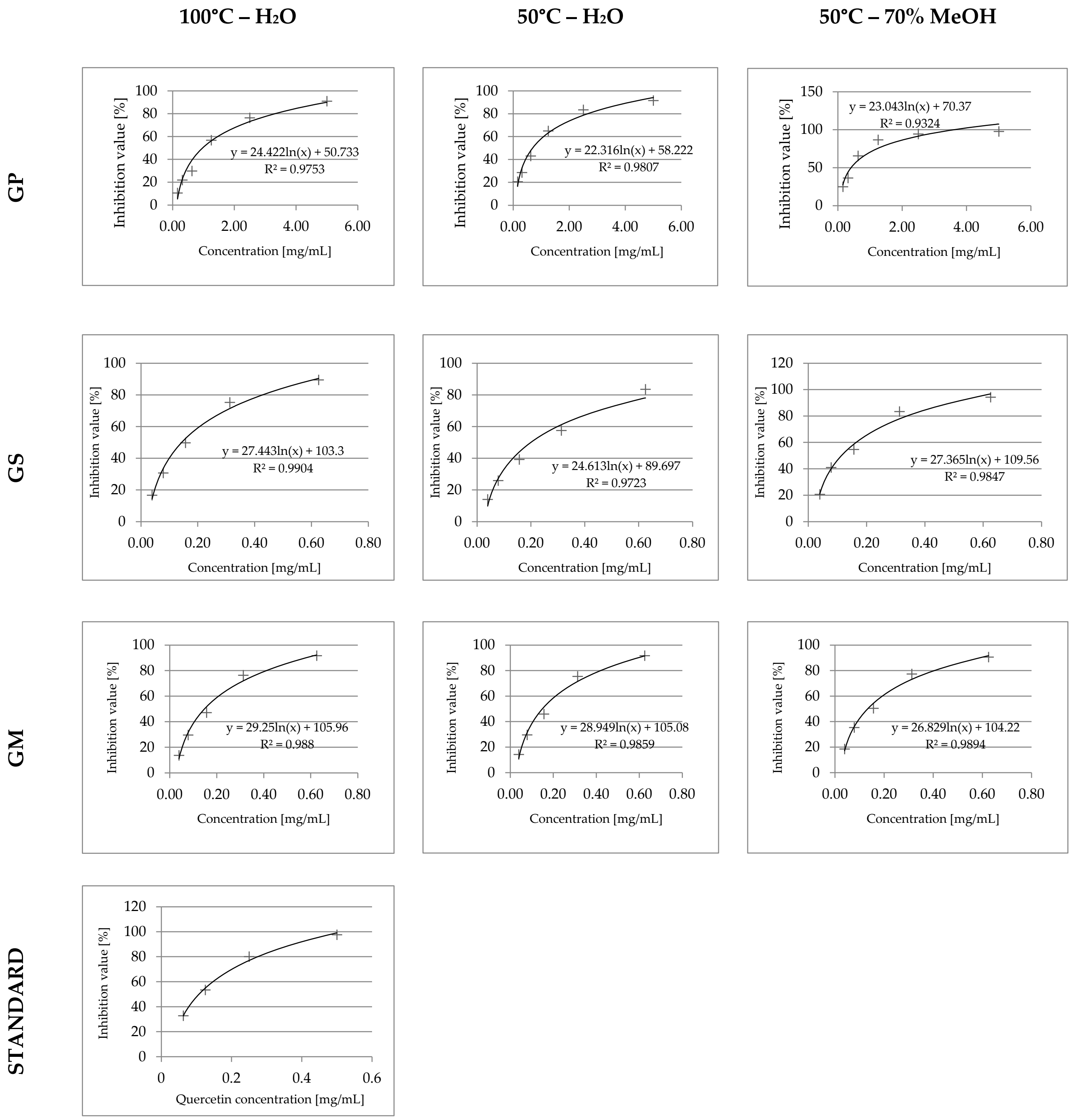

Figure S20. Standard and response curves for **Cu^{2+} chelation assay** (standard: Quercetin; concentration range 0.0625–0.5 mg/mL; y = 31.929ln(x) + 121.24; r = 0.9972).

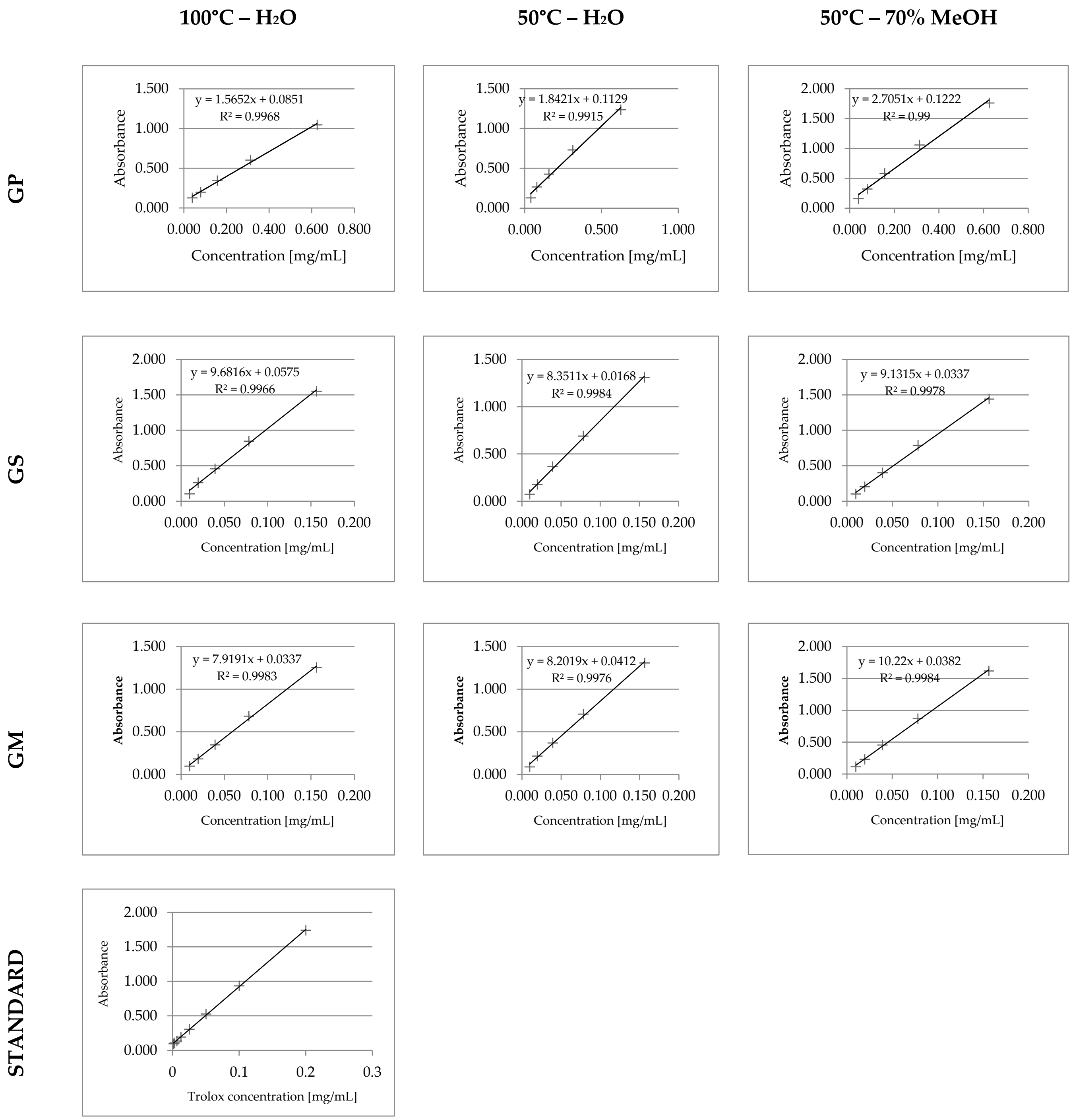

Figure S21. Standard and response curves for **CUPRAC assay** (standard: Trolox; concentration range 0.0156–0.2 mg/mL; y = 8.2943x + 0.0936; r = 0.9998).

Table S5. Extraction yields and sample nomenclature of *Geranium* leaf extracts.

Extract code	Species	Dry extract mass (g)	Extraction yield (%)
GP-H_2O (100 °C)	GP	0.946	18.81
GP-H_2O (50 °C)	GP	0.495	19.80
GP-70% MeOH (50 °C)	GP	1.510	30.18
GS-H_2O (100 °C)	GS	1.412	22.61
GS-H_2O (50 °C)	GS	0.655	26.19
GS-70% MeOH (50 °C)	GS	1.100	21.98
GM-H_2O (100 °C)	GM	1.311	26.12
GM-H_2O (50 °C)	GM	0.558	22.32
GM-70% MeOH (50 °C)	GM	1.810	36.19

GP – *G. phaeum*, GS – *G. sanguineum*, GM – *G. macrorrhizum*; H_2O - water; MeOH - methanol. The extraction method and conditions are described in the Materials and Methods section.
